# Development of Standardized Fetal Progenitor Cell Therapy for Cartilage Regenerative Medicine: Industrial Transposition and Preliminary Safety in Xenogeneic Transplantation

**DOI:** 10.3390/biom11020250

**Published:** 2021-02-09

**Authors:** Alexis Laurent, Philippe Abdel-Sayed, Aurélie Ducrot, Nathalie Hirt-Burri, Corinne Scaletta, Sandra Jaccoud, Katja Nuss, Anthony S. de Buys Roessingh, Wassim Raffoul, Dominique Pioletti, Brigitte von Rechenberg, Lee Ann Applegate, Salim Darwiche

**Affiliations:** 1 Regenerative Therapy Unit, Lausanne University Hospital, University of Lausanne, CH-1015 Lausanne, Switzerland; alexis.laurent@unil.ch (A.L.); philippe.abdel-sayed@chuv.ch (P.A.-S.); aurelie.ducrot@epfl.ch (A.D.); nathalie.burri@chuv.ch (N.H.-B.); corinne.scaletta@chuv.ch (C.S.); sandra.jaccoud@chuv.ch (S.J.); lee.laurent-applegate@chuv.ch (L.A.A.); 2 Preclinical Research Department, LAM Biotechnologies SA, CH-1066 Épalinges, Switzerland; 3 Laboratory of Biomechanical Orthopedics, Ecole Polytechnique Fédérale de Lausanne, CH-2002 Neuchâtel, Switzerland; dominique.pioletti@epfl.ch; 4 Musculoskeletal Research Unit, Zurich Tierspital, University of Zurich, CH-8952 Schlieren, Switzerland; katja.nuss@vetclinics.uzh.ch (K.N.); brigitte.vonrechenberg@uzh.ch (B.v.R.); 5 Children and Adolescent Surgery Service, Lausanne University Hospital, University of Lausanne, CH-1011 Lausanne, Switzerland; anthony.debuys-roessingh@chuv.ch; 6 Plastic, Reconstructive, and Hand Surgery Service, Lausanne University Hospital, University of Lausanne, CH-1011 Lausanne, Switzerland; wassim.raffoul@chuv.ch; 7 Center for Applied Biotechnology and Molecular Medicine, University of Zurich, CH-8057 Zurich, Switzerland; 8 Oxford OSCAR Suzhou Center, Oxford University, Suzhou 215123, Jiangsu, China

**Keywords:** cartilage cell therapy, cell banking, chorioallantoic membrane model, fetal progenitor cells, large animal model, MACI, preclinical research, pilot safety study

## Abstract

Diverse cell therapy approaches constitute prime developmental prospects for managing acute or degenerative cartilaginous tissue affections, synergistically complementing specific surgical solutions. Bone marrow stimulation (i.e., microfracture) remains a standard technique for cartilage repair promotion, despite incurring the adverse generation of fibrocartilagenous scar tissue, while matrix-induced autologous chondrocyte implantation (MACI) and alternative autologous cell-based approaches may partly circumvent this effect. Autologous chondrocytes remain standard cell sources, yet arrays of alternative therapeutic biologicals present great potential for regenerative medicine. Cultured human epiphyseal chondro-progenitors (hECP) were proposed as sustainable, safe, and stable candidates for chaperoning cartilage repair or regeneration. This study describes the development and industrial transposition of hECP multi-tiered cell banking following a single organ donation, as well as preliminary preclinical hECP safety. Optimized cell banking workflows were proposed, potentially generating millions of safe and sustainable therapeutic products. Furthermore, clinical hECP doses were characterized as non-toxic in a standardized chorioallantoic membrane model. Lastly, a MACI-like protocol, including hECPs, was applied in a three-month GLP pilot safety evaluation in a caprine model of full-thickness articular cartilage defect. The safety of hECP transplantation was highlighted in xenogeneic settings, along with confirmed needs for optimal cell delivery vehicles and implantation techniques favoring effective cartilage repair or regeneration.

## 1. Introduction

Demographic shifts of recently increasing amplitude incur heightened incidence and prevalence of chronic degenerative diseases, stimulating translational research eventually providing novel therapeutic solutions toward optimally managing structural tissue repair and functional restoration [1,2]. The developmental bedrock of regenerative medicine continuously contributes to the democratization of cell-based or cell-derived products and therapies, purposed with the prevention and treatment of diverse musculoskeletal affections, ranging from acute traumatic wounds to degenerative wear-related defects. The latter predominate translational research revolving around cartilage tissue repair, as attested to by the emergence of numerous specific cell therapies and combined bioengineered constructs [3,4,5]. Intrinsically poorly equipped for autonomous structural and functional restoration, cartilage tissues often require synergistic combinations of surgical care and therapeutic stimulation for effective repair. In this context, autologous cellular therapy products (i.e., with or without cell culture-expansion) may be proposed and remain historic standards, yet the overarching clinical benefits remain mitigated by multi-factorial inherent burdens (e.g., fibrocartilage scar tissue formation, hypertrophic phenotype preferential induction, relatively onerous cell expansion, excessive phenotypic plasticity in vitro, resort to biphasic surgical interventions, incurrence of donor-site morbidity, or inhomogeneous functional outcomes) [6,7,8,9,10]. The physical and biomechanical properties of cartilage tissue parallelly prompt the development of effective surgical and therapeutic product delivery techniques, as various approaches currently deployed (e.g., microfracture, autologous chondrocyte implantation) may fall short of patient and clinician expectations with regard to outcomes (Table 1). 

Considering native cartilage properties from a technical point of view during cell therapy development, both avascular and alymphatic tissue properties contribute to maintaining a niche, relatively shielded from the recipient’s immune system (i.e., segregation from antigen-presenting cells, dendritic cells, and macrophages). This aspect technically facilitates allogeneic cell therapy approaches aiming for tissue repair stimulation or regenerative processes chaperoning [11,12]. Considering potential therapeutic cell sources (e.g., embryonic or mesenchymal stem cells, bone marrow concentrates, platelet enriched plasma), inhomogeneity and variability impact both safety and potency thereof, hindering effective clinical benefits for cartilage repair [13,14,15,16,17,18,19,20,21,22,23,24,25,26,27,28]. The mitigation of such risks is enabled by the use of cultured primary fetal progenitor cells (FPC), which represent optima in terms of homogenous industrial transposability, regulatory compliance demonstration, on-demand availability, and standardized clinical safety assurance [29,30]. 

**Figure 1:**
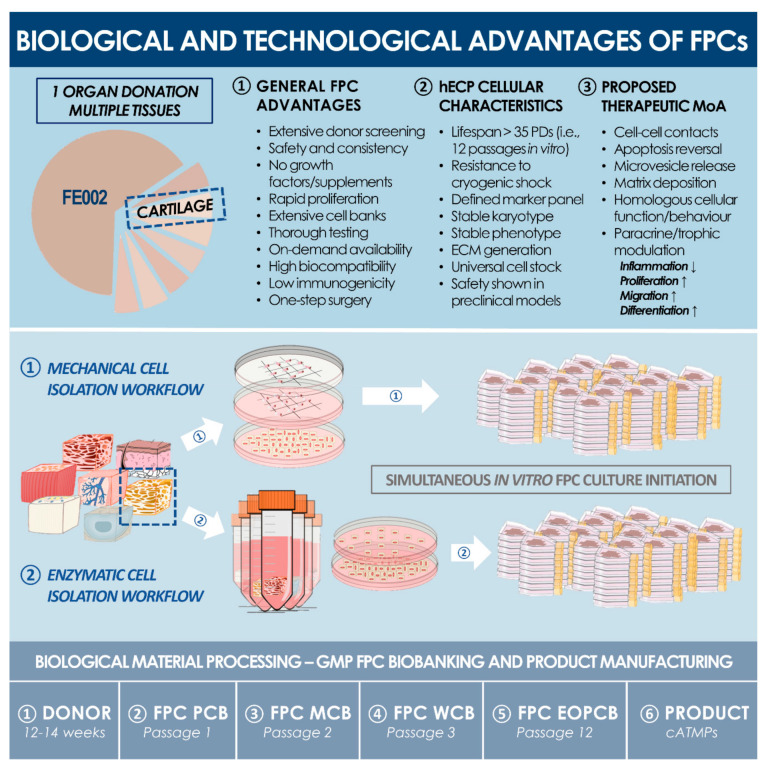
Overview of technological and clinical advantages of processing and using hECPs as therapeutic agents or biotechnological substrates. From a single highly controlled fetal organ donation (i.e., FE002 donation, 2009), various tissue biopsies (e.g., bone, cartilage, intervertebral disc, lung, muscle, skin, tendon, etc.) were bioprocessed for FPC isolation, parallelly using both enzymatic and non-enzymatic methods. Optimized and consistent tissue procurement methodologies, cell culture initiation, and FPC biobanking workflows allow for thorough transversal and longitudinal testing to be performed along the product manufacturing continuum, ensuring optimal quality and safety of end-products. cATMP, *combined advanced therapy medicinal product*; ECM, *Extra-cellular matrix*; EOPCB, *End of production cell bank*; FPC, *Fetal progenitor cell*; GMP, *Good manufacturing practices*; MCB, *Master cell bank*; MoA, *Mechanism of action*; PCB, *Parental cell bank*; PD, *Population doubling*; WCB, *Working cell bank*.

**Figure 2:**
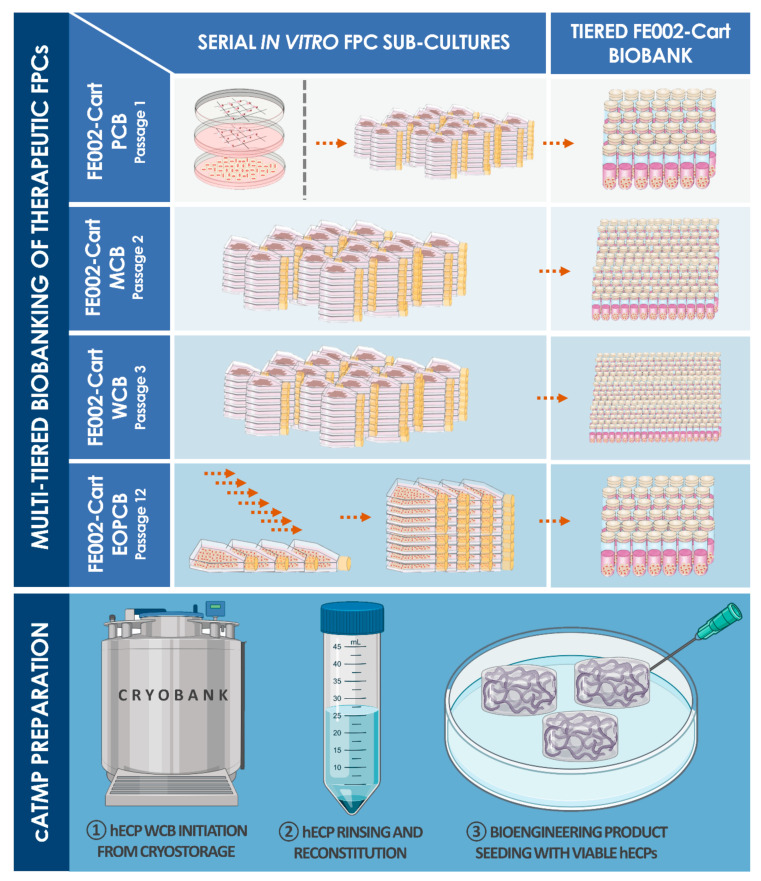
Schematic workflow for optimized and standardized multi-tiered cell banking of primary hECPs (e.g., non-enzymatically isolated FE002-Cart cell type) and cATMP preparation for the clinic. In vitro optimization steps in the pilot expansion study allow one to select the optimally adapted serum manufacturer and lot, two-dimension culture surface size and brand, producing the overall best efficiency of manufacturing. Defined technical specifications and rigorous screening, characterization testing, batch release testing, and safety testing allow for the liberation of highly homogenous, stable, and safe therapeutic biological materials for use in regenerative medicine applications. These considerations and technical specificities are largely dependent on the inherent high robustness, consistency, and stability of properly harnessed FPC sources. Industrial transposition to GMP manufacturing is tangibly attained with such materials, whereas extensive multi-tiered cryopreserved cell banks may be rapidly and efficiently established as proposed herein. Presented bank tiers and passage nomenclature were validated for the FE002-Cart cell type. Off-the-freezer use of such FPC sources enables simple, extemporaneous, and the on-demand production of cATMPs.

Human fetal epiphyseal chondroprogenitors (hECP) constitute robust leads for cartilage cell therapy product development. Such cell sources are characterized by chondrogenic potential relatively superior to that of adult chondrocytes, enhanced phenotypic stability versus stem cells, and low intrinsic immunogenicity or tumorigenicity [11,31,32,33,34,35,36]. Cultured hECPs retain high cellular viability upon appropriate isolation, exhibit extremely robust proliferative potential in monolayer culture at high cumulative population doubling values, maintain homogenous and stable cell population surface marker profiles, and present inherent resistance to multilineage differentiation [34]. They additionally express relatively low levels of hypertrophy markers (i.e., type X collagen), potentially mediated by epigenetic cues in vivo [37,38]. Industrial transposition and the clinical translation of hECP-based products and therapies are of great interest, as a clinical-grade multi-tiered cell bank, established from a single controlled fetal tissue donation, bears the technical potential for generating hundreds of millions of individual off-the-freezer cell-seeded biocompatible constructs for homogenous, standardized, and safe patient treatment (Figure 1 and Figure 2). Properly sourced, isolated, and cultured hECPs, therefore, fulfill the requirements for the development of allogeneic cell therapies, classified in Europe as (combined) advanced therapy medicinal products ((c)ATMPs), as safety, stability, and efficacy of biological starting materials are key drivers upholding the quality standards for product development and implementation [2,30]. 

Operating within harmonized frameworks, such as fetal transplantation programs for FPC sourcing, optimally ensures adequation with high quality and safety prerequisites. Such parameters comprise, without being limited to, the negligible probability of communicable disease transmission, prolonged maintenance of defined and pre-terminally differentiated phenotypes, homogenous proliferation characteristics, low immunogenicity or tumorigenicity, and relatively low technical limitations [39,40,41]. The subsequent validation of identity, purity, sterility, stability, safety, and efficacy of biological raw materials may be robustly implemented when considering the industrial multi-tiered cell banking of hECPs following good manufacturing practices (GMP). The resulting consistent therapeutic cellular materials present high biocompatibility with most approved cell delivery scaffolds, evade or reverse the effects of oxidative stress, and mediate wound healing in a potentially scarless manner [39,40,42,43]. The present study complements our previous reports of stringently optimized processes for hECP cell source selection, primary cell isolation, and bioprocessing methodologies [34]. Industrial transposition and upscaling workflows for hECP multi-tiered banking in view of therapeutic products or processes development following a single fetal organ donation (e.g., FE002, 2009) are presented herein. Such considerations were complemented with preliminary data on the preclinical safety of a specific primary cultured hECP type (i.e., FE002-Cart cell type).

Firstly, optimized cell banking workflows, adaptable to industrial transposition and GMP manufacturing, were proposed, demonstrating the potential generation of millions of safe and sustainable therapeutic products from a single cryopreserved Parental Cell Bank (PCB). The genetic stability of the FE002-Cart cellular materials was confirmed throughout production lots, up to the End of Production Cell Bank (EOPCB). Secondly, clinically relevant hECP doses were tested for preliminary toxicity in a standardized chorioallantoic membrane (CAM) model, a multi-facetted tool for wound and tissue healing evaluation [44]. Lastly, as a first step in defining a novel strategy for cartilage regeneration, a hECP-based MACI-like approach was applied in a three-month good laboratory practices (GLP) pilot safety evaluation in a caprine model of full-thickness articular cartilage defect. Therein, hECPs were delivered within a collagen-based matrix (i.e., cATMP-like prototype), to optimize therapeutic biological material localization, in combination with microfracture (MF), designed to direct new tissue repair and remodeling. Confirming previous results in small preclinical models, the preliminary safety of hECP transplantation was highlighted in a large-animal xenogeneic setting, along with confirmed needs for optimal cell delivery vehicles and implantation techniques favoring effective cartilage tissue repair or regeneration. The absence of adverse reactions (i.e., CAM and caprine models) and marginal indications of enhanced tissue healing in the hECP treatment group at the early three-month timepoint have preliminarily validated the rationale set forth in our transversal FPC transplantation program. Such contributions favor the further translational investigation of the broad therapeutic potential of FPCs and hECPs in particular, synergistically contributing to advancements in musculoskeletal regenerative medicine benefiting patients around the globe. 

## 2. Materials and Methods 

### 2.1. Primary hECP Isolation, Cell Banking, and Characterization

#### 2.1.1. Fetal Tissue Donation and Procurement

The cell source used for this research and preclinical study was the enzymatically isolated FE002-Cart primary FPC type, a hECP source established after standardized processing of a fetal cartilage sample (i.e., 14 weeks of gestation) in 2009. FE002-Cart primary hECPs were isolated using two methodologies (i.e., enzymatic and non-enzymatic cell isolation) from a voluntary organ donation and were further mitotically propagated under appropriate inducive conditions. The FE002 donation was registered under the Swiss federal fetal transplantation program and its dedicated biobank, complying with the laws and regulations within both framework programs. Obtention and use of progeny cells followed regulations of the biobank of the department of musculoskeletal medicine in the CHUV University Hospital (Lausanne, Switzerland). Following anonymous tissue procurement, FE002-Cart hECPs were isolated from the FE002 organ donation according to a validated protocol, as approved by the State Ethics Committee (University Hospital of Lausanne, CHUV, Ethics Committee Protocol #62/07: “Development of fetal cell banks for tissue engineering”, August 2007). One of the cartilage cell sources from this donation, the FE002-Cart primary cell type, was further deposited under the European Collection of Authenticated Cell Cultures accession number N°12070303-FE002-Cart in July 2012.

#### 2.1.2. Primary hECP Enzymatic Isolation and PCB Establishment

In parallel to the non-enzymatic FPC culture initiation, cartilage tissue from the FE002 donation was simultaneously processed for the obtention of an enzymatically isolated FE002-Cart cell type, which served for the banking steps and safety evaluations included in this study. Briefly, a part of the biopsied tissue was separated after reception and rinsed in conserved PBS. The tissue was dissected into <0.3 mm^3^ fragments and placed in sterile centrifuge tubes (50 mL, Falcon^®^, USA) before being immersed in 5 mL of warmed (i.e., 37 °C) trypsin-EDTA (trypsin-EDTA: 0.25% trypsin and 0.1% ethylenediaminetetraacetic acid, Gibco^™^, USA). After an incubation period of 10–20 min at 37 °C, the enzymatic cell dissociation was stopped by the addition of 10 mL of initial cell growth medium. The cell suspensions were then centrifuged at 230 ± 10× *g* at ambient temperature for 15 min. Following supernatant removal, the cellular materials were resuspended in warmed initial cell growth medium. The resulting cell suspension (i.e., excluding the digested remnants of cartilage tissue) was then used to seed six 10 cm diameter cell culture Petri dishes (Falcon^®^, USA). Initial growth medium was added by pipet and the final volume of liquid cell growth medium per vessel was 10 mL. The vessels were stacked and were incubated in a 37 °C ± 2 °C humidified atmosphere with 5% ± 0.5% CO_2_ and 80% ± 10% relative humidity. The initial cell growth medium was composed as described in Supplementary Materials, was exempt of antibiotic supplementation, and was thereafter renewed every other day. Preliminary cell cultures were regularly microscopically assessed to verify adequate cell morphology or growth and to exclude contamination. Towards the end of the initial culture period, abundant fibroblast-type cells (i.e., Passage 0) had populated the culture vessels. Once optimal banking confluency (i.e., 95%, assessed by contrast phase microscopy) was attained for these primary cultures (i.e., 11 days), the cells were passaged to cell culture flasks at a relative viable seeding density of 1.5 × 10^3^ ± 100 cells/cm^2^ and expanded as described above. This cell population (i.e., Passage 1) was then properly harvested, enumerated, and conditioned for cryopreservation as described hereabove in the non-enzymatic cell isolation workflow, constituting the material stock PCB for the enzymatically isolated FE002-Cart cell source. The non-enzymatic hECP cell isolation workflow is presented in the Supplementary Methods.

#### 2.1.3. Pilot hECP Expansion and Cell Banking Campaign

In order to preliminarily assess the quality of the cell type of interest, the enzymatically isolated FE002-Cart cell type was used in a pilot serial expansion campaign in in vitro adherent monolayer culture. This campaign was carried out to validate the PCB lot, to optimize the cell type-specific culture conditions, and to verify the maintenance of biological characteristics throughout cell culture. To this end, PCB vials at Passage 1 were used in a recovery procedure. Briefly, vials to be initiated were transported to the cell culture suite on dry ice (i.e., without direct contact between the vials and the dry ice). Original labels were collected and attached to the relevant batch records. The vials were rapidly thawed in a 37 °C ± 2 °C water bath until the last ice crystal had just disappeared. The cell suspension was then aseptically transferred to a sterile centrifuge tube, before warmed (i.e., 37 °C) culture medium was added, dropwise, to the suspension (i.e., approximately 4 mL of medium for each 10^6^ cells in the tube). After dilution, the cell suspension was centrifuged at 230 ± 10× *g* for 10 min at ambient temperature. After resuspension in warmed growth medium, total and viable cell counts were determined, wherein the acceptance criterion was set at 85% relative viability for the initiation procedure to continue. The cell suspension was then used to seed a variety of culture vessels (i.e., different surface types, surface sizes, and vessel manufacturers). Growth was also evaluated using different FBS lots and manufacturers (i.e., Invitrogen, HyClone™, Sigma^®^, Gibco™), with primary endpoints set for maximized harvest cell yields, optimal proliferative cellular morphology, and minimal population doubling times, in view of attaining maximal manufacturing efficiency. Once the optimal expansion parameters were defined, FE002-Cart cells were serially expanded up to P12 using a relative viable seeding density of 1.5 × 10^3^ ± 100 cells/cm^2^ and cultures were allowed to attain 95% confluency before harvest and passaging. Upon each passage, a fraction of the harvested cells was cryopreserved for characterization assays.

#### 2.1.4. Preliminary Characterization of the Enzymatically Isolated FE002-Cart Cell Type

Specific characteristics of the enzymatically isolated FE002-Cart cell type were studied, such as morphology, expansion kinetics, and lifespan. Cell bank vials at different passages were initiated to obtain standard growth curves, whereas statistically significative differences in cell yields were determined by Student’s *t*-test (i.e., *p* < 0.05). Experiments were conducted in triplicate with three experimental repetitions. In any case, cultures were harvested for enumeration and viability assessment after reaching 80% confluency. Population doubling values (PDVs) and population doubling times (PDTs) at different passages were calculated. The in vitro lifespan of the considered cell type was assessed with regard to passage numbers. The in vitro lifespan was defined as the highest passage at which the growth rates (i.e., expressed as relative cell yields or PDVs) conserved 75% of the initial values (i.e., established as average values over three low passages, P2–P4). Karyology studies (i.e., Giemsa banding) were performed using confluent cell cultures generated from vials at Passages 3, 6, and 12, for the investigation of genetic stability during processing and expansion.

#### 2.1.5. Optimized Multi-Tiered hECP Biobanking Workflow

Following predefined technical specifications and using vials from the PCB material stock, progeny tiered cell banks were derived and established by serial culture expansions of the cell type of interest. To this end, PCB vials (i.e., cells at Passage 1 in frozen state) were used to manufacture master cell bank (MCB) lots at Passage 2 after a culture expansion in one hundred T75 flasks (Nunc^®^, USA), using a relative viable seeding density of 1.5 × 10^3^ ± 100 cells/cm^2^. The cells were processed and preserved as described for the pilot expansions to create the MCB lots. By analogy, serial expansions were performed to establish working cell bank (WCB) lots at Passage 3 and the end of production cell bank (EOPCB) lots at Passage 12. For each manufactured cell bank lot, destined for animal experimentation, sample vials were selected at the beginning, in the middle, and at the end of the batch and were tested for sterility, cell recovery, cell morphology, and growth characteristics (PDV, PDT) upon reinitiation. For each vial lot, a corresponding batch record and certificate of analysis were established, summarizing lot designation, quantity of vials, date of manufacture, tests performed, specifications (i.e., cellular behavior, sterility, quantitative parameters), test results, and release. Non-conforming cell bank lots were destroyed by autoclaving and discarded. Each individual vial was attributed with a code, and storage locations were updated accordingly in a master log. Logbooks were used to record vial movements, ranging from initial deposit to removal for further banking or experimental purposes. Modifications were dated and signed in the logbooks to ensure continued traceability of materials.

### 2.2. Preclinical Safety Study of FE002-Cart FPCs in a CAM Model

#### Study Design

Preliminary toxicity studies of FE002-Cart FPCs were performed on a standardized CAM model. Initially, 58 commercially available fertilized chicken eggs (Animalco AG, Switzerland) were incubated at 37 °C in a humidified atmosphere with the small end facing downward in a dedicated hatching incubator (FIEM srl, Italy). After four days, a small hole was aseptically drilled (Dentalwerk Buermoos drill, Germany) in the small end of each egg, in order to detach the chorioallantoic membrane from the outer shell. The hole was covered with parafilm and the eggs were incubated with the small end upward. On day 10, a large opening (i.e., 25 ± 5 mm in diameter) was created on the small end of each egg using a Dremel^®^ engraver (Bosch Power Tools B.V., The Netherlands), in order to remove the egg shell until reaching the border of the membrane. Six millimeter-diameter O-rings were placed on the membrane of each egg. Eggs which were assessed as non-viable were frozen at −20 °C overnight before being discarded. The large holes were then sealed with parafilm and the eggs were incubated overnight. Eggs which did not survive were frozen the following day before being discarded. On day 11, the membranes were photographed and the test and reference items were then aseptically delivered to the surface of the membranes of each egg. Test items consisted in FE002-Cart cells at Passages 6 and 12, which had been stored as dry pellets at −80 °C and which were reconstituted in sterile 0.9% NaCl (Bichsel AG, Switzerland). To obtain the dry pellets, cell cultures had been harvested as described in the banking procedures, were rinsed with 0.9% NaCl before centrifugation at 230 ± 10 × *g* at ambient temperature for 15 min and the discarding of supernatants. Dry pellets were constituted by 5 × 10^6^ to 40 × 10^6^ cell equivalents, while the test items were constituted by 1.2 × 10^6^ cell equivalents suspended in 250 µL of 0.9% NaCl. Reference items consisted in 250 µL of 0.9% NaCl. Once inoculated, the eggs were sealed with parafilm and incubated again. Endpoint imaging was performed on day 14 using a Leica M205 FA fluorescence stereomicroscope (Leica Camera AG, Germany), as well as standard cameras. Individual viability and quality of blood vessel formation were assessed before the eggs were appropriately frozen and discarded.

### 2.3. Preclinical Safety Study in a Caprine Model

#### 2.3.1. Study Norms and Authorizations

A caprine model was selected for the animal studies based on the strong similarities between caprine and human hyaline cartilage. The study was performed in collaboration with the Musculoskeletal Research Unit (MSRU) of the Vetsuisse Faculty in Zurich under the identification “MSRU0027—Evaluation of a cartilage replacement with epiphyseal chondroprogenitors (ECP) in matrix—An experimental study in goats” with the test items “ECP in Chondo-Gide^®^ matrix over MF”. The animal safety study was performed after proper internal ethical considerations and validation, in compliance with Principles of Good Laboratory Practice (GLP) as set forth by the Organization for Economic Cooperation and Development (OECD), and adopted on 26 November 1997 (C(97)186/Final). Performed procedures had been adapted from specific guidelines, namely the OECD Council Directive 93/42/EEC (June 1993), concerning medical devices and ISO 10993 (i.e., Biological Evaluation of Medical Devices) Part 1 (i.e., Evaluation and Testing, 2009), Part 2 (i.e., Animal welfare requirements, 2006), Part 6 (i.e., Tests for local effects after transplantation, 2007), and Part 12 (i.e., Sample preparation and reference materials, 2007). All animal experiments were conducted according to Swiss federal laws on animal protection and welfare, and were duly authorized by the Zurich cantonal Ethics Committee (License #174/2012).

#### 2.3.2. Animal Study Design

Eight female Saanen goats (Staffelegghof AG, Switzerland), aged 1.50 to 2.25 years and weighing 50–80 kg, identified by earmarks and transponders, were randomized to form two treatment groups. All animals were acclimatized for at least eight days in test conditions. One full-thickness chondral defect of 8 mm in diameter was created in the medial and lateral condyles of the stifle joint in each animal (i.e., two defects per animal). Operated hindlimbs were randomized across the eight animals. Six goats in the “hECP” group (i.e., identifiers ECP1 to ECP6) received hECPs seeded in a Chondro-Gide^®^ collagen matrix scaffold (Geistlich Pharma, Switzerland) surgically placed over induced microfractures (MF) in full-thickness chondral defects (i.e., *n* = 12 defects). Two goats in the “CTRL” group (i.e., identifiers CTRL1 and CTRL2) received sodium chloride-soaked Chondro-Gide^®^ scaffolds (i.e., sham control) over MFs (i.e., *n* = 4 defects). Three months after the surgery, animals were sacrificed and the condyles were harvested. The regions of interest were evaluated by MRI, macroscopically, and histologically. Quality of defect filling, health of surrounding cartilage, subchondral bone reaction, and synovitis indications were described and scored.

#### 2.3.3. Cell-Laden Implant Preparation and Viability Control

To prepare the test items, Chondro-Gide^®^ matrix discs, 8 mm in diameter, were prepared under aseptic conditions in a class A laminar flow hood prior to seeding with hECPs. For the hECP group, approximately 1.2 × 10^6^ hECPs from a dedicated WCB were thawed, rinsed with 0.9% NaCl (Bichsel AG, Switzerland), and seeded onto the rough side of the matrix in 100 μL of 0.9% NaCl. Seeded constructs were kept at room temperature (i.e., 15–25 °C) in parafilm-sealed sterile Petri dishes for an average of 56 ± 18 min (i.e., mean ± SD) prior to surgical implantation by the veterinarian. To validate the construct preparation procedure, hECP cellular viability was verified on a representative sample using an MTT metabolic activity assay (Roche AG, Switzerland), after seeding and incubation at room temperature for two hours in a humidified, sealed Petri dish. The CTRL group received Chondro-Gide^®^ scaffolds seeded with 100 μL of 0.9% NaCl and were maintained under the same environmental conditions as those of the hECP group, with an average of 69 ± 25 min at room temperature in a sealed Petri dish prior to implantation.

#### 2.3.4. Animal Sedation and Anesthesia

Animals were acclimatized at least eight days prior to surgery and fasted 24 h before anesthesia, with access to water. Goats were then sedated with xylazine (i.e., 0.04–0.05 mg/kg_BW_ i.m.) and given preemptive analgesia by the injection of buprenorphine (i.e., 0.004–0.01 mg/kg_BW_ i.m.). Anesthesia was induced with ketamin hydrochloride (i.e., 2.5–6.5 mg/kg_BW_ i.v.) in combination with diazepam (i.e., 0.08–0.13 mg/kg_BW_ i.v.) and propofol (i.e., 0.4–1.4 mg/kg_BW_ i.v.). After local anesthesia of the larynx and intubation, general anesthesia was maintained via the inhalation of isoflurane in oxygen. Cardiovascular monitoring parameters included electrocardiogram, heart rate, and directly measured systolic, mean, and diastolic arterial blood pressure via an arterial catheter placed in an auricular artery. Corneas were protected using ophthalmic ointments.

#### 2.3.5. Surgical Technique

The stifle joint was positioned in maximal flexion during the surgery. An S-shaped cutaneous incision over the joint, followed by incisions parallel to the patellar ligament, exposed both the lateral and medial condyles of the femur. An 8 mm wide circular defect was created in the cartilage tissue and a drill bit or scraper was used to remove cartilage fragments down to the subchondral bone plate. Four microfracture drill holes were created through the subchondral plate and further enlarged using a micro-pick. Fibrin glue (ARTISS^®^ Fibrin Sealant, Baxter, USA) was placed over the microfractures in the subchondral plate and the hECP-laden collagen scaffold or NaCl-soaked collagen scaffold was placed on top, with the rough side toward the subchondral bone plate. Fibrin glue was used to further immobilize the implanted matrix and avoid delamination. After implantation, internal tissues were sutured and staples were used to close cutaneous surgical wounds. The verification of surgical procedure correctness was performed by means of surgery X-rays (i.e., latero-medial and anterior-posterior planes) of operated stifles. X-ray readings also served as a baseline to evaluate changes in the subchondral environment (e.g., subchondral bone cyst formation).

#### 2.3.6. Post-Operative Treatment and Prophylaxis

The post-operative recovery and observation period was set at two hours after the end of the surgical procedure. Post-operative analgesia consisted in buprenorphine (i.e., 0.01 mg/kg_BW_ i.m. or i.v., Temgesic^®^, Indivior Schweiz AG, Switzerland), administered every one to six hours after surgery, depending on visible signs of pain. Carprofen (i.e., 4 mg/kg_BW_ i.v., Rimadyl^®^, Zoetis, USA) was administered once daily for five days after surgery (i.e., including the day of surgery). Prophylactic post-operational antibiotic therapy consisted in penicillin (i.e., 30,000 IU/kg_BW_ i.v.) and gentamycin (i.e., 4 mg/kg_BW_ i.v.) for six days after surgery. Three doses of bisphosphonates (i.e., Bonviva^®^, 3 mg/dose in 500 mL Ringer’s lactate, i.v., Future Health Pharma GmbH, Switzerland) were administered on the day of surgery, and one and two months after surgery, respectively, to prevent subchondral cyst formation and subchondral bone collapse. Operated hindlimbs were maintained in a cast for four weeks after surgery and were further wrapped in soft bandages for two additional weeks. Animals were housed for three months with liberty of movement and access to water, hay, and mineral supplements.

#### 2.3.7. In-Life Monitoring, Sacrifice, and Tissue Processing

Specific medical records were kept for each animal, including all relevant observations. Viability checks and clinical sign observations were performed at least twice daily. Body weight determination was performed once during acclimatization and once during the post-operative housing phase. At the defined three-month timepoint for sacrifice, animals were stunned and spinal cords were promptly sectioned, before standard slaughterhouse processing. Samples from the spleen, liver, lungs, kidneys, stifle joint synovial membranes, popliteal and inguinal lymph nodes (i.e., both hind limbs) were harvested and stored in a stabilizing solution (i.e., 25 mM sodium citrate, 10 mM EDTA, 70% *w*/*v* ammonium sulfate, pH 5.2) to maintain nucleic acid and protein integrity. Organ tissue samples were then preserved at −20 °C in view of subsequent screening for traces of human DNA or potential histological abnormalities. Synovial membranes were further processed, evaluated, and scored.

#### 2.3.8. Stifle Imaging and Analysis

Harvested hindlimbs were transported in an insulated container to perform magnetic resonance imaging (MRI) on a 3.0 T instrument. Coronal sequences were recorded, particularly the T2-weighted Turbo Spin Echo sequence, useful for the detection of subchondral bone sclerosis and the Short T1 Inversion Recovery (STIR) sequence, which suppressed signals from fat tissue and was used to detect bone marrow edema. Analyzed scans showing subchondral bone sclerosis and bone marrow edema were graded using appropriate criteria (Appendix A, Table A6). The depth and width of sclerotic and edema reactions were recorded, as well as the size of the defect. Depth was taken from the surface of the cartilage at the defect site to the farthest permeation of the reaction signal within bone, orthogonal to the defect surface. Width was taken orthogonal to the depth axis, measuring the longest distance. The size of defects was measured at the base of the defects, which could be observed as brighter regions along the cartilage surface, as this area was generally still filled with fluid.

#### 2.3.9. Macroscopic Assessment of Condyles, Histological Processing, and Scoring

Stifle joints were opened and the condyles were exposed for macroscopic evaluation of the state of repair, as well as signs of joint inflammation and notable secondary lesions. The defect (i.e., Zone 0) filling, as well as the health of the surrounding cartilage tissue, both proximal (i.e., Zone 1, defect rim, Zone 2, proximal margin) and distal (i.e., Zone 3, distal margin), were graded using a defect filling score scale and a modified Outerbridge score scale, respectively (Table A1) [45]. Condyles were then separated and randomized, allocating half for MMA embedding. The other half was allocated for decalcification and paraffin embedding. MMA-embedded samples were sectioned to produce 40 µm thick sections, which were surface-stained with Toluidine blue and 3 µm thin sections were stained with Toluidine Blue, and Safranin O/Fast green. Paraffin-embedded samples yielded thin sections (i.e., 3 µm), and were stained with hematoxylin-eosin (HE) and Safranin O/Fast green. A modified O’Driscoll score scale was used to grade the center and rim (i.e., Zones 0 and 1, respectively) of the defect filling based on histological observations (Table A2) [46]. Parameters, such as cellular morphology, Safranin-O staining, surface coverage, thickness, bonding of new tissue to adjacent cartilage, hypocellularity of hyaline-like tissue, and chondrocyte clustering, were evaluated. The Little score scale was used to grade the surrounding osteochondral tissue both proximal and distal to the defect edge (i.e., Zones 2 and 3) based on histological observations (Table A3) [47]. The chondral and subchondral tissues surrounding the defects were evaluated according to structure, cellularity, cell cloning, territorial, and interterritorial Toluidine Blue staining, as well as the tidemark, calcified cartilage, and subchondral bone tissue. When Toluidine Blue staining was not available, as was the case for paraffin embedded-samples, Safranin O staining intensity was used instead.

#### 2.3.10. Synovial Tissue Processing and Evaluation

Samples of synovial fluid from both stifles (i.e., operated and contralateral) were taken and stored for further processing to detect biochemical signs of joint inflammation and degeneration. Synovial membranes of both stifle joints were also harvested, fixed in 4% formalin, dehydrated, embedded in paraffin, sectioned, and stained with HE. Synovial membrane sections from both the operated and contralateral control stifles were used to grade synovitis according to adapted scoring scales described previously, the Krenn score scale and a modified Rooney score scale (Table A4 and Table A5, respectively) [48,49]. Indications of synoviocyte hyperplasia, increased resident cell density, fibrosis, angiogenesis, and inflammatory infiltrates were evaluated. Synovial membrane samples from unoperated contralateral stifles were also evaluated to provide an internal control. Staining for human DNA to detect hECPs within goat synovial membranes was achieved using Ventana’s Alu Positive Control Probe II (Roche AG, Switzerland) and counterstaining with Nuclear Fast Red.

#### 2.3.11. Statistical Analysis

Data in histograms are represented as means, with error bars showing standard deviations. Differences between the hECP and CTRL groups were analyzed statistically using Student’s *t*-test, where *p* < 0.05 indicated statistically detectable differences.

## 3. Results

### 3.1. hECP Isolation and Multi-Tiered Cell Banking

**Figure 3:**
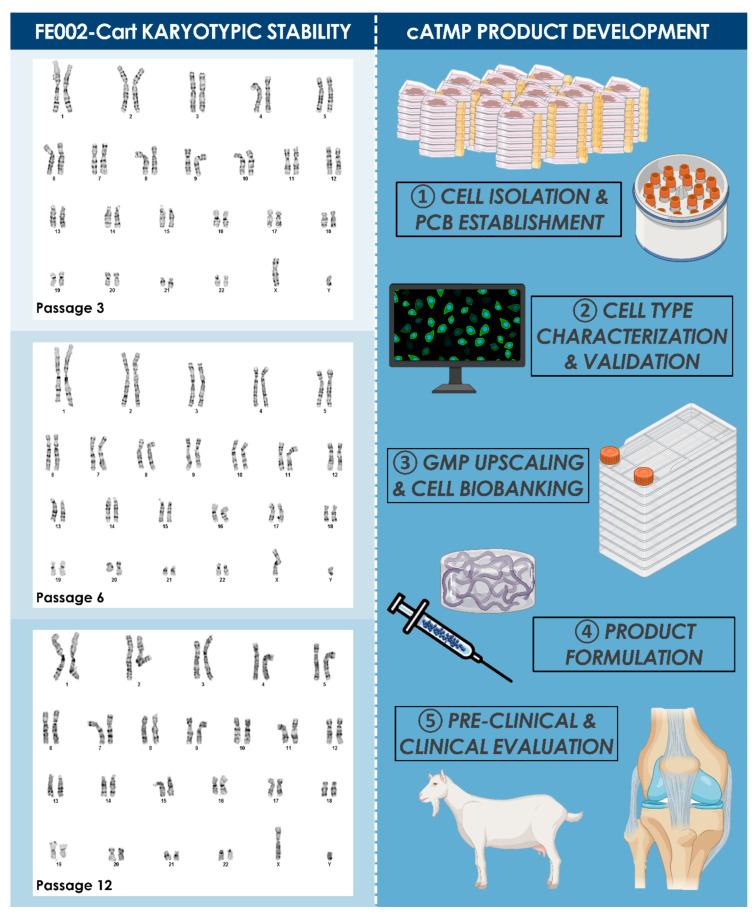
Karyotypic stability of the FE002-Cart cell type (i.e., Passages 3, 6, and 12) and essential steps in view of cATMP development with standardized FPC biobanks under the Swiss Fetal Transplantation Program. A normal 46,XY karyotype was observed in all test-conditions using Giemsa banding and was characterized as stable throughout the considered passages. In the product development workflow, robustness of biological material sourcing and processing are prerequisites for transpositional and translational success. Thorough characterization of material stability and safety demonstration are furthermore imperative requirements in view of constituting investigational medicinal product dossiers (IMPD) and investigators brochures (IB) for human clinical trials. The high homogeneity and stability of the FE002-Cart cell type enables optimal integration of such biological active pharmaceutical ingredients (API) in product developmental processes.

In view of R&D and clinical applications, the FE002 tissue donation differential bioprocessing and parallel FE002-Cart PCBs establishment was performed in the ad hoc CHUV accredited manufacturing suite, as defined under the Swiss Fetal Transplantation Program (Figure 1). Extensive manufacturing optimization and characterization steps, performed during the pilot cell banking phase, enabled the efficient establishment of large and consistent MCB, WCB, and EOPCB vial lots (Figure 2). Optimal endpoint cell expansion manufacturing yields were obtained with Sigma^®^ or Gibco^™^ FBS lots, and 75 to 175 cm^2^ standard uncoated Nunc^®^ culture flasks. The cell banking nomenclature and tier type depended on the passage number (Pn) characterizing the cell lot and the cell type of interest. Cells in cryovials from the PCB were defined as belonging to P1 in their frozen state, becoming P2 upon re-initiation and subsequent culture-expansion. With vial contents ranging between 10^6^ and 10^7^ viable cells at the time of freezing, typical manufacturing lot sizes approximately reached 80–100 vials (i.e., PCB, P1), 80–100 (i.e., MCB, P2) and 100–150 vials (i.e., WCB, P3). The process of multi-tiered cell banking of the FE002-Cart cell type, as described herein, was completely repeated and validated at least five times. Further sub-tiering of the WCBs into 3 tiers (i.e., WCB tier 1 at P3, WCB tier 2 at P4, and WCB tier 3 at P5) enabled the establishment of a sustainable model for cartilage FPC bank exploitation, virtually abolishing the need for repeated organ donations. Release testing was performed for FE002-Cart parental, master, and working cell banks, while in vitro and in vivo safety testing assays were performed on WCB and EOPCB materials. Screening tests that were accomplished on the EOPCB (i.e., cells at Passage 13 after recovery), confirmed the maintenance of a normal karyotype and demonstrated the absence of toxicity. The sterility of culture conditions was also confirmed at each banking step. Karyotyping was accomplished in three conditions (i.e., P3, P6, and P12) using Giemsa banding to assess cell genetic stability throughout passages, wherein 60 to 100 metaphases were examined each time. No polyploidy or clonal abnormalities were observed, and no chromosome aberrations were noted on the normal 46,XY karyotype (Figure 3). Cell morphology during expansion was consistently fibroblastic in nature (i.e., spindle-shaped cells, elongated) and characteristic for this specific cell type. For each expansion passage from Passage 3 to Passage 12, the cell viability, as assessed during the recovery studies, ranged from 94% to 100%, confirming a high resistance to cryopreservation. Mean PDT values ranged from 68 to 89 h (i.e., P2-68 h; P3-73 h; P4-79 h; P5-80 h; P6-78 h; P7-74 h; P8-77 h; P9-82 h; P10-86 h; P11-84 h; P12-89 h; data from FE002-Cart EOPCB establishment). Mean PDV data confirmed that the lifespan, as defined previously, was at least equal to 12 standard passages for the FE002-Cart cell type.

### 3.2. Safety of hECPs in the CAM Model

**Figure 4:**
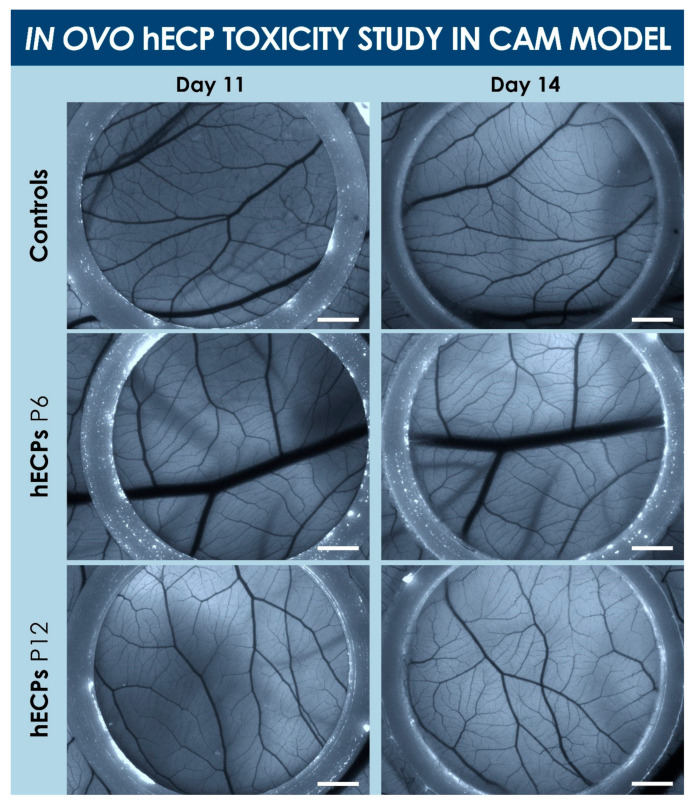
Representative data overview of standardized CAM experiment for evaluation of hECP embryotoxicity and angiotoxicity in ovo. Data are presented for eggs N°10, N°32, and N°12 for control, P6, and P12 groups at days 11 and 14, respectively. Scale bars = 1 mm.

Results indicated that hECP test items did not promote mortality or abnormal formation of vasculature in the CAM model (Figure 4 and Figure S1). Initially (i.e., day 0), 58 eggs were incubated after procurement. At the time of the large opening creation (i.e., day 10), 47 eggs were assessed as viable, with 44 surviving eggs on day 11. The control, P6, and P12 groups were allocated with 14, 15, and 15 eggs, respectively. At the time of endpoint analysis (i.e., day 14), 12, 14, and 15 eggs were assessed as viable in the control, P6, and P12 groups, respectively. It is worth noting that all eggs which did not survive between days 11 and 14 were slightly damaged at the time of large opening creation, with minimal loss of vasculature integrity. The effect of the test-items on egg viability was, therefore, determined to be non-significative at day 14. Macroscopic and microscopic endpoint observation did not reveal an impact of test-items on the formation of blood vessels in the experimental model, or any other sign of development perturbation.

### 3.3. Safety of hECPs in the Caprine Cartilage Defect Model

#### 3.3.1. General Observations

No test item-related mortality was observed. Neither test item-related clinical signs nor test item-related changes in examined physiological parameters were observed. No test item-related neurological signs or changes in body weight were observed. No lameness or other apparent abnormal clinical symptoms were observed in any of the goats prior to sacrifice at three months.

#### 3.3.2. Implant Preparation and Surgical Procedure

**Figure 5:**
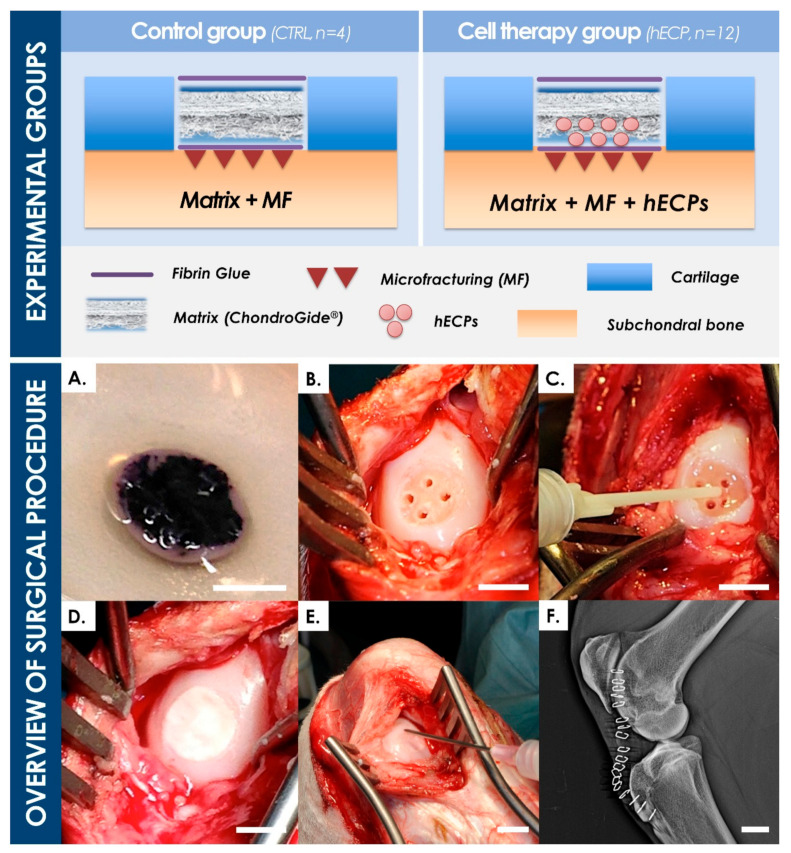
Caprine safety study design showing the artificial defects in the CTRL group receiving Chondro-Gide^®^ matrix, loaded with normal sterile saline, and glued over the microfracture drill holes with fibrin sealant. The hECP group received the same matrix, seeded with 1.2 million freshly thawed hECP cells suspended in saline, glued over microfracture drill holes with fibrin sealant. In both cases, fibrin sealant was also applied on top of the membranes, to improve fixation. (**A**) Seeded constructs displayed strong purple staining of seeded cell (i.e., indicating metabolic activity in the MTT assay) after two hours of incubation at room temperature, in the same conditions as those followed prior to implantation. (**B**) The surgical procedure comprised creation of four microfracture drill holes within the created defect, through the subchondral bone plate. (**C**) Fibrin glue was then added to the bottom of the defect filling and covering the created microfracture drill holes. (**D**) The bioengineered construct was then implanted in the defect, with the rough side facing downward and the smooth side facing toward the surface. (**E**) Fibrin glue was additionally deposited on top of the construct membrane to reduce the subsequent risk of delamination. (**F**) Suturing of the capsule was performed, and soft tissue was replaced back together, before control X-rays were performed to evaluate the surgical procedure and to establish a baseline (i.e., monitor the potential subchondral bone cyst formation). Imaging represents the operative and post-operative procedures for medial condyles of ECP1 (i.e., **B**,**D**), ECP2 (i.e., **C**), and ECP4 (i.e., **E**,**F**). Scale bars = 5 mm (**A**); 6 mm (**B**–**E**); 12 mm (**F**).

Seeding of the collagen-based implants with the ad hoc hECP suspension was rapid and efficient. All prepared implants conserved their macroscopic properties between the time of manufacturing and application to the defects. The viability and localization of the hECPs in the constructs were confirmed as maintained by the MTT assay, as viable cells could be detected by the Formazan as dark purple precipitates, which indicated metabolic activity and homogenous cell distribution on the seeded matrices (Figure 5A). As the viability validation was performed 2 h after cell seeding, it could be safely concluded that cells on implanted scaffolds were also viable, as constructs were all implanted well within the 2 h window (i.e., 56 ± 18 min) after seeding. The successive steps of surgical implantation of the bioengineered constructs were confirmed to be adapted for treatment of the created lesions, wherein no technical difficulties were noted during creation and treatment of said defects (Figure 5B–E). Post-operative X-rays confirmed the absence of observable abnormalities or artefacts in considered joints (Figure 5F).

#### 3.3.3. Macroscopic Observations and Scoring of Cartilage Repair

**Figure 6:**
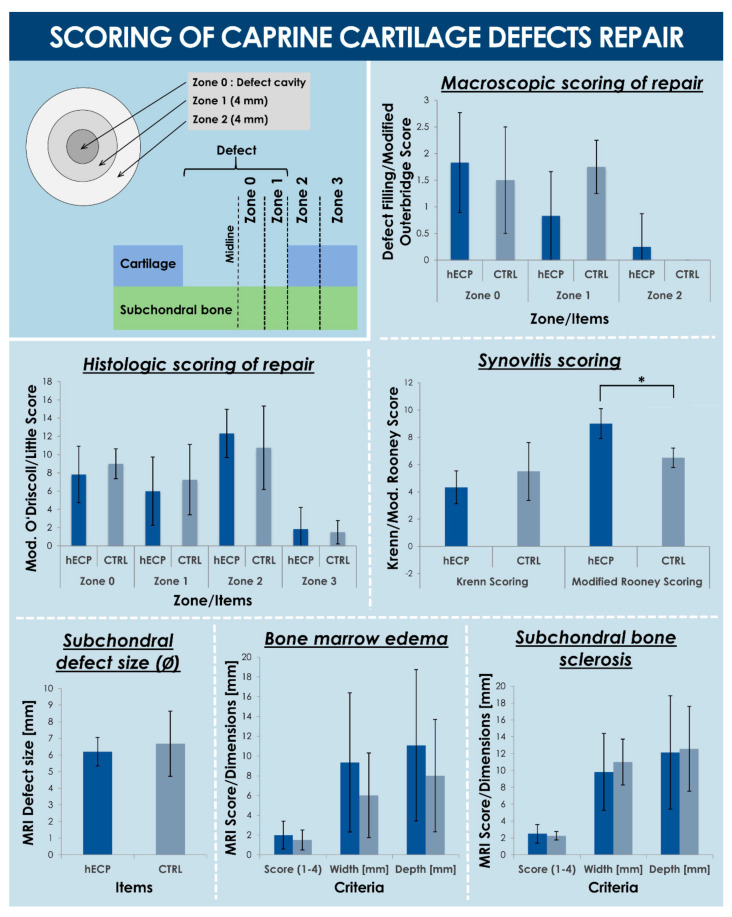
Evaluation and scoring of caprine cartilage repair after three months. The macroscopic state of the defect filling (i.e., Zone 0) was graded, as well as the health of cartilage tissue immediately surrounding the defect zone within 4 mm (i.e., defect rim, Zone 1), the tissue beyond the 4 mm demarcation (i.e., adjacent margin, Zone 2), and the surrounding tissue (i.e., distal margin, Zone 3). Macroscopic repair scoring was performed using the defect filling score and a modified Outerbridge score scale. Histologic scoring of repair was performed using a modified O’Driscoll score scale (i.e., for Zones 0 and 1) and the Little score scale (i.e., for Zones 2 and 3), respectively. Synovitis scoring was performed using the Krenn score scale and a modified Rooney score scale. Measurement and scoring of endpoint defect size, bone marrow edema, and subchondral bone sclerosis were performed based on post-sacrifice MRI data. Data are presented as mean values with standard deviations as error bars. (*) *p* < 0.05.

All tissues of interest were evaluated directly upon post-sacrifice surgical opening of the joints (Figures S2 and S3). Grading the defect filling (i.e., macroscopic repair in Zone 0, defect filling score, Table A1) did not show a detectable difference between the hECP group (i.e., mean score value of 1.83 ± 0.94) and the CTRL group (i.e., mean score value of 1.50 ± 1.00) (Figure 6, Table S1). Similarly, the health of cartilage tissue around the defect edge (i.e., macroscopic repair in Zone 2, proximal margin, Modified Outerbridge Score, Table A1) was also equivalent in the hECP group (i.e., mean score value of 0.25 ± 0.62) and the CTRL group (i.e., mean score value of 0.00 ± 0.00) (Figure 6, Table S1). However, the health of the cartilage tissue immediately surrounding the implant site (i.e., Zone 1, defect rim, consisting in a 4 mm diameter rim, defect filling score, Table A1) appeared to be improved in the hECP group (i.e., mean score value of 0.83 ± 0.83) compared to the CTRL group (i.e., mean score value of 1.75 ± 0.50), with a nearly statistically detectable difference (i.e., *p* = 0.06) (Figure 6, Table S1). Indeed, the presence of hECPs appeared to have maintained the health of the cartilage rim around the defect (Figure S2). Macroscopic observations were complemented by biomechanical analysis of the harvested tissues, which showed, despite elevated heterogeneity and variability upon analysis, a trend toward early formation and maintenance of structurally sound tissues at the early three-month timepoint (Supplementary Data).

#### 3.3.4. MRI Findings and Scoring

Post-harvest MRI data of sclerotic reaction and bone marrow edema seemed, for the most part, to map what was observed macroscopically, except for a few cases (Figures S14 and S15). It was interesting to note that, much like macroscopic signs, the condyles seemed to be isolated in their reaction, acting almost like independent sub-compartments within a same stifle joint, as seen in CTRL1 where the medial and lateral defects scored the highest and nearly lowest, respectively, with regards to subchondral bone defect size (Figure 6, Figures S14, and S15). Statistical analysis of the MRI grading did not detect a difference between hECP and CTRL groups, with the hECP group (*n =* 12) scoring, on average, 2.50 ± 1.09 and the CTRL group (*n* = 4) scoring, on average, 2.25 ± 0.50 in sclerotic reaction (Figure 6, Table S8). The hECP group also scored, on average, 2.00 ± 1.41 and the CTRL group scored, on average, 1.50 ± 1.00 for bone marrow edema (Figure 6, Table S8). Other imaging measurements were also statistically indistinguishable between the two groups. The hECP group did, however, trend toward narrower sclerotic reactions (i.e., 9.83 ± 4.56 mm and 11 ± 2.72 mm width dimensions for hECP and CTRL groups, respectively), while the CTRL group showed a mild trend toward narrower (i.e., 9.35 ± 7.05 mm and 6.02 ± 4.28 mm width dimensions for hECP and CTRL groups, respectively) and shallower (i.e., 11.08 ± 7.66 mm and 8.02 ± 5.68 mm depth dimensions for hECP and CTRL groups, respectively) bone marrow edema (Figure 6, Table S8).

#### 3.3.5. Histological Observations

Upon assessing histological evaluations, no statistical difference was detected between hECP and CTRL group condyles. Indeed, scoring the defect center (i.e., Zone 0) according to the modified O’Driscoll score scale (Table A2) revealed no statistical difference between the hECP group (i.e., mean score value of 7.83 ± 3.10) and the CTRL group (i.e., mean score value of 9.00 ± 1.63) (Figure 6, Table S4). Scoring the defect rim (i.e., Zone 1) according to the modified O’Driscoll score scale (Table A2) revealed no statistical difference between the hECP group (i.e., mean score value of 6.00 ± 3.74) and the CTRL group (i.e., mean score value of 7.25 ± 3.86) (Figure 6, Table S5). Scoring the adjacent margin (i.e., Zone 2) according to the Little score scale (Table A3), revealed no statistical difference between the hECP group (i.e., mean score value of 12.33 ± 2.64) and the CTRL group (i.e., mean score value of 10.75 ± 4.57) (Figure 6, Table S6). Scoring the distal margin (i.e., Zone 3) according to the Little Score (Table A3) revealed no statistical difference between the hECP group (i.e., mean score value of 1.83 ± 2.37) and the CTRL group (i.e., mean score value of 1.50 ± 2.29) (Figure 6, Table S7). Looking at the defect filling and the subchondral bone region under the defects, CTRL condyles exhibited normal subchondral bone, a calm unreactive scar with mature fibrous tissue (CTRL1, Figure S12), as well as an indication of fibrous and mesenchymal tissue and blood filling of the subchondral trabeculae (CTRL2, Figure S13). There was also an instance of a blood clot covering the defect with granulation tissue deep into subchondral bone, a detectable subchondral cyst filled with vessels, granulation and fibrous tissue, some signs of fibrocartilage formation, and increased remodeling on bone interfaces, indicative of a resorptive active inflammatory status with a reactive enzymatic process (CTRL1, Figure S12). The chronically active inflammatory response was also seen in CTRL2 with granulomatous inflammatory tissue, neutrophils, foreign body giant cells and some mature fibrous tissue (CTRL2, Figure S13). Subchondral bone cysts with undifferentiated mesenchymal tissue and/or newly formed fibrous tissue were also a common observation in the hECP group (ECP1, Figure S6; ECP2, Figure S7; ECP3, Figure S8; ECP4, Figure S9; ECP5, Figure S10). There was also evidence of undisturbed bone marrow with no notable new bone formation, no cysts and no increased remodeling in some cases (ECP6, Figure S11) where the other condyle showed very different signs, such as a cyst-like fluid-filled area, immature granulation tissue with many vessels, and immature chondrocytes present with observed unresorbed remnants of bone, hyperemic tissue with areas of necrosis, and indications of a chronic active granulomatous inflammation (ECP6, Figure S11). Finally, some subchondral reactions showed signs of normal subchondral tissue, active islets of osteoblasts, a slight amount of fibrous tissue, and no remaining granulation tissue with the clear formation of bone marrow in detectable islets of hematopoiesis (ECP1, Figure S6).

#### 3.3.6. Synovial Membrane Analysis and Synovitis Scoring

The synovial membrane samples taken from operated stifles, as well as contralateral control stifles, were analyzed for indications of inflammation and fibrosis (Figure S4). Scoring synovitis, according to the Krenn synovitis score scale (Table A4), revealed no statistical difference between the hECP group (i.e., mean score values of 4.33 ± 1.21 and 2.00 ± 1.26 for operated and unoperated limbs, respectively) and the CTRL group (i.e., mean score values of 5.50 ± 2.12 and 1.00 ± 0.00 for operated and unoperated limbs, respectively) (Figure 6, Table S2). Scoring synovitis, according to the modified Rooney score scale (Table A5) revealed no statistical difference between contralateral control limbs in the hECP group (i.e., mean score value of 3.50 ± 1.52) and the CTRL group (i.e., mean score values of 3.50 ± 2.12) (Figure 6, Table S3). It did, however, indicate a relatively lower synovitis score (*p* = 0.026) in the CTRL group, when comparing operated limbs between the hECP group (i.e., mean score value of 9.00 ± 1.10) and the CTRL group (i.e., mean score value of 6.50 ± 0.71) (Table S3). It is important to note that synovial membrane samples from operated stifles in CTRL1 showed granulation tissue formation, while samples from the operated stifle in CTRL2 showed indications of active severe granulomatous inflammation with multinucleated cell infiltrates.

#### 3.3.7. hECP Cell Tracking

When tracking hECP cells within synovial membrane samples, ECP5 and ECP6 showed inclusions. Those found in ECP6 were scarce, concentrated in tissue creases, and mixed with caprine cells. The human ECP cells found in synovial membranes of ECP5 both presented clump patterns, as well as sparse and diffuse patterns. Human cells did not establish exclusive clumps, however, or appear to segregate away from caprine tissue. In fact, especially regarding the sparse single cells found in ECP5 membranes, human cells that had engrafted seemed integrated with the surrounding tissue. No human cells were observed in the synovial membranes of the un-operated contralateral limbs in ECP5 and ECP6, as well as operated and contralateral stifles in ECP1, ECP2, ECP3, and ECP4. While this effect may have been an isolated occurrence in ECP5 and ECP6, it was also deemed likely that inclusions did not occur everywhere in the membrane, and that tissue excision from some sites might have missed other sites where inclusions might have occurred (Figure S5).

## 4. Discussion

### 4.1. Unsatisfactory Surgical State of the Art for Cartilage Repair

Articular cartilage tissue, upon sustaining damage or during the course of long-term degenerative affections, is intrinsically highly restricted in terms of structural repair capacity and functional restoration, due to an avascular nature and relative resident cell scarcity. When left untreated, cartilage defects may lead to total joint degeneration and may eventually lead to post-traumatic osteoarthritis (OA). Palliative treatment of cartilage injuries and defects, such as analgesic pharmacotherapy, controlled ponderal loss, physiotherapy, and viscous supplementation may address and minimize the pain sensation. Nevertheless, such interventions fail to treat the root-cause or repair the lesion itself. Such conservative measures have been shown to provide limited and momentary symptomatic relief, which may not delay tissue degeneration and cartilage defect development. Therefore, surgical management options have constituted the leading therapeutic strategies over the last few decades for the effective repair and prevention of extensive cartilage lesions, wherein cell therapies and tissue engineering-assisted surgeries have been largely introduced.

In this context, bone marrow stimulation or microfracture (MF) is currently the most widely applied surgical technique for the repair of cartilage tissue injuries. Despite the relatively rapid formation of repair tissue, specifically enabling swift returns of professional athletes to competitions, concerns have been raised around the effective long-term outcomes. Indeed, baring adverse complications, the MF procedure leads to the formation of fibrocartilaginous scar tissue, which is mechanically inferior to, and integrates poorly in, the surrounding hyaline cartilage. Therefore, the resulting state of repair deteriorates with time and the procedure loses its effectiveness as a function of the biological age of patients [50,51]. As a corollary to the previously mentioned intrinsic physiological limitations within dense hyaline cartilage tissues, access to blood-borne reparative cues and related mechanisms is limited. MF attempts to strategically overcome this barrier by artificially breaching the subchondral bone plate, causing it to bleed into the cartilage defect. Thereafter, a major pitfall resides in the instability of the resulting blood clot. Upon clot detachment or incomplete void filling of the defect cavity, the intended mechanism of repair is compromised, potentially severely impacting the clinical outcome.

Notwithstanding the technical simplicity of MF, the overall resulting effects are limited to mere resurfacing of cartilage defects, accompanied by formation of a fibrocartilaginous scar and unstable mesenchymal blood clot. High clinical needs, therefore, drive the development of emerging tissue engineering strategies and cell therapy applications to repair and restore cartilage. Therein, the overarching objective resides in implementing a therapeutic treatment capable of generating, or stimulating the generation of, hyaline-like matrix with restored low friction properties and optimal integration in recipient tissues. As observed during the pilot safety study in the caprine model described herein, the choice of surgical approach and cell therapy delivery method is of prime importance, along with the choice of active cells, to favor optimal therapeutic outcomes.

### 4.2. Limited Available Cell Therapies for Cartilage Regeneration

Key features directing the design and development of novel cell-based treatments for cartilage injuries comprise on-demand availability to clinicians and the reliability of potent therapeutic effects. These requirements contrast with modalities of currently used autologous chondrocyte treatments, which comprise long-term planning phases, onerous and lengthy GMP therapeutic batch manufacture, and multiple surgeries. This is particularly outlined in the case of autologous chondrocyte implantation (ACI), falling short of MF with regard to recorded procedure counts, despite the strong developmental drive behind the successive generations of ACI therapies and the inherent limitations of MF [50,52]. These considerations prompt the development of effective combinations of therapeutic biologics, conjugated to appropriate constructs to be surgically delivered, enabling an effective appraisal of novel regenerative products and a phasing-out of historical yet subpar therapeutic options.

Cartilage tissue engineering strategies have evolved since the initial reports of ACI by Brittberg et al. in 1994 for condyle lesion treatment, wherein autologous chondrocytes were injected into the defect under a periosteal patch. Despite relatively improved outcomes, ACI requires two-stage surgeries and incurs high economic burdens, restricting its application to young patients with symptomatic ICRS grade III to IV lesions (i.e., usually >2 mm in size). Long-term follow-ups of first generation ACI have shown that the implantation of autologous cultured chondrocytes with bone marrow stem cell (BMSC) or BMSCs alone resulted in equivalent improvements in reported outcomes, with no apparent tumor formation risk. Overall, such cell therapies prevented total knee replacement needs and limited subsequent or repeat surgeries [53]. Matrix-induced autologous chondrocyte implantation (MACI) includes patient-specific therapeutic cells seeded on an appropriate matrix (e.g., Chondro-Gide^®^, Geistlich Pharma, Switzerland). MACI was developed as an improved version of ACI, involving the use of isolated autologous chondrocytes. On the other hand, AMIC (autologous matrix induced chondrogenesis) does not require the extraction of autologous cells. A membrane is simply placed on top of the MF site to seal and stabilize the blood clot and provide a scaffold for bone marrow cells to populate the defect. Various approaches were adopted in several formats for commercially available products (e.g., Hyalograft^®^ C, Anika Pharmaceutics, USA; BioSeed^®^-C, BioTissue, Germany; Novocart^®^ 3D, TETEC, Germany; CaReS^®^, Arthro-Kinetics, Austria; NeoCart^®^, Histogenics, USA; Chondrom^®^, Regrow, India; Chondrosphere^®^, Co.don AG, Germany) [54]. Although debatable, it is argued that collagen membranes may modulate the kind of repair tissue in formation, significantly improving clinical outcomes, and providing results superior to MF alone [55,56]. A recent systematic review on MACI treatments for chondral defects reported between 1998 and 2016 identified 38 clinical studies with 12–180 months of follow-up, involving 1614 patients to treat 1755 lesions [57]. Despite heterogeneity and variability (i.e., clinical scores, scaffold type, lesion localization-based outcomes), MACI was shown to be safe, effective, and satisfactory for functional recovery [58,59]. The various regenerative medicine approaches cited hereabove for cartilage repair may be distinguished by degrees of regulatory complexity. While MF is almost purely surgical (i.e., no implanted material), AMIC introduces a membrane on top of MF (i.e., no added cells), and ACI in its various generations including MACI include autologous chondrocytes and some form of biomaterial (e.g., collagen scaffolds).

Besides autologous chondrocytes, various cell sources have been investigated for cartilage bioengineering. Alternative biologic therapeutic material sources have been considered for conjugation with post-MF matrix-stabilizing techniques and regenerative response eliciting through bone marrow stimulation, such as platelets [60]. Such approaches remain hindered by potentially increased risks of subchondral cyst formation in reaction to synovial fluid pressure, following secondary adverse subchondral bone plate micro-breaching. Novel autologous cell therapy prospects comprise cultured nasal septum chondrocytes in collagen membranes to develop 30 × 40 × 2 mm tissue grafts for a nose-to-knee approach, yielding solid evidence of optimal clinical effectiveness and sustainability [61]. Allogeneic cartilage cell therapies have also been investigated, allowing for single-step surgeries in widened patient age brackets, early management of degenerative joint disease, and strong prophylactic potential [62]. Notably, polydactyly-sourced chondrocytes in a 3:1 mixture with irradiated counterparts transfected to express TGF-β1 (i.e., INVOSSA™ K injection, Kolon Life Sciences, Korea) were proposed as a self-stimulating regenerative strategy [63]. Strikingly, the corresponding phase III clinical study (i.e., Clinicaltrials.gov identifier NCT03383471) involving grade 3 chronic degenerative joint disease in the knee was interrupted due to an apparent mis-labeling of the non-irradiated cell substrates, which were subsequently identified as fetal kidney cells (i.e., GP2-293 cell line). The trial resumed after one year of interruption, in May 2020, and marked the first use of a cancerous cell line for human therapeutic application, in which long-term safety will require elucidation, despite irradiation of the cellular materials. The indirect therapeutic use of substrate fetal kidney cells is of great interest in this case, as such materials have benefitted from extensive industrial track records since the 1950s in vaccine industrial manufacturing. The GP2-293 cells are in fact a transfected cell line derived from the HEK-293 cell line currently used for COVID-19 vaccine development by Regeneron, USA. The HEK-293 is a historic fetal kidney cell line that was developed in 1973 after an elective abortion in the Netherlands and was a clone resulting from adenovirus 5 DNA transfection, establishing a complex karyotype for the specified cell source. Such considerations resonate with the rationale set forth in the Swiss fetal progenitor cell transplantation program, wherein we have developed and optimized therapeutic cell sources for tissue-specific regenerative medicine solutions (e.g., FE002-Cart hECPs) and biotechnological applications.

### 4.3. Cultured hECPs as Optimal Candidates for Cell-Based Cartilage Repair

Cultured hECPs provide the stability, reliability, and traceability required from a source material for allogeneic cell therapy applications [34,40]. Such cell types present relatively high phenotypic stability, potent intrinsic chondrogenesis, and stimulation thereof (i.e., high levels of sulfated GAGs, high Sox9:Scleraxis ratios, high *IHH* and *PTH1R* expression, TGF-β3-induced production of aggrecans, and high contents of types I and II collagen) [12,64]. In terms of functionality (i.e., reparation stimulation or chaperoning role) evolution and dependency to processing workflows, the FE002-Cart cell source had been studied to validate stability thereof, wherein chondrogenic potential was assessed as stable between 9 and 18 population doublings [34]. Notwithstanding the expression of various stem cell surface markers, cultured hECPs are characterized by relatively restricted adipogenic and osteogenic lineage differentiation capacities, on a biopsy site-specific basis [11]. Regarding cell-surface marker stability, materials from various cell bank lots of the FE002-Cart cell type had been studied in terms of surface marker profile evolution along the in vitro lifespan upon serial banking (i.e., 9, 18, and 24 population doublings), wherein no modifications were observed in said profiles throughout the tested timepoints [34]. Several scaffold or cell-delivery options have been investigated, comprising polyethylene glycols, chitosan, albumin, or hyaluronan for injectable applications, complementing adhesive, chondrogenic, and mitogenic properties [65,66]. Additionally, it is well established that alginate favors both the production and maintenance of extracellular matrix components in vivo, positively impacting structural stability parameters, while resisting vessel infiltration and mineralization [12,64,67,68,69]. Energy dissipation modulation additionally favors optimal chondrogenic expression under dynamic loading and subsequent load bearing, with the upregulation of specific chondrogenic markers (e.g., mRNA of *ACAN*, *COL2A1*, *SOX9*, or *TGFB3*) in hECPs [70,71,72,73]. Bioengineered construct manufacturing specifications and processes, such as cell seeding and preculture periods, external or internal biochemical modulation, and scaffold mechanical stimulation are determinants for obtention of adequate responsiveness and stability, as well as relevant mass transport properties under load bearing [74,75,76,77,78,79,80,81,82,83,84,85,86,87,88].

On a practical clinical side, the hECP-MACI-like combination protocol set forth in the present study comprises the use of off-the-freezer therapeutic cells, extemporaneously seeded to form the combination product. This prompted the verification of therapeutic cell survival and preliminary cytocompatibility with the collagen scaffolds, in view of potential surgical delays before implantation. The confirmed two-hour window of operability available with the seeded constructs provided sufficient flexibility for the operating room staff and further excludes the need for an in-theatre dedicated incubator. Technically, the hECP-seeded membranes were easy to manipulate and apply onto the subchondral bed, without any perceivable loss in mechanical integrity during handling. Surgical fibrin sealant was, thereafter, easily dispersed in the remaining defect cavity for optimal sealing thereof.

With regard to observed outcomes in the caprine pilot safety study, it is necessary to mention that significant rates of delamination in the model may have been attributed to premature post-operative limb loading and movement, which may be drastically reduced in human patients. Sutures may be used to increase fixation, yet these may either damage surrounding tissues or remain for prolonged time periods in the joint, as intra-articular suture resorption rates might be highly inhomogeneous compared to those observed in highly vascularized tissues [54]. This would not preclude the use of an arthroscopic approach in clinical settings, in order to reduce scarring. The activation of fibrin (i.e., sealant) in situ may also have deleterious biological effects, accelerating fibrotic cascades and impairing the proper migration of mesenchymal precursors from the bone marrow. Therefore, the biological burden of using fibrin tissue sealant has to be further investigated, particularly with regard to its potential effects on mesenchymal clot formation and cytotoxicity.

As complete tissue repair (i.e., full resurfacing) within a critical-sized cartilage defect is not expected to be reached within the first three months, choosing to terminate the study at this time-point coincided with our aim to study the early stages of repair. The study of in vivo hECP viability was of great interest from a mechanistic point of view, as anticipated immuno-modulatory activity and anti-inflammatory effects would highly benefit an unstable injury microenvironment, such as that found in full chondral lesions with breaches in subchondral tissue [89,90,91]. Indeed, as secondary arthritis is quite common in goats, the animals showing signs of joint inflammation presented notable sequelae, unrelated to the induced defects. It is interesting to note that the inflammation observed in both control cases did not seem to be linked to secondary erosions. In general, active subchondral reactions with formation of undifferentiated, GAG-depositing mesenchyme are key, as is an overall bone remodeling trend towards bone formation. In cases where subchondral bone is unreactive and mature fibrotic tissue is deposited, the potential for further remodeling is limited and, therefore, undesirable.

### 4.4. Tiered hECP Banking for Safe and Sustainable Cartilage Cell Therapies

The specific bioprocessing methodologies and multi-tiered cell bank establishment applied for the clinical-grade FE002-Cart progenitor cell sources are of high interest from quality assurance and sustainability points of view (Table 2). Indeed, exploiting the inherent capacities of large manufacturing yields facilitates the rapid and tangible industrial transposition to GMP manufacturing, eventually resulting in overall negligible direct costs of allogeneic therapeutic cell manufacture. To this end, specific product release and characterization testing of the cell bank lots in production (i.e., MCBs and WCBs) may integrate assays for cell proliferation, isoenzyme testing for origin validation, DNA fingerprinting, qualification/testing for sterility, specific testing for absence demonstration of mycoplasma, endotoxins, viral contaminants (e.g., adenovirus, B19 parovirus, BPyV, EBV, HuPyV, HPV, HBoV, HAV, HBV, HCV, hCMV, HIV-1, HIV-2, HTLV-1, HTLV-2, HHV-6, HHV-7, HHV-8, KIPyV, orthomyxovirus, paramyxovirus, picornavirus, reovirus, West Nile virus, WUPyV, SV40), quantification of reverse transcriptase activity, or quantitative transmission electron microscopy (TEM) of cell sections for the detection of viruses, virus-like particles, mycoplasma, yeasts, fungi, or bacteria [41]. Safety assessments to be performed on EOPCB materials may comprise in vivo tumorigenicity assays and karyology studies, as performed for the FE002-Cart cell type in this study (Figure 3). Overall, standard industrial technical specifications, testing, and validation strategies are well-adapted for the large-scale manufacture of therapeutic hECPs, due to high robustness, consistency, and stability of the considered biological material sources (Figure 1).

Overall, the most interesting aspect of the Swiss FPC transplantation program is the initial requirement for only one single organ donation following a controlled and medically indicated pregnancy termination, for the parallel establishment of progenitor cell types from different tissues, among which is epiphyseal cartilage. From the same initial organ donation (i.e., FE002, 2009), dermal progenitor cell sources had been established, and the overall framework biobanking model was established and validated using this source in industrialized and clinical settings [30,41]. Specifically, it was projected that GMP manufacture could tangibly be considered for generating over 39 billion individual product doses of therapeutic cells at defined passages within the in vitro lifespan of the cell type (i.e., FE002-SK2 cells), from one controlled organ donation. Such considerations are further uniquely complemented by the fact that allogeneic cellular products obtained in this manner have been safely and successfully used for over two decades in Lausanne for treating burn wounds and severe ulcers, as well as in multiple clinical trials in Europe and Asia [41]. Therefore, building on the translational (i.e., formulated products and hundreds of treated patients) and transpositional (i.e., multiple GMP technology transfers) hindsight gathered for cutaneous regenerative medicine, and given the highly similar behaviors and characteristics of hECPs from the same organ donation (i.e., FE002), the high potential value with regard to sustainability and quality assurance of the FE002-Cart cell type are assured [34]. Furthermore, to complement the preliminary safety data presented herein, additional work of collaborator groups around the same hECP cell source have preliminarily confirmed safety in alternate preclinical models (i.e., nude, NSG, NSG-SGM3, and C57BL/6 murine subcutaneous implantation models), along with potential modulation of chondrogenic activity by physical and chemical cues [12,64,92,94]. Overall, it had been shown in at least three in vivo studies that considered hECPs (i.e., same source from cell banks established in our laboratory) could be implanted in immunocompromised, humanized, and immunocompetent mice without the formation of tumors or rejection of implanted materials.

### 4.5. FE002-Cart hECPs as Safe Cell Sources for Musculoskeletal Tissue Engineering

As shown in the CAM biocompatibility assay and in the caprine safety study, the hECP source of interest was preliminarily confirmed to be safe for therapeutic use, as the materials did not significantly induce chicken embryo death, nor did they cause observable immunologic or tumorigenic effects in the large animal test-subjects. With regard to acceptance of the cells in xenogeneic transplantation, it appeared that the capability of hECPs to engraft in caprine host tissues did not seem to be exclusively linked to inflammatory states. Indeed, one of the relatively stable harvested joints (i.e., ECP6) displayed signs of human cell engraftment onto the synovial membrane. A very different pattern was, however, noted in ECP5, where the joint state was unstable, and the synovial membrane showed signs of widely integrated hECPs (Figure S5). As no clumping patterns of hECPs were observed (i.e., excluding caprine cells and tissues), the positive integration alternative appeared to be favored, rather than ectopic tissue and cell growth. Host tissue engraftment and homing capacities of hECPs may help determine parameters of efficacy and targeting. As no hECPs were detected within defects or subchondral tissues at the three-month time point, their survival and persistence are highly questionable. They may however initially provide the necessary cues to direct or chaperone regeneration early after implantation. Indeed, as the mesenchymal blood clot is expected to form within days of the procedure, the cues delivered during the clotting process, as well as the early cues present during early remodeling, may be key in driving in situ host mesenchymal cell differentiation and in directing tissue building toward a regenerative rather than a repair path. Specifically, hECP implantation did not seem to exacerbate degenerative joint conditions and may well provide necessary protective cues to maintain joint homeostasis during repair. Elucidation of the composition and provenance of newly deposited extra-cellular matrix within the defect sites would indicate if hECPs preferentially work as chaperones of repair rather than builders of new tissue.

The present study further confirmed that the preliminary safety of hECPs manufactured under defined specifications, as described in vitro, may very well extend to in vivo situations. Preliminary safety had already been assessed during sub-cutaneous implantation of bioengineered constructs, yielding FE002-Cart hECPs in rodent and murine models, wherein no immune system activation or tumor formation had been noted [36]. Parallelly, Park et al. have recently shown comparable results with hECPs isolated from a different anatomical site, a different cell culture initiation methodology, and a different preclinical model (i.e., non-human primate) [95]. With respect to the caprine model used for the present study, any efficacy demonstration was mitigated by the rates of delamination and subchondral bone cyst formation, caused by synovial fluid pressure increases, inducing the variability of results both in the CTRL and hECP groups. Securing bioengineering constructs in place is, therefore, crucial to avoid the formation of subchondral bone cysts and delamination. It is important to further optimize the delivery vehicle of hECPs onto cartilage defect sites (e.g., using hydrogels with enhanced mechanical and adhesive properties), in order to optimize therapeutic outcomes in subsequent preclinical and clinical studies. Due to the high versatility and cyto-compatibility of the enzymatically isolated FE002-Cart cell source, the valorization thereof may be undertaken in many cell-based cartilage repair approaches. Additionally, as hECPs are expected to act early-on after implantation by interacting with surrounding tissues and orchestrate regeneration, the biodegradation profile of the carrier matrix may be tuned to be relatively faster than that of collagen membranes. The further optimization of the cell delivery vehicle and implantation procedure will allow for improved design and evaluation of long-term hECP efficacy in attempting to achieve functional cartilage regeneration.

### 4.6. Study Limitations

The scope of the present study encompassed the experimental validation of industrial-scale multi-tiered cell banking workflow for hECPs, the in ovo evaluation of hECP embryotoxicity on a standardized CAM model, and finally the in vivo preliminary preclinical evaluation of hECP safety in a caprine model. Taken individually and from an efficacy point of view, the in vivo caprine study did not show statistically significant improvement in the cell therapy group. However, these results are most interesting from the safety standpoint, as no incurred adverse effects or rejections were noted in the test and control groups. Furthermore, in the setting of a preliminary safety evaluation, the termination of the study at three months did not allow us to satisfactorily evaluate an efficacy outcome, which shall be specific to further long-term preclinical and/or clinical investigations. Therefore, the scope and primary objective of the present study with respect to the caprine experiments (i.e., preliminary safety) need to be placed in this context for appropriate analysis. Evaluation of the overall outcomes of the proposed hECP-based cell therapy will only be possible once efficacy preclinical studies or clinical studies have been performed.

Taken again from an efficacy endpoint, the absence of statistical significance in inter-group differences in long-term studies may be considered as insufficient to demonstrate the superiority of one cell type over the controls. However, and most importantly, in the present setting, the absence of detectable differences at an early timepoint may be considered as an advantage of prime importance, demonstrating the absence of early adverse effect eliciting. Acceptable management options for large tissue defects are still to be found, and studies such as those presented herein preliminarily allow for further consideration of alternative cell sources (i.e., or at the very least, allow to not exclude them for safety reasons) and, therefore, contribute to further pursuing investigations for optimal therapy elaboration.

## 5. Conclusions

This study describes the industrial transposition of hECP multi-tiered cell banking for therapeutic product development, following a single fetal organ donation, for optimized cartilage defect repair or regeneration. Therein, particular focus was set on preclinical hECP safety by considering a standardized therapeutic FPC source (i.e., FE002-Cart cell type). An optimized cell banking workflow was proposed, potentially generating millions of safe and sustainable therapeutic products after transposition to the GMP manufacturing of product cell lots for cATMPs. Furthermore, hECP clinical cell doses were characterized as non-embryotoxic and non-angiotoxic in a standardized chorioallantoic membrane model, with an absence of significantly induced mortality in exposed embryos. Lastly, a hECP-MACI combination was applied in a three-month GLP pilot safety evaluation in a caprine model of full-thickness articular cartilage defect. The safety of hECP transplantation was highlighted in xenogeneic settings, along with confirmed needs for optimal cell delivery vehicles and implantation techniques favoring effective cartilage tissue repair or regeneration [96]. Overall, hECPs were confirmed as safe, stable, and sustainable therapeutic material leads for the development of regenerative therapies and related cell-based products in robust industrial settings. Preliminary safety data favor the continued investigation of hECP therapeutic potential in further preclinical and clinical settings. The integrated conclusions of the present work further strengthen the rationale set forth under our Swiss fetal progenitor cell transplantation program, enabling continued tangible advances in musculoskeletal medical development toward the betterment of global patient health.

## Figures and Tables

**Table 1:** Clinically available options for chondral injury treatment.

Treatment	Description	Benefits	Limitations
Non-surgical methods	Analgesics, weight loss, physical re-education, physical therapy, complementary medicine (e.g., acupuncture)	No surgery necessary Relatively inexpensive	Palliative treatment options Chronic analgesic use potential
Arthroscopic chondroplasty	Arthroscopic resection of detached cartilage fragments to prevent further joint irritation and damage	Rapid, minimally invasive procedureImmediate weight-bearing possible	Palliative treatment Benefits not proven Brief pain relief
MF ^1^	Arthroscopic procedure to create small lesions in the osteochondral interface to direct osteoprogenitor cells into damaged cartilage tissue	No graft required for defects < 2 cm^2^ Rapid recovery	Fibrocartilage formation potential Variable functional outcomes Rapid deterioration
Mosaicoplasty	Osteochondral autografts harvested from patient non-loaded zones of the joint	Optimal for small lesions (1–4 cm^2^) Pain relief	Sub-optimal surrounding tissue graft adherence Graft-site morbidity
ACI ^2^	Autologous cultured and expanded chondrocytes implanted in the lesion under a tissue flap	Hyaline cartilage formation potential Extensive lesion treatment (<10 cm^2^)	Graft delamination Periosteal hypertrophy Qualitative variability of regenerated tissue Onerous protocol
MACI ^3^	Autologous cultured and expanded chondrocytes seeded onto 3D scaffolds and implanted into defect	Treatment of large lesions Hyaline cartilage formation potential Limited periosteal hypertrophy	Graft delamination Limited integration Qualitative variability of regenerated tissue Onerous protocol
Joint arthroplasty	Replacement of arthritic joint with an artificial implant	Pain relief Functional resurgence potential	Infectious risk Functional outcome variability Implant wear or loosening

^1^ Microfracture, ^2^ Autologous chondrocyte implantation, ^3^ Matrix-assisted autologous chondrocyte transplantation.

**Table 2:** FE002-Cart cell type characterization overview. Data succinctly comprise findings of our research group and those of collaborator groups working on the same primary cell source of interest.

Cell Type Characteristic	Data, Findings ^1^	References
Identity	“FE002-Cart”, primary diploid cell type, from ulnar epiphysis Fibroblast-like stable cellular morphology in 2D in vitro culture Defined surface marker profile (i.e., CD14^−^, CD34^−^, CD45^−^, HLA-DP/DQ/DR^−^, CD26^+^, CD44^+^, CD73^+^, CD90^+^, CD105^+^, CD166^+^, HLA-ABC^+^) ^2^	[34]
Stability	Establishment and testing of EOPCB ^3^ at Passage 12 ^1^ Normal 46X,Y karyotype stable up to Passage 12 ^1^ Lifespan > 35 PDs ^4^ with stable in vitro expansion kinetics Resistance to adipogenic and osteogenic induction, cryogenic shock	[34]
Cytocompatibility	Cytocompatible with various hydrogel formulations (e.g., alginate and hyaluronan-based polymer gels) High shear stress resistance for seeding in bioengineered constructs Clinically relevant scaffold stiffness generation under stimulation	[64,92,93]
Safety	Non-toxic and no angiogenesis perturbation in CAM ^5^ model ^1^ Non-immunogenic and non-tumorigenic in murine and rat models of cartilage defect or subcutaneous implantation Non-immunogenic, non-tumorigenic, and no slowing of cartilage defect healing in caprine model ^1^	[36,92]
Functionality	Spontaneous chondrogenic activity in 3D micropellets Potent and stable production of ECM ^6^ (i.e., GAGs ^7^, aggrecan, types I and II collagen) Important functional responsiveness to mechanostimulation in dynamic scaffold culture conditions Highly responsive chondrogenic potential under biochemical stimulation (e.g., alginate, TGF-β1) Highly responsive chondrogenic potential in specific formulations (e.g., therapeutic cell microencapsulation)	[12,34,36,70,92,94]

^1^ Original data, ^2^ cluster of differentiation, ^3^ end of production cell bank, ^4^ population doubling, ^5^ chorioallantoic membrane, ^6^ extracellular matrix, ^7^ glycosaminoglycan.

## Supplementary Materials

The supplementary figures, methods, data, and tables are available online at https://www.mdpi.com/2218-273X/11/2/250/s1.

## Appendix A

The various scoring methodologies for evaluation of caprine cartilage repair are presented hereafter.

**Table A1:** Macroscopic scoring table for evaluation of cartilage state and repair in four zones (i.e., Zone 0 to Zone 3).

**Description—Defect Filling Score Scale (Zones 0 ^1^ and 1 ^2^)**	**Grade**
Totally filled	0
Peripheral and central filling. No MF drill holes visible	1
Peripheral filling. Very thin transparent filling. Visible drill holes	2
Peripheral filling and/or blood-tinged	3
No filling	4
**Description—Modified Outerbridge Score Scale (Zones 2 ^3^ and 3 ^4^)**	**Grade**
Normal cartilage appearance (hyaline, flat, smooth on the surface)	0
Cobblestone appearance, soft, swollen cartilage	1
Fissures visible in cartilage	2

^1^ Defect center, ^2^ defect rim, ^3^ adjacent margin, ^4^ distal margin.

**Table A2:** Histological scoring scale modified from the original O’Driscoll score scale for cartilage defect repair evaluation (i.e., Zones 0 and 1). The score was used to evaluate the defect filling according to cellular morphology, Safranin-O staining, surface coverage, thickness, bonding of new tissue to adjacent cartilage, hypocellularity of hyaline-like tissue, and chondrocyte clustering.

Description—Cellular Morphology	Grade
Hyaline articular cartilage	0
Incompletely differentiated mesenchyme	1
Fibrous tissue	2
Bone	3
**Description—Safranin-O Staining of the Matrix**	**Grade**
Normal	0
Moderate	1
Slight	2
None	3
**Description—Surface coverage**	**Grade**
Smooth and intact (100% coverage)	0
75–50% coverage	1
50–25% coverage	2
Severe disruption (<25% coverage)	3
**Description—Thickness**	**Grade**
75–100% of normal adjacent cartilage	0
50–75% of normal adjacent cartilage	1
25–50% of normal adjacent cartilage	2
0–25% of normal adjacent cartilage	3
**Description—Bonding of new tissue to adjacent cartilage (Zone 1)**	**Grade**
Bonded at both ends	0
Bonded at one end, or partially at both ends	1
Not bonded	2
**Description—Hypocellularity of hyaline-like tissue**	**Grade**
Non-hyaline	N/A
Normal cellularity	0
Slight hypocellularity or slight hypercellularity	1
Moderate hypocellularity	2
Severe hypocellularity	3
**Description—Chondrocyte clustering**	**Grade**
Non-hyaline tissue and cells	N/A
No chondrocyte clusters	0
<25% of the cells	1
25–100% of the cells	2

**Table A3:** Scoring according to the Little score scale for osteochondral tissue (i.e., Zones 2 and 3). The score was used to evaluate the chondral and subchondral tissue surrounding the defect according to structure, cellularity, cell cloning, territorial, and interterritorial Toluidine Blue staining, as well as the tidemark, calcified cartilage, and subchondral bone tissue.

Description—Structure	Grade
Normal	0
Slight surface irregularities	1
Moderate surface irregularities	2
Severe surface irregularities	3
Clefts into transitional zone (1/3 depth)	4
Clefts into radial zone (2/3 depth)	5
Clefts into calcified zone (full depth)	6
Fibrillation and/or loss to transitional zone (1/3 depth)	7
Fibrillation and/or loss to radial zone (2/3 depth)	8
Fibrillation and/or loss to calcified zone (full depth)	9
Fibrillation and/or loss to subchondral bone	10
**Description—Cellularity**	**Grade**
Normal	0
Increase or slight decrease	1
Moderate decrease	2
Severe decrease	3
No cells	4
**Description—Cell cloning**	**Grade**
Normal	0
Several doublets	1
Many doublets	2
Doublets and triplets	3
Multiple cell nests	4
**Description—Territorial Toluidine Blue**	**Grade**
Normal	0
Increase or slight decrease in staining	1
Moderate decrease	2
Severe decrease	3
No staining	4
**Description—Interterritorial Toluidine Blue**	**Grade**
Normal	0
Loss of staining in tangential zone (1/3 depth)	1
Loss of staining in transitional zone (2/3 depth)	2
Loss of staining in radial zone (full depth)	3
No staining	4
**Description—Tidemark/Calcified cartilage/Subchondral bone**	**Grade**
Intact subchondral bone plate and single tidemark	0
Intact subchondral bone plate and multilayered tidemark	1
Blood vessels through subchondral bone to calcified cartilage	2
Tidemark crossed by blood vessels	3

**Table A4:** Krenn synovitis scoring scale. Indications of thickening synovial lining cell layers, increased resident cell density, and inflammatory infiltrates were evaluated by three investigators. A consensus score was reported for each parameter.

Description—Enlargement of Synovial Lining Cell Layer	Grade
Lining cells form 1 layer	0
Lining cells form 2–3 layers	1
Lining cells form 4–5 layers, with few multinucleated cells	2
Lining cells form more than 5 layers, with ulceration, multinucleated cells	3
**Description—Density of Resident Cells**	**Grade**
Synovial stroma shows normal cellularity	0
Cellularity slightly increased	1
Cellularity moderately increased, multinuclear cells might occur	2
Cellularity greatly increased, multinucleated giant cells, pannus, rheumatoid granulomas	3
**Description—Inflammatory Infiltrate**	**Grade**
No inflammatory infiltrate	0
Few, mostly perivascular situated lymphocytes or plasma cells	1
Numerous lymphocytes or plasma cells, sometimes forming follicle-like aggregates	2
Dense band-like inflammatory infiltrate or numerous large follicle-like aggregates	3
**Description—Synovitis**	**Total**
No synovitis	0–1
Low-grade synovitis	2–4
High-grade synovitis	5–9

**Table A5:** Modified Rooney synovitis scoring scale. Indications of synoviocyte hyperplasia, fibrosis, proliferating blood vessels, perivascular and diffuse lymphocyte infiltrates, and focal lymphocyte aggregates were evaluated by three investigators. A consensus score was reported for each parameter.

Synoviocyte Hyperplasia: Predominant Cell Depth of Synovial Lining Layer	
Description	Grade
1–2	0
3–4	1
5–6	2
7–8	3
9–10	4
>10	5
**Fibrosis: Percentage of Fibrosis in Subsynovial Layer**	
**Description**	**Grade**
<10	0
10–30	1
30–50	2
50–70	3
70–90	4
>90	5
**Proliferating blood vessels: Number of Vessels Per High Power Field ^1^**	
**Description**	**Grade**
0–3	0
4–8	1
9–13	2
14–18	3
18–22	4
>22	5
**Perivascular Infiltrates of Lymphocytes: Percentage of Vessels Per High Power Field**	
**Description**	**Grade**
<5	0
5–25	1
25–50	2
50–75	3
100	4
**Focal lymphocyte Aggregates: Number of Cells in Diameter**	
**Description**	**Grade**
10	0
20	1
30	2
40	3
50	4
≥55	5
**Diffuse Infiltrates of Lymphocytes: Percentage of Diffuse Lymphocytes Per High Power Field**	
**Description**	**Grade**
0	0
5–20	1
20–40	2
40–60	3
60–80	4
100	5

^1^ HPF = 20.

**Table A6:** MRI score scale for post-harvest evaluation and scoring of subchondral bone sclerosis and bone marrow edema in caprine stifles.

Description	Grade
No finding	0
Mild	1
Moderate	2
Severe	3
Profound	4

## References

[1] Vacanti J.P., Langer R. (1999). Tissue engineering: The design and fabrication of living replacement devices for surgical reconstruction and transplantation. Lancet.

[2] Marks P., Gottlieb S. (2018). Balancing safety and innovation for cell-based regenerative medicine. N. Engl. J. Med..

[3] Vrahas M.S., Mithoefer K., Joseph D. (2004). The long-term effects of articular impaction. Clin. Orthop. Relat. Res..

[4] Flanigan D.C., Harris J.D., Trinh T.Q., Siston R.A., Brophy R.H. (2010). Prevalence of chondral defects in athletes’ knees: A systematic review. Med. Sci. Sports Exerc..

[5] Makris E.A., Gomoll A.H., Malizos K.N., Hu J.C., Athanasiou K.A. (2015). Repair and tissue engineering techniques for articular cartilage. Nat. Rev. Rheumatol..

[6] Brittberg M., Lindahl A., Nilsson A., Ohlsson C., Isaksson O., Peterson L. (1994). Treatment of deep cartilage defects in the knee with autologous chondrocyte transplantation. N. Engl. J. Med..

[7] Horas U., Pelinkovic D., Herr G., Aigner T., Schnettler R. (2003). Autologous chondrocyte implantation and osteochondral cylinder transplantation in cartilage repair of the knee joint. A prospective, comparative trial. J. Bone Joint Surg. Am..

[8] Lu Y., Dhanaraj S., Wang Z., Bradley D.M., Bowman S.M., Cole B.J., Binette F. (2006). Minced cartilage without cell culture serves as an effective intraoperative cell source for cartilage repair. J. Orthop. Res..

[9] Katopodi T., Tew S.R., Clegg P.D., Hardingham T.E. (2009). The influence of donor and hypoxic conditions on the assembly of cartilage matrix by osteoarthritic human articular chondrocytes on Hyalograft^®^ matrices. Biomaterials.

[10] Vinardell T., Sheehy E.J., Buckley C.T., Kelly D.J. (2012). A comparison of the functionality and in vivo phenotypic stability of cartilaginous tissues engineered from different stem cell sources. Tissue Eng. Part A.

[11] Quintin A., Schizas C., Scaletta C., Jaccoud S., Applegate L.A., Pioletti D.P. (2010). Plasticity of fetal cartilaginous cells. Cell Transplant..

[12] Studer D., Cavalli E., Formica F.A., Kuhn G.A., Salzmann G., Mumme M., Steinwachs M.R., Laurent-Applegate L.A., Maniura-Weber K., Zenobi-Wong M. (2017). Human chondroprogenitors in alginate-collagen hybrid scaffolds produce stable cartilage in vivo. J. Tissue Eng. Regen. Med..

[13] Park J., Gelse K., Frank S., von der Mark K., Aigner T., Schneider H. (2006). Transgene-activated mesenchymal cells for articular cartilage repair: A comparison of primary bone marrow-, perichondrium/periosteum- and fat-derived cells. J. Gene Med..

[14] Wakitani S., Nawata M., Tensho K., Okabe T., Machida H., Ohgushi H. (2007). Repair of articular cartilage defects in the patello-femoral joint with autologous bone marrow mesenchymal cell transplantation: Three case reports involving nine defects in five knees. J. Tissue Eng. Regen. Med..

[15] Hwang N.S., Varghese S., Elisseeff J. (2008). Derivation of chondrogenically-committed cells from human embryonic cells for cartilage tissue regeneration. PLoS ONE.

[16] Prockop D.J. (2009). Repair of tissues by adult stem/progenitor cells (MSCs): Controversies, myths, and changing paradigms. Mol. Ther..

[17] Saw K.Y., Hussin P., Loke S.C., Azam M., Chen H.C., Tay Y.G., Low S., Wallin K.L., Ragavanaidu K. (2009). Articular cartilage regeneration with autologous marrow aspirate and hyaluronic acid: An experimental study in a goat model. Arthroscopy.

[18] Fortier L.A., Potter H.G., Rickey E.J., Schnabel L.V., Foo L.F., Chong L.R., Stokol T., Cheetham J., Nixon A.J. (2010). Concentrated bone marrow aspirate improves full-thickness cartilage repair compared with microfracture in the equine model. J. Bone Joint Surg. Am..

[19] Toh W.S., Lee E.H., Guo X.M., Chan J.K., Yeow C.H., Choo A.B., Cao T. (2010). Cartilage repair using hyaluronan hydrogel-encapsulated human embryonic stem cell-derived chondrogenic cells. Biomaterials.

[20] Zscharnack M., Hepp P., Richter R., Aigner T., Schulz R., Somerson J., Josten C., Bader A., Marquass B. (2010). Repair of chronic osteochondral defects using predifferentiated mesenchymal stem cells in an ovine model. Am. J. Sports Med..

[21] Gupta P.K., Das A.K., Chullikana A., Majumdar A.S. (2012). Mesenchymal stem cells for cartilage repair in osteoarthritis. Stem Cell Res. Ther..

[22] Torrero J.I., Aroles F., Ferrer D. (2012). Treatment of knee chondropathy with platelet rich plasma. Preliminary results at 6 months of follow-up with only one injection. J. Biol. Regul. Homeost. Agents.

[23] Bekkers J.E., Creemers L.B., Tsuchida A.I., van Rijen M.H., Custers R.J., Dhert W.J., Saris D.B. (2013). One-stage focal cartilage defect treatment with bone marrow mononuclear cells and chondrocytes leads to better macroscopic cartilage regeneration compared to microfracture in goats. Osteoarthr. Cartil..

[24] Lee G.W., Son J.H., Kim J.D., Jung G.H. (2013). Is platelet-rich plasma able to enhance the results of arthroscopic microfracture in early osteoarthritis and cartilage lesion over 40 years of age?. Eur. J. Orthop. Surg. Traumatol..

[25] Orozco L., Munar A., Soler R., Alberca M., Soler F., Huguet M., Sentis J., Sanchez A., Garcia-Sancho J. (2013). Treatment of knee osteoarthritis with autologous mesenchymal stem cells: A pilot study. Transplantation.

[26] Pelttari K., Pippenger B., Mumme M., Feliciano S., Scotti C., Mainil-Varlet P., Procino A., von Rechengerg B., Schwamborn T., Jakob M. (2014). Adult human neural crest-derived cells for articular cartilage repair. Sci. Transl. Med..

[27] Pleumeekers M.M., Nimeskern L., Koevoet W.L., Kops N., Poublon R.M., Stok K.S., van Osch G.J. (2014). The in vitro and in vivo capacity of culture-expanded human cells from several sources encapsulated in alginate to form cartilage. Eur. Cells Mater..

[28] Steinwachs M.R., Waibl B., Wopperer S., Mumme M. (2014). Matrix-associated chondroplasty: A novel platelet-rich plasma and concentrated nucleated bone marrow cell-enhanced cartilage restoration technique. Arthrosc. Tech..

[29] De Buys Roessingh A.S., Hohlfeld J., Scaletta C., Hirt-Burri N., Gerber S., Hohlfeld P., Gebbers J.O., Applegate L.A. (2006). Development, characterization, and use of a fetal skin cell bank for tissue engineering in wound healing. Cell Transplant..

[30] Applegate L.A., Scaletta C., Hirt-Burri N., Raffoul W., Pioletti D. (2009). Whole-cell bioprocessing of human fetal cells for tissue engineering of skin. Skin Pharmacol. Physiol..

[31] Almqvist K.F., Dhollander A.A., Verdonk P.C., Forsyth R., Verdonk R., Verbruggen G. (2009). Treatment of cartilage defects in the knee using alginate beads containing human mature allogenic chondrocytes. Am. J. Sports Med..

[32] Adkisson H.D., Milliman C., Zhang X., Mauch K., Maziraz R.T., Streeter P.R. (2010). Immune evasion by neocartilage-derived chondrocytes: Implications for biologic repair of joint articular cartilage. Stem Cell Res..

[33] Acosta F.L., Metz L., Adkisson H.D., Liu J., Carruthers-Liebenberg E., Milliman C., Maloney M., Lotz J.C. (2011). Porcine intervertebral disc repair using allogeneic juvenile articular chondrocytes or mesenchymal stem cells. Tissue Eng. Part A.

[34] Darwiche S.E., Scaletta C., Raffoul W., Pioletti D.P., and Applegate L.A. (2012). Epiphyseal chondroprogenitors provide a stable cell source for cartilage cell therapy. Cell Med..

[35] Dhollander A.A., Verdonk P.C., Lambrecht S., Verdonk R., Elewaut D., Verbruggen G., Almqvist K.F. (2012). Midterm results of the treatment of cartilage defects in the knee using alginate beads containing human mature allogenic chondrocytes. Am. J. Sports Med..

[36] Cavalli E., Fisch P., Formica F.A., Gareus R., Linder T., Applegate L.A., Zenobi-Wong M. (2018). A comparative study of cartilage engineered constructs in immunocompromised, humanized and immunocompetent mice. J. Immunol. Regen. Med..

[37] Zimmermann P., Boeuf S., Dickhut A., Boehmer S., Olek S., Richter W. (2008). Correlation of COL10A1 induction during chondrogenesis of mesenchymal stem cells with demethylation of two CpG sites in the COL10A1 promoter. Arthritis Rheum..

[38] Tompkins M., Adkisson H.D., Bonner K.F. (2013). De novo NT allograft. Operat. Techn. Sports Med..

[39] Doyle A., Griffiths J.B. (1998). Cell and Tissue Culture: Laboratory Procedures in Biotechnology.

[40] Quintin A., Hirt-Burri N., Scaletta C., Schizas C., Pioletti D.P., Applegate L.A. (2007). Consistency and safety of cell banks for research and clinical use: Preliminary analysis of fetal skin banks. Cell Transplant..

[41] Laurent A., Lin P., Scaletta C., Hirt-Burri N., Michetti M., de Buys Roessingh A.S., Raffoul W., She B.R., Applegate L.A. (2020). Bringing Safe and Standardized Cell Therapies to Industrialized Processing for Burns and Wounds. Front Bioeng. Biotechnol..

[42] Shah M., Foreman D.M., Ferguson M.W.J. (1992). Control of scarring in adult wounds by neutralising antibody to transforming growth factor β. Lancet.

[43] Cass D.L., Meuli M., Adzick N.S. (1997). Scar wars: Implications of fetal wound healing for the pediatric burn patient. Pediatr. Surg. Int..

[44] Ribatti D. (2016). The chick embryo chorioallantoic membrane (CAM). A multifaceted experimental model. Mech. Dev..

[45] Outerbridge R.E. (1961). The etiology of chondromalacia patellae. J. Bone Joint Surg. Br..

[46] O’Driscoll S.W., Marx R.G., Beaton D.E., Miura Y., Gallay S.H., Fitzsimmons J.S. (2001). Validation of a simple histological-histochemical cartilage scoring system. Tissue Eng..

[47] Little C., Smith S., Ghosh P., Bellenger C. (1997). Histomorphological and immunohistochemical evaluation of joint changes in a model of osteoarthritis induced by lateral meniscectomy in sheep. J. Rheumatol..

[48] Krenn V., Morawietz L., Burmester G.R., Kinne R.W., Mueller-Ladner U., Muller B., Haupl T. (2006). Synovitis score: Discrimination between chronic low-grade and high-grade synovitis. Histopathology.

[49] Yamanaka H., Goto K., Miyamoto K. (2010). Scoring evaluation for histopathological features of synovium in patients with rheumatoid arthritis during anti-tumor necrosis factor therapy. Rheumatol. Int..

[50] Ahmed T.A., Hincke M.T. (2010). Strategies for articular cartilage lesion repair and functional restoration. Tissue Eng. Part B Rev..

[51] Bedi A., Feeley B.T., Williams R.J. (2010). Management of articular cartilage defects of the knee. J. Bone Joint Surg. Am..

[52] Brittberg M. (2010). Cell carriers as the next generation of cell therapy for cartilage repair: A review of the matrix-induced autologous chondrocyte implantation procedure. Am. J. Sports Med..

[53] Teo A.Q.A., Wong K.L., Shen L., Lim J.Y., Wei S.T., Lee H., Hui J.H.P. (2019). Equivalent 10-year outcomes after implantation of autologous bone marrow-derived mesenchymal stem cells versus autologous chrondrocyte implantation for chondral defects of the knee. Am. J. Sports Med..

[54] Benthien J.P., Behrens P. (2011). The treatment of chondral and osteochondral defects of the knee with autologous matrix-induced chondrogenesis (AMIC): Method description and recent developments. Knee Surg. Sports Traumatol. Arthrosc..

[55] Mithoefer K., McAdams T., Williams R.J., Kreuz P.C., Mandelbaum B.R. (2009). Clinical efficacy of the microfracture technique for articular cartilage repair in the knee: An evidence-based systematic analysis. Am. J. Sports Med..

[56] Gille J., Behrens P., Volpi P., de Girolamo L., Reiss E., Zoch W., Anders S. (2013). Outcome of Autologous Matrix Induced Chondrogenesis (AMIC) in cartilage knee surgery: Data of the AMIC Registry. Arch. Orthop. Trauma Surg..

[57] Piotet L.-M. (2018). Third Generation of Cellular Therapies for Chondral Defects: A Bibliographic Search. Master’s Thesis.

[58] Minas T., Peterson L. (2012). Autologous chondrocyte transplantation. Oper. Tech. Sports Med..

[59] Kon E., Roffi A., Filardo G., Tesei G., Marcacci M. (2015). Scaffold-based cartilage treatments: With or without cells? A systematic review of preclinical and clinical evidence. Arthroscopy.

[60] Dhollander A.A., De Neve F., Almqvist K.F., Verdonk R., Lambrecht S., Elewaut D., Verbruggen G., Verdonk P.C. (2011). Autologous matrix-induced chondrogenesis combined with platelet-rich plasma gel: Technical description and a five pilot patients report. Knee Surg. Sports Traumatol. Arthrosc..

[61] Mumme M., Barbero A., Miot S., Wixmerten A., Feliciano S., Wolf F., Asnaghi A.M., Baumhoer D., Bieri O., Kretzcchmar M. (2016). Nasal chondrocyte-based engineered autologous cartilage tissue for repair of articular cartilage defects: An observational first-in-human trial. Lancet.

[62] Noh M.J., Copeland R.O., Yi Y., Choi K.-B., Meschter C., Hwang S., Lim C.-L., Yip V., Hyun J.-P., Lee H.-Y. (2010). Pre-clinical studies of retrovirally transduced human chondrocytes expressing transforming growth factor-beta.1 (TG-C). Cytotherapy.

[63] Ha C.-W., Noh M.J., Choi K.B., Lee K.H. (2012). Initial phase I safety of retrovirally transduced human chondrocytes expressing transforming growth factor-beta-1 in degenerative arthritis patients. Cytotherapy.

[64] Broguiere N., Cavalli E., Salzmann G.M., Applegate L.A., Zenobi-Wong M. (2016). Factor XIII cross-linked hyaluronan hydrogels for cartilage tissue engineering. ACS Biomater. Sci. Eng..

[65] Madeira C., Santhagunam A., Salgueiro J.B., Cabral J.M. (2015). Advanced cell therapies for articular cartilage regeneration. Trends Biotechnol..

[66] Mardones R., Jofré C.M., Minguell J.J. (2015). Cell therapy and tissue engineering approaches for cartilage repair and/or regeneration. Int. J. Stem Cells.

[67] Häuselmann H.J., Fernandes R.J., Mok S.S., Schmid T.M., Block J.A., Aydelotte M.B., Kuettner K.E., Thonar E.J. (1994). Phenotypic stability of bovine articular chondrocytes after long-term culture in alginate beads. J. Cell Sci..

[68] Mhanna R., Kashyap A., Palazzolo G., Vallmajo-Martin Q., Becher J., Möller S., Schnabelrauch M., Zenobi-Wong M. (2014). Chondrocyte culture in three-dimensional alginate sulfate hydrogels promotes proliferation while maintaining expression of chondrogenic markers. Tissue Eng. Part A.

[69] Mellor L.F., Baker T.L., Brown R.J., Catlin L.W., Oxford J.T. (2014). Optimal 3D culture of primary articular chondrocytes for use in the rotating wall vessel bioreactor. Aviat. Space Environ. Med..

[70] Abdel-Sayed P., Darwiche S.E., Kettenberger U., Pioletti D.P. (2014). The role of energy dissipation of polymeric scaffolds in the mechanobiological modulation of chondrogenic expression. Biomaterials.

[71] Hunter C.J., Mouw J.K., Levenston M.E. (2004). Dynamic compression of chondrocyte-seeded fibrin gels: Effects on matrix accumulation and mechanical stiffness. Osteoarthr. Cartil..

[72] Mauck R.L., Byers B.A., Yuan X., Tuan R.S. (2007). Regulation of cartilaginous ECM gene transcription by chondrocytes and MSCs in 3D culture in response to dynamic loading. Biomech. Model Mechanobiol..

[73] Thorpe S.D., Buckley C.T., Vinardell T., O’Brien F.J., Campbell V.A., Kelly D.J. (2008). Dynamic compression can inhibit chondrogenesis of mesenchymal stem cells. Biochem. Biophys. Res. Commun..

[74] Wendt D., Marsano A., Jakob M., Heberer M., Martin I. (2003). Oscillating perfusion of cell suspensions through three-dimensional scaffolds enhances cell seeding efficiency and uniformity. Biotechnol. Bioeng..

[75] Melchels F.P., Barradas A.M., van Blitterswijk C.A., de Boer J., Feijen J., Grijpma D.W. (2010). Effects of the architecture of tissue engineering scaffolds on cell seeding and culturing. Acta Biomater..

[76] Burg K.J., Holder W.D., Culberson C.R., Beiler R.J., Greene K.G., Loebsack A.B., Roland W.D., Eiselt P., Mooney D.J., Halberstadt C.R. (2000). Comparative study of seeding methods for three-dimensional polymeric scaffolds. J. Biomed. Mater. Res..

[77] Alvarez-Barreto J.F., Linehan S.M., Shambaugh R.L., Sikavitsas V.I. (2007). Flow perfusion improves seeding of tissue engineering scaffolds with different architectures. Ann. Biomed. Eng..

[78] Roh J.D., Nelson G.N., Udelsman B.V., Brennan M.P., Lockhart B., Fong P.M., Lopez-Soler R.I., Salzman W.M., Breuer C.K. (2007). Centrifugal seeding increases seeding efficiency and cellular distribution of bone marrow stromal cells in porous biodegradable scaffolds. Tissue Eng..

[79] Thevenot P., Nair A., Dey J., Yang J., Tang L. (2008). Method to analyze three-dimensional cell distribution and infiltration in degradable scaffolds. Tissue Eng. Part C Methods.

[80] Moretti M., Wendt D., Dickinson S.C., Sims T.J., Hollander A.P., Kelly D.J., Prendergast P.J., Heberer M., Martin I. (2005). Effects of *in vitro* preculture on in vivo development of human engineered cartilage in an ectopic model. Tissue Eng..

[81] Roche S., Ronzière M.C., Herbage D., Freyria A.M. (2001). Native and DPPA cross-linked collagen sponges seeded with fetal bovine epiphyseal chondrocytes used for cartilage tissue engineering. Biomaterials.

[82] Hasegawa T., Miwa M., Sakai Y., Niikura T., Lee S.Y., Oe K., Iwakura T., Kurosaka M., Komori T. (2010). Efficient cell-seeding into scaffolds improves bone formation. J. Dent. Res..

[83] Erickson I.E., Kestle S.R., Zellars K.H., Farrell M.J., Kim M., Burdick J.A., Mauck R.L. (2012). High mesenchymal stem cell seeding densities in hyaluronic acid hydrogels produce engineered cartilage with native tissue properties. Acta Biomater..

[84] Hollister S.J. (2005). Porous scaffold design for tissue engineering. Nat. Mater..

[85] Kemppainen J.M., Hollister S.J. (2010). Differential effects of designed scaffold permeability on chondrogenesis by chondrocytes and bone marrow stromal cells. Biomaterials.

[86] Huang A.H., Farrell M.J., Kim M., Mauck R.L. (2010). Long-term dynamic loading improves the mechanical properties of chondrogenic mesenchymal stem cell-laden hydrogel. Eur. Cells Mater..

[87] Campbell J.J., Lee D.A., Bader D.L. (2006). Dynamic compressive strain influences chondrogenic gene expression in human mesenchymal stem cells. Biorheology.

[88] Terraciano V., Hwang N., Moroni L., Park H.B., Zhang Z., Mizrahi J., Seliktar D., Elisseeff J. (2007). Differential response of adult and embryonic mesenchymal progenitor cells to mechanical compression in hydrogels. Stem Cells.

[89] Montjovent M.O., Bocelli-Tyndall C., Scaletta C., Scherberich A., Mark S., Martin I., Applegate L.A., Pioletti D.P. (2009). *In vitro* characterization of immune-related properties of human fetal bone cells for potential tissue engineering applications. Tissue Eng. Part A.

[90] Ramelet A.A., Hirt-Burri N., Raffoul W., Scaletta C., Pioletti D.P., Offord E., Mansourian R., Applegate L.A. (2009). Chronic wound healing by fetal cell therapy may be explained by differential gene profiling observed in fetal versus old skin cells. Exp. Gerontol..

[91] Pritchard S., Bianchi D.W. (2012). Fetal cell microchimerism in the maternal heart: Baby gives back. Circ. Res..

[92] Li F., Levinson C., Truong V.X., Laurent-Applegate L.A., Maniura-Weber K., Thissen H. (2020). Microencapsulation improves chondrogenesis *in vitro* and cartilaginous matrix stability in vivo compared to bulk encapsulation. Biomater. Sci..

[93] Nasrollahzadeh N., Applegate L.A., Pioletti D.P. (2017). Development of an effective cell seeding technique: Simulation, implementation, and analysis of contributing factors. Tissue Eng. Part C Methods.

[94] Levinson C., Lee M., Applegate L.A., Zenobi-Wong M. (2019). An injectable heparin-conjugated hyaluronan scaffold for local delivery of transforming growth factor β1 promotes successful chondrogenesis. Acta Biomater..

[95] Park D.Y., Min B.H., Park S.R., Oh H.J., Truong M.D., Kim M., Choi J.Y., Park I.S., Choi B.H. (2020). Engineered cartilage utilizing fetal cartilage-derived progenitor cells for cartilage repair. Sci. Rep..

[96] Cherian J.J., Parvizi J., Bramlet D., Lee K.H., Romness D.W., Mont M.A. (2015). Preliminary results of a phase II randomized study to determine the efficacy and safety of genetically engineered allogeneic human chondrocytes expressing TGF-B1 in patients with grade 3 chronic degenerative joint disease of the knee. Osteoarthr. Cart..

