# Supplementary material for: Development of Standardized Fetal Progenitor Cell Therapy for Cartilage Regenerative Medicine: Industrial Transposition and Preliminary Safety in Xenogeneic Transplantation
Source: Biomolecules. 2021 Feb 9;11(2):250. doi: 10.3390/biom11020250 (PMC7916236; doi:10.3390/biom11020250)
Supplement: Supplementary file 1 [file biomolecules-11-00250-s001.pdf]

Supplementary Figures

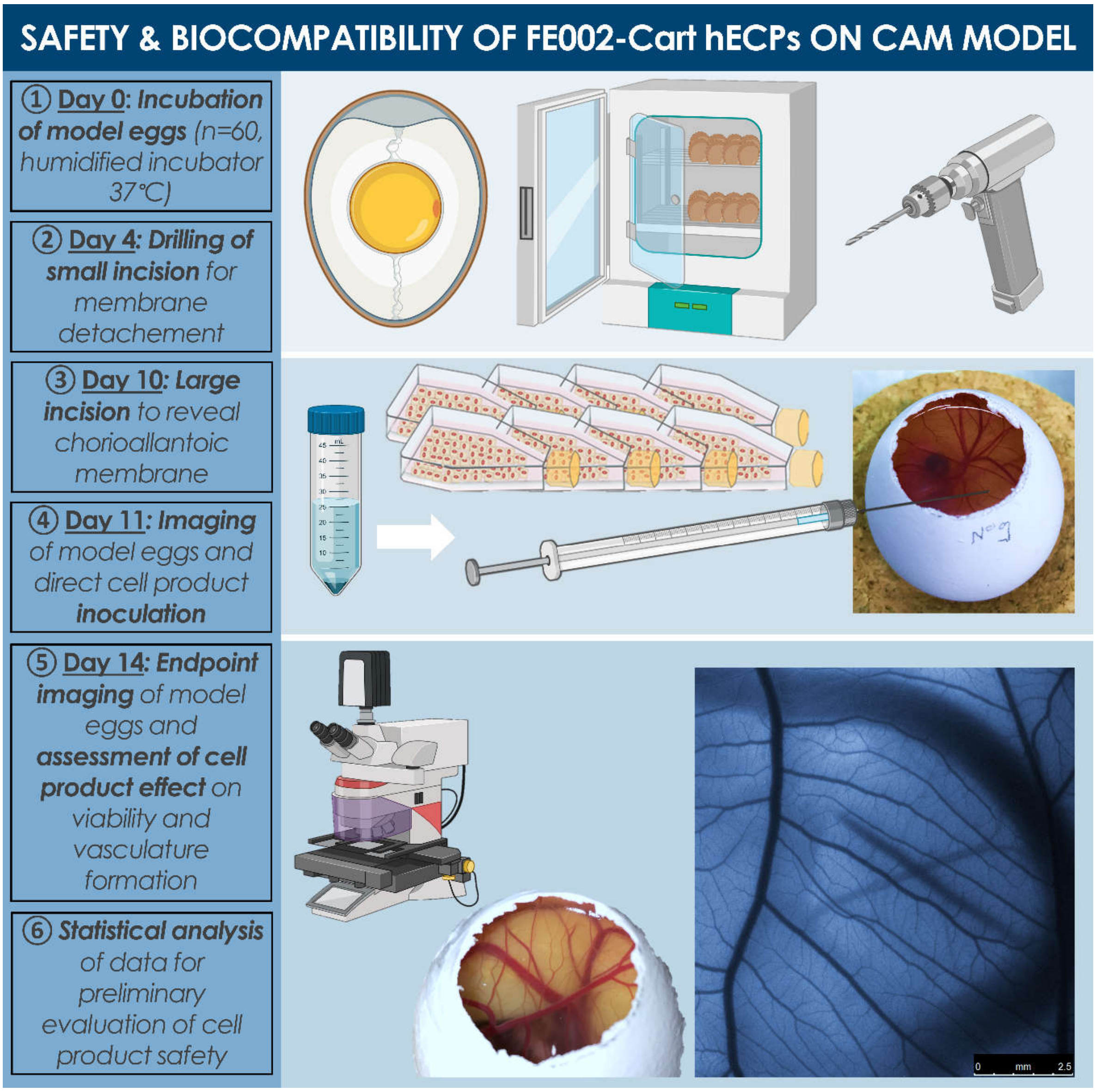

Figure S1. Schematic and graphical overview of the adapted CAM assay for standardized evaluation of hECP biocompatibility. Both embryo viability and quality of vasculature formation served for the endpoint assessment of FPC effects.

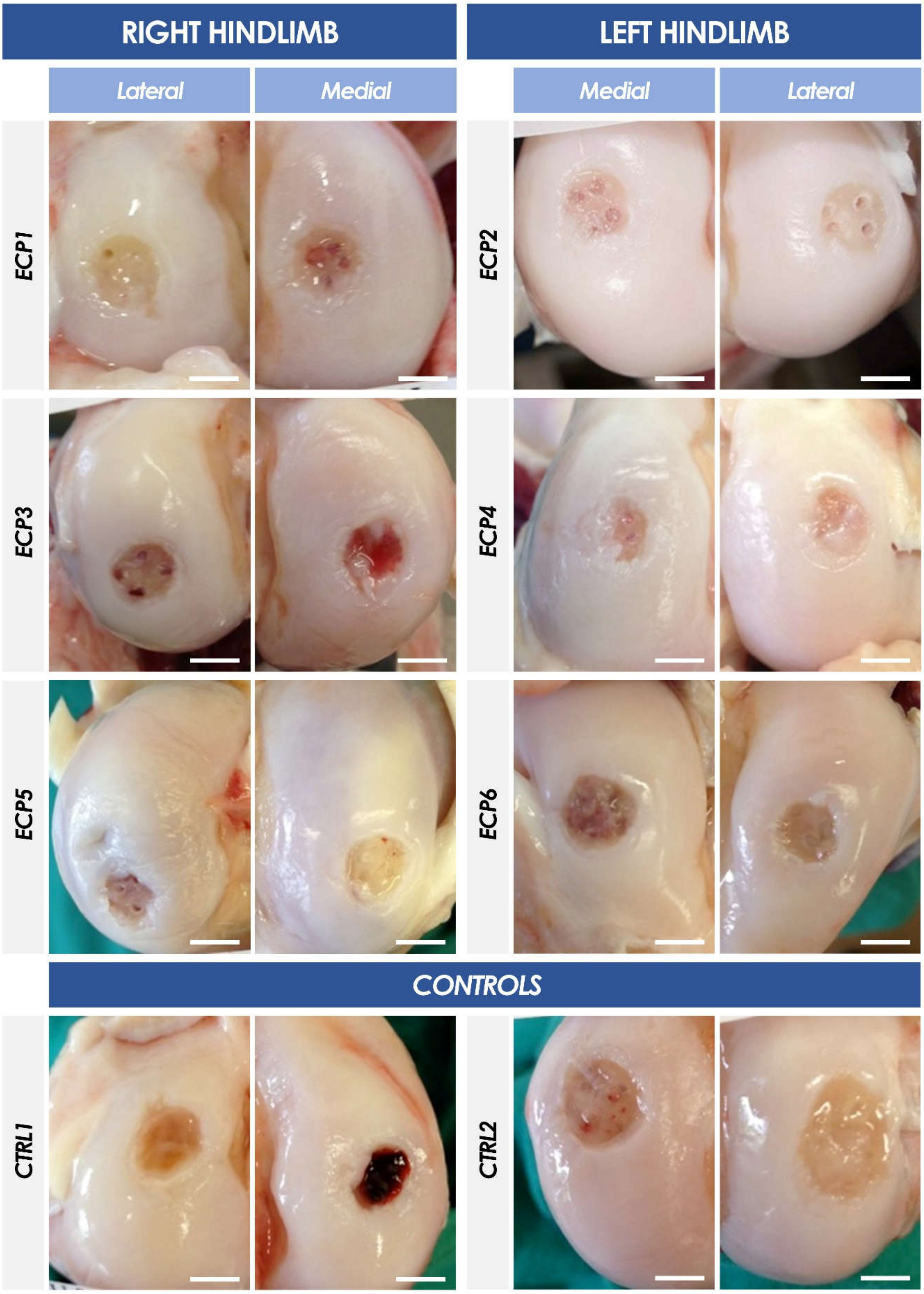

Figure S2. Macroscopic imaging of the harvested stifle joint condyles. Evaluation parameters included the health of surrounding cartilage tissue, the clarity of the defect rim, and the quality of repair tissue. Upon macroscopic evaluation of the harvested joints, the synovial fluid and membrane in ECP1 appeared normal. The cartilage surrounding the defect on the lateral condyle appeared healthy, with a clearly defined defect rim and some apparent resurfacing. The medial condyle however showed evidence of a cobblestone-like cartilage surface, degeneration of cartilage adjacent

to the defect with fissures and cracks, and clearly visible microfracture (MF) drill holes, possibly indicating delamination. ECP2 showed evidence of delamination in defects of both condyles with healthy surrounding cartilage tissue. The lateral condyle defect had a regular rim with medial MF drill holes slightly filled with light tissue and lateral holes unchanged. The medial condyle defect seemed to contain a thin transparent tissue layer through which MF drill holes could still be distinguished. The synovial fluid aspirate from ECP3 appeared bloody, with the synovial membrane showing signs of inflammation, particularly on the medial side. This may have occurred in relation to the secondary patellar lesion observed corresponding to the lesion between the trochlear and weight-bearing region. The cartilage surrounding both implant sites seemed healthy, despite the presence of a transparent tissue film in the lateral defect, visible MF drill holes, and a clear defect rim. The medial defect had a blood-tinged center, no visible MF drill holes, and may have maintained part of the implant in place, with apparent ingrowth of cartilage islets from the defect rim. Despite a lesion in the middle of the trochlear groove and the distal part of the patella, no major signs of synovial membrane inflammation were noted for ECP4, with the synovial fluid appearing as clear and present in normal physiological amounts. Cartilage surrounding both defect sites did not seem macroscopically affected, as they demonstrated a thin membranous tissue present in the lateral defect covering MF drill holes. The medial defect showed evidence of tissue ingrowth covering half of the defect site, with two MF drill holes still apparent and a heterogenous defect rim. The lateral defect in ECP5 had no apparent MF drill holes, had transparent tissue visible above the implanted matrix, with some neocartilage formation, and normal surrounding cartilage tissue. The medial defect however seemed delaminated, with no visible membrane, clearly present MF drill holes, and a degeneration of adjacent cartilage 5 mm away from the defect, evidencing subchondral cyst formation. No patellar erosion or abnormalities in synovial fluid were however noted, indicating that a risk of infection was unlikely. ECP6 presented normal synovial membranes and fluid, a good appearance on both condyles with clear evidence of new cartilage formation, invisible MF drill holes, and a transparent gel-like tissue within both defects, with identifiable small cartilage islets. The synovial fluid in CTRL1 appeared opaque, blood-tinged, and abnormally highly viscous, with apparent blood clots. It was also present in an amount higher than normal physiological levels, which, together with an activated reddened synovial membrane with visibly enlarged vessels, was an indication of inflammation. A tissue layer was however apparent, covering the MF drill holes in the lateral defect. The defect rim was however irregular, with some radiating fissures into what appeared to be an otherwise undisturbed surrounding cartilage tissue. The medial defect however seemed to have deepened into the subchondral bone and formed a large blood clot, along with an irregular defect rim. The synovial fluid in CTRL2 had a higher viscosity and was present in larger amounts than normal but was not blood-tinged. The synovial membrane was however activated. The medial defect showed thin tissue coverage, with three of the four MF drill holes still visible. The surrounding cartilage seemed unchanged, despite a discoloration visible medially. The defect rim was regular. The lateral defect had two of four MF drill holes visible, a tissue layer covering the defect, and a regular rim with small outward fissures. Resurfacing however was almost continuous with surrounding cartilage surfaces and overall cartilage tissue seemed unchanged. A bloody imbibition was nevertheless observed in the lateral part of the lateral condyle, which presented indications of local inflammation. Scale bars = 5 mm.

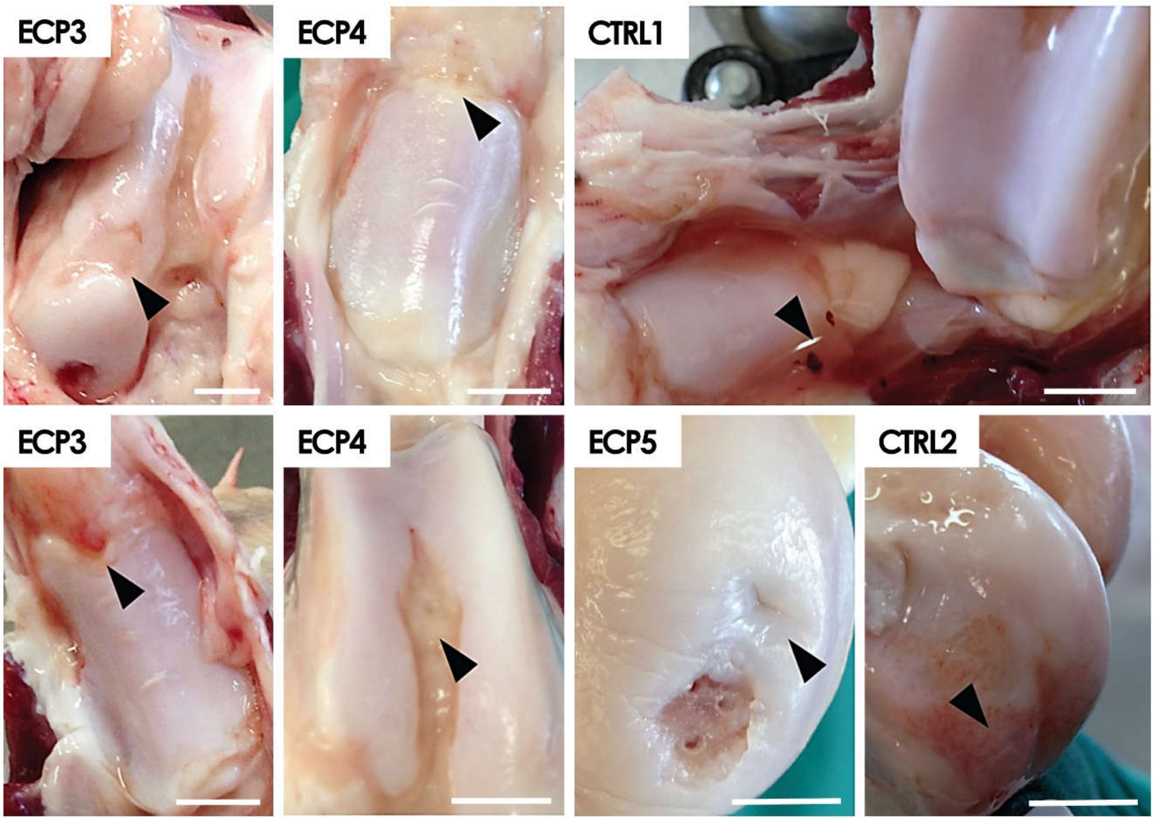

Figure S3. Abnormalities noted during macroscopic evaluations of the harvested stifle joints, secondary to defects. As indicated by the arrowheads, ECP3 and ECP4 presented patellar lesions and other secondary cartilage lesions, ECP5 showed evidence of subchondral cyst formation, secondary to the adjacent defect site. CTRL1 had blood-tinged synovial fluid, with evidence of blood clots. CTRL2 showed evidence of bloody imbibition on the lateral side of the lateral condyle, with evidence of inflammation. Scale bars = 10 mm.

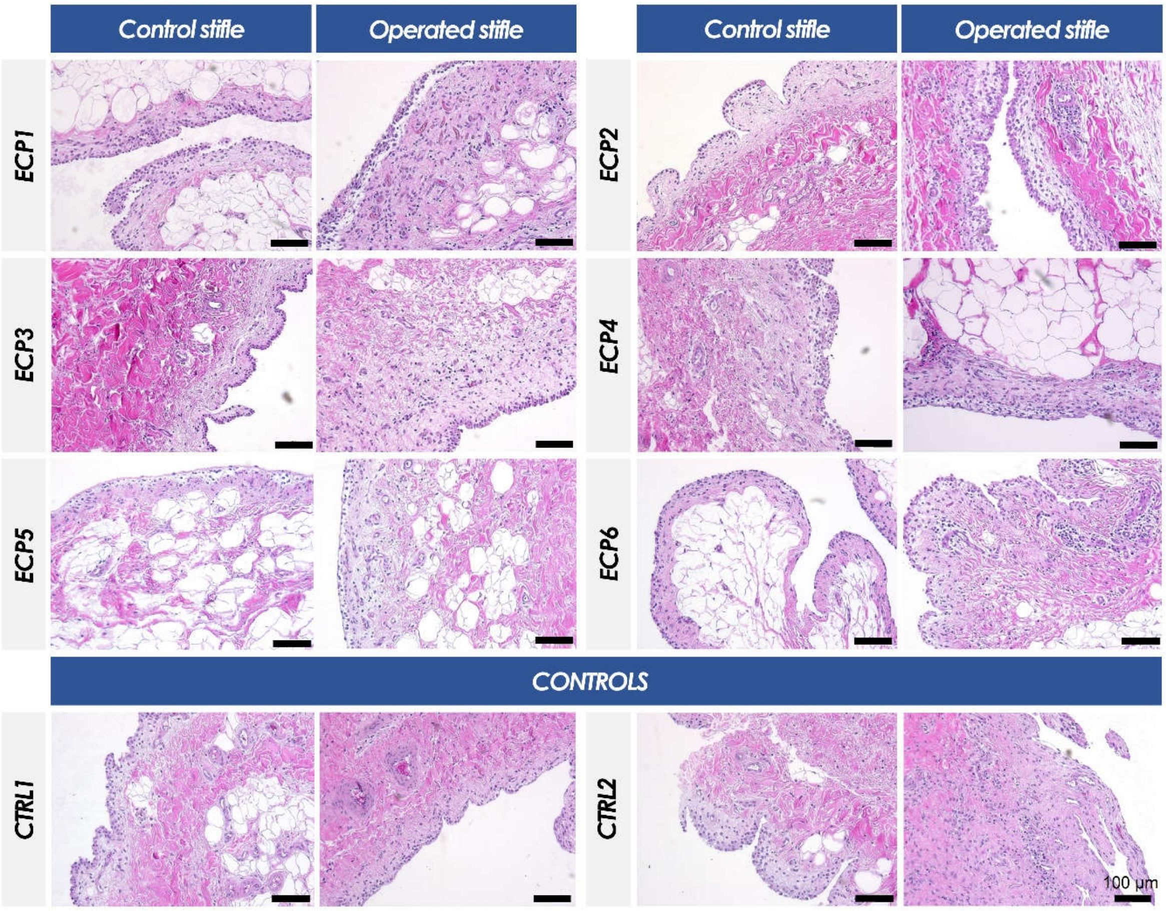

Figure S4. Synovial membrane samples harvested from operated and un-operated contralateral stifles, processed and stained with hematoxylin and eosin, were used to detect indications of synovitis according to two separate scoring scales (i.e., Krenn and modified Rooney score scales). Scale bars = 100 µm.

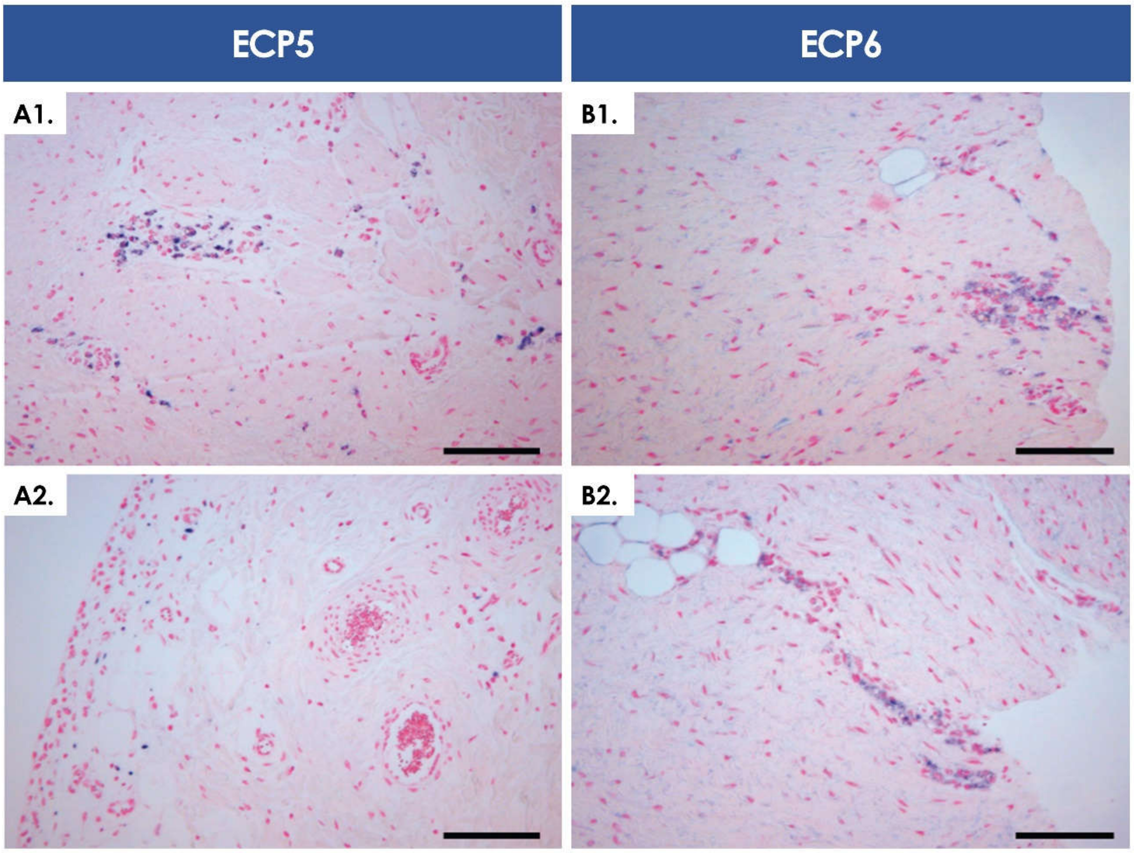

Figure S5. Human ECPs in caprine synovial membrane samples taken from ECP5 and ECP6. Human cells were either organized in clumps together with caprine cells or sparsely distributed within the tissue. Human cells, detected via DNA Alu repeats, appear in dark purple, while caprine cells and tissues were counterstained with Nuclear Fast Red, and appear in various shades of pink. Scale bars = 100 µm.

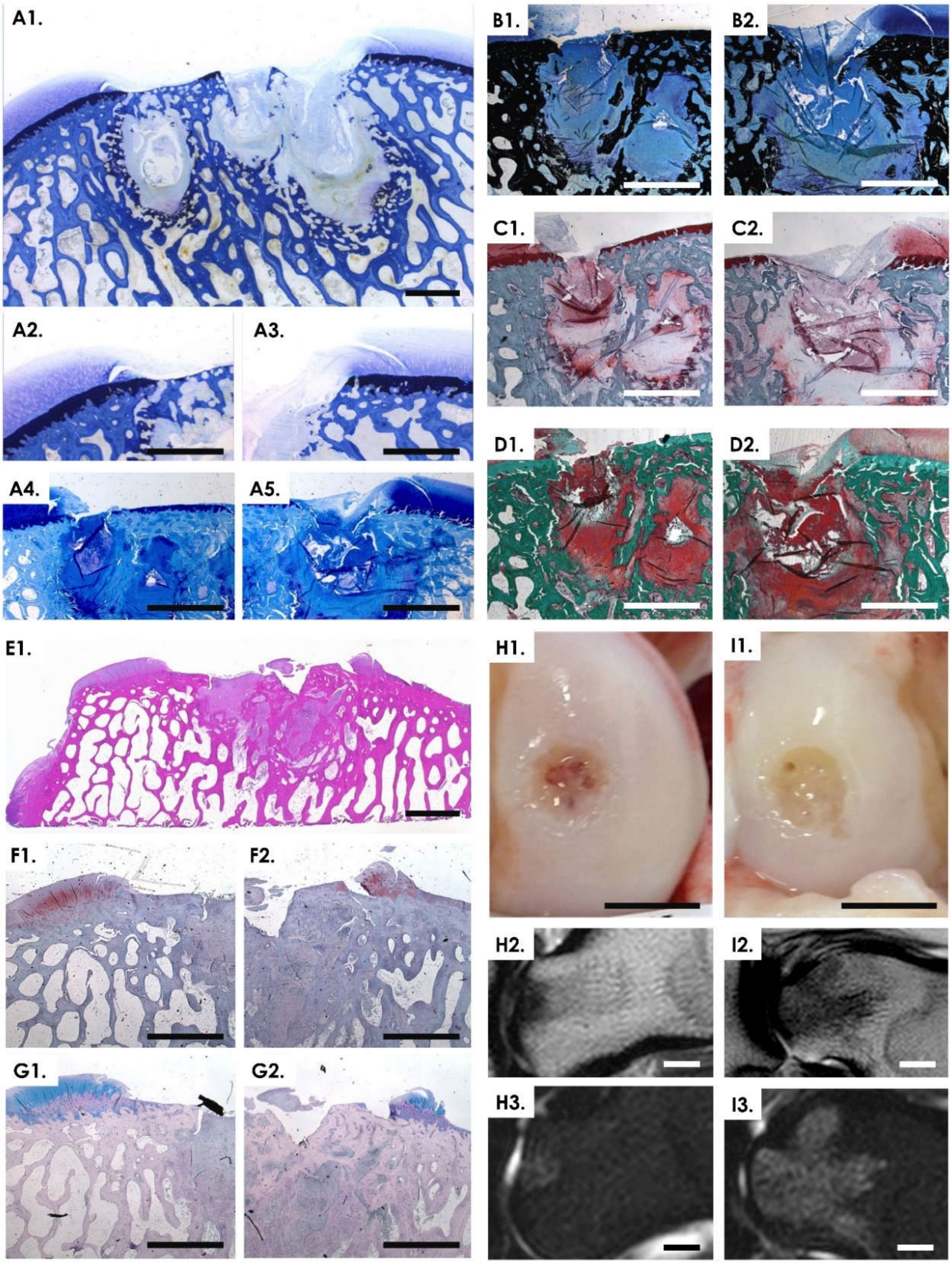

Figure S6. Histological, macroscopic, and MRI imaging mainly performed on the medial condyle from ECP1. (**A-D**) Histological staining of MMA-embedded medial condyle. (**A1-A3**) Toluidine Blue on ground sections. (**A4, A5**) Toluidine blue on thin sections. (**B1, B2**) VonKossa/McNeal stain on thin sections. (**C1, C2**) Safranin-O stain on thin sections. (**D1, D2**) Masson Goldner trichrome stain on thin sections. (**E-G**) Histological staining of paraffin-embedded, decalcified medial condyle. (**E1**) Hematoxylin and eosin. (**F1, F2**) Safranin-O. (**G1, G2**) Alcian Blue staining. (**H1, I1**) Macroscopic imaging of medial and lateral condyles of the right hindlimb, respectively. (**H2, I2**) MRI data for sclerotic bone structure visualization, from the medial and lateral condyles of the right hindlimb, respectively. (**H3, I3**) MRI data for bone marrow edema visualization, from the medial and lateral condyles of the right hindlimb, respectively. Scale bars = 2 mm (A-G); 10 mm (H-I).

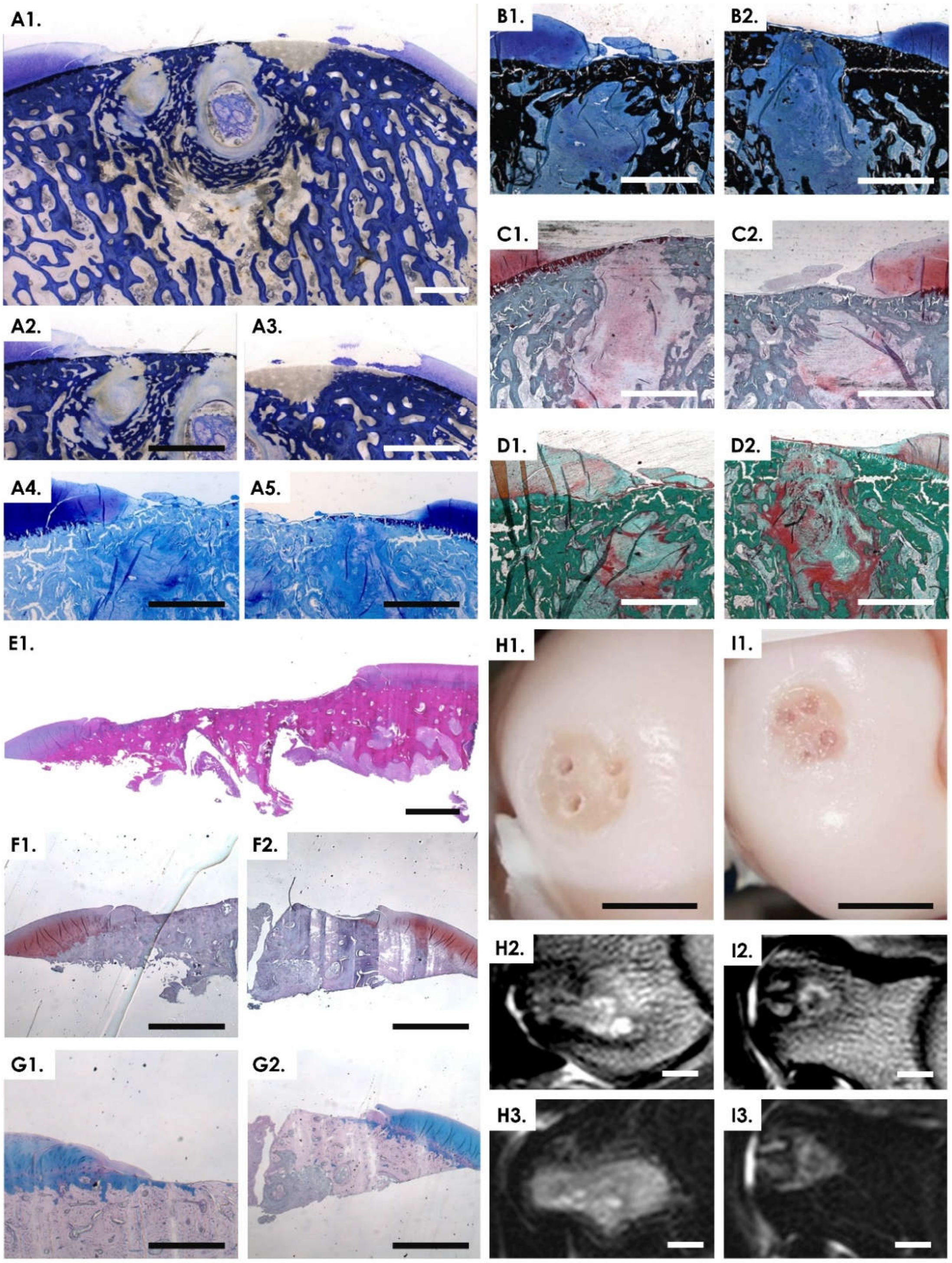

Figure S7. Histological, macroscopic, and MRI imaging mainly performed on the medial condyle from ECP2. (**A-D**) Histological staining of MMA-embedded medial condyle. (**A1-A3**) Toluidine Blue on ground sections. (**A4, A5**) Toluidine blue on thin sections. (**B1, B2**) VonKossa/McNeal stain on thin sections. (**C1, C2**) Safranin-O stain on thin sections. (**D1, D2**) Masson Goldner trichrome stain on thin sections. (**E-G**) Histological staining of paraffin-embedded, decalcified medial condyle. (**E1**) Hematoxylin and eosin. (**F1, F2**) Safranin-O. (**G1, G2**) Alcian Blue staining. (**H1, I1**) Macroscopic imaging of lateral and medial condyles of the left hindlimb, respectively. (**H2, I2**) MRI data for sclerotic bone structure visualization, from the lateral and medial condyles of the left hindlimb, respectively. (**H3, I3**) MRI data for bone marrow edema visualization, from the lateral and medial condyles of the left hindlimb, respectively. Scale bars = 2 mm (A-G); 10 mm (H-I).

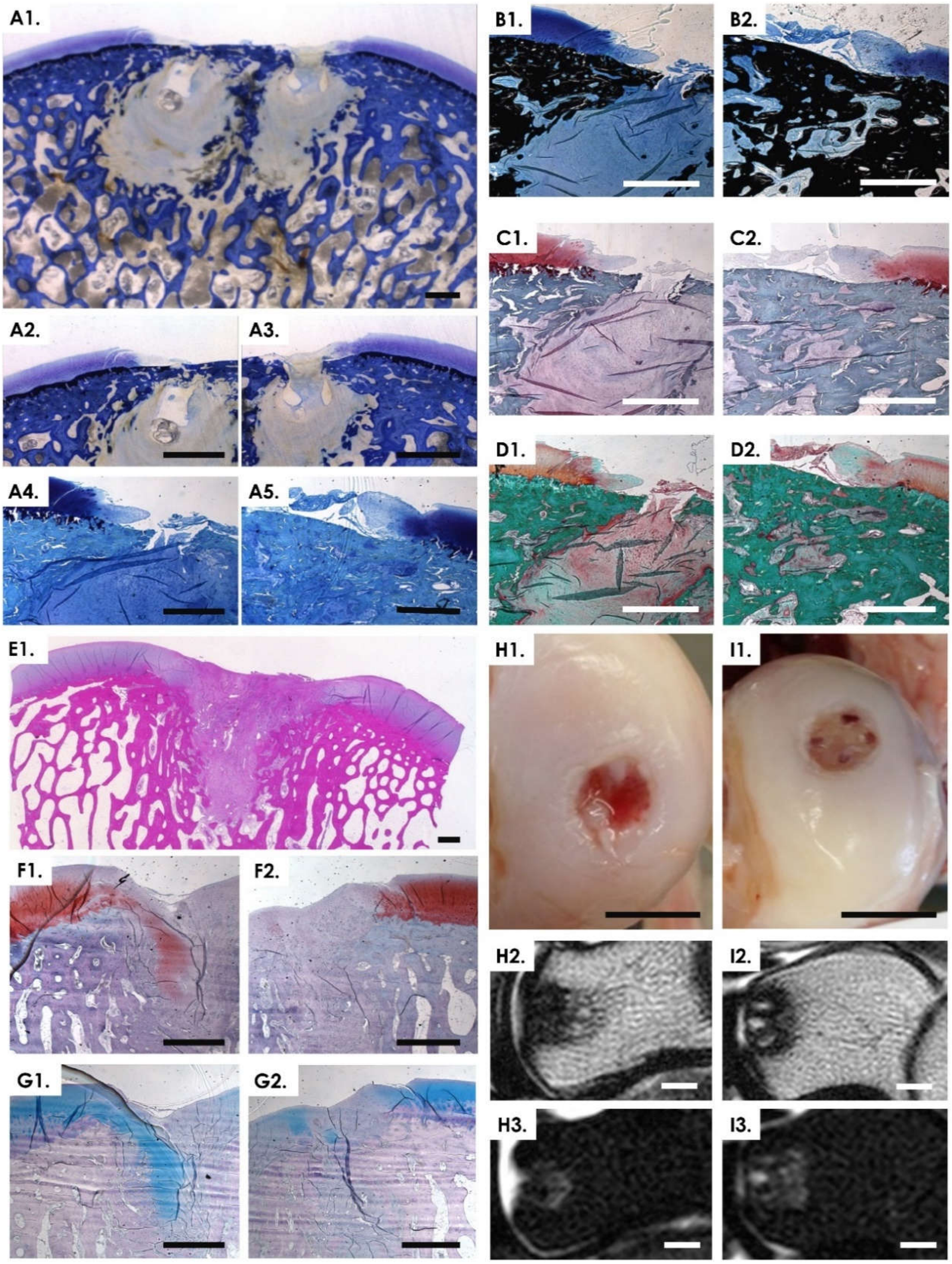

Figure S8. Histological, macroscopic, and MRI imaging mainly performed on the medial condyle from ECP3. (**A-D**) Histological staining of MMA-embedded medial condyle. (**A1-A3**) Toluidine Blue on ground sections. (**A4, A5**) Toluidine blue on thin sections. (**B1, B2**) VonKossa/McNeal stain on thin sections. (**C1, C2**) Safranin-O stain on thin sections. (**D1, D2**) Masson Goldner trichrome stain on thin sections. (**E-G**) Histological staining of paraffin-embedded, decalcified medial condyle. (**E1**) Hematoxylin and eosin. (**F1, F2**) Safranin-O. (**G1, G2**) Alcian Blue staining. (**H1, I1**) Macroscopic imaging of medial and lateral condyles of the right hindlimb, respectively. (**H2, I2**) MRI data for sclerotic bone structure visualization, from the medial and lateral condyles of the right hindlimb, respectively. (**H3, I3**) MRI data for bone marrow edema visualization, from the medial and lateral condyles of the right hindlimb, respectively. Scale bars = 2 mm (A-G); 10 mm (H-I).

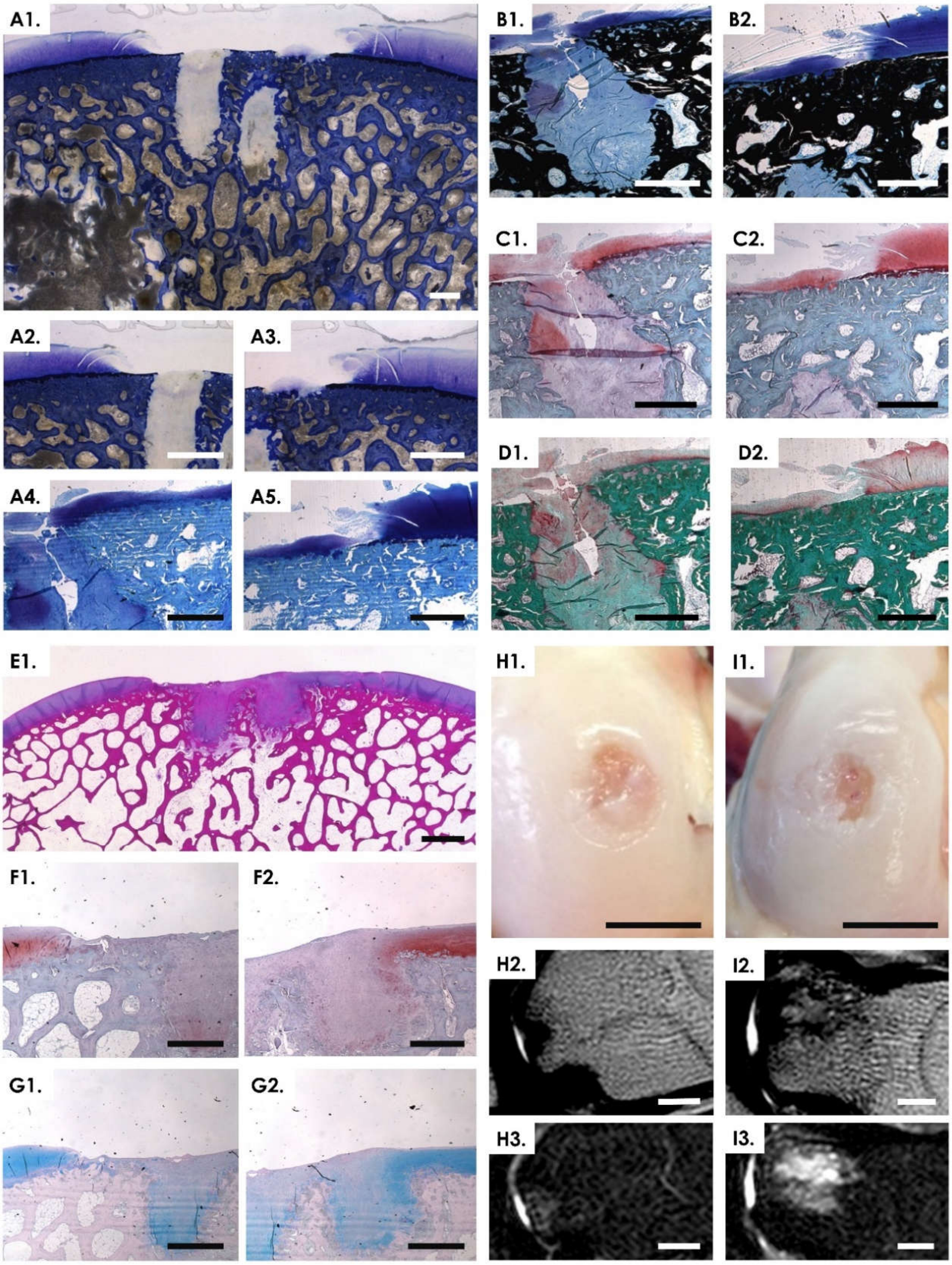

Figure S9. Histological, macroscopic, and MRI imaging mainly performed on the medial condyle from ECP4. (**A-D**) Histological staining of MMA-embedded medial condyle. (**A1-A3**) Toluidine Blue on ground sections. (**A4, A5**) Toluidine blue on thin sections. (**B1, B2**) VonKossa/McNeal stain on thin sections. (**C1, C2**) Safranin-O stain on thin sections. (**D1, D2**) Masson Goldner trichrome stain on thin sections. (**E-G**) Histological staining of paraffin-embedded, decalcified medial condyle. (**E1**) Hematoxylin and eosin. (**F1, F2**) Safranin-O. (**G1, G2**) Alcian Blue staining. (**H1, I1**) Macroscopic imaging of lateral and medial condyles of the left hindlimb, respectively. (**H2, I2**) MRI data for sclerotic bone structure visualization, from the lateral and medial condyles of the left hindlimb, respectively. (**H3, I3**) MRI data for bone marrow edema visualization, from the lateral and medial condyles of the left hindlimb, respectively. Scale bars = 2 mm (A-G); 10 mm (H-I).

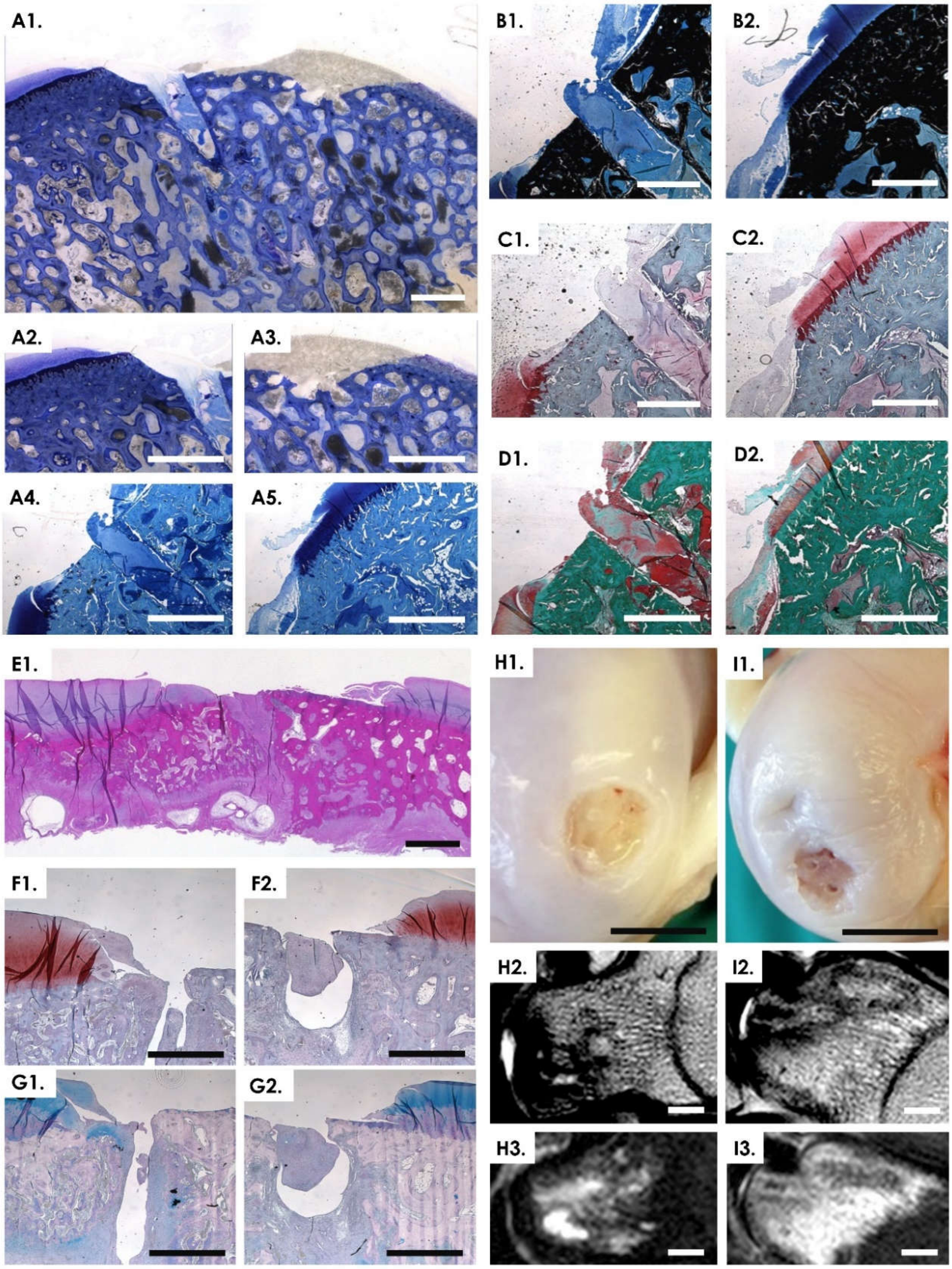

Figure S10. Histological, macroscopic, and MRI imaging mainly performed on the medial condyle from ECP5. (**A-D**) Histological staining of MMA-embedded medial condyle. (**A1-A3**) Toluidine Blue on ground sections. (**A4, A5**) Toluidine blue on thin sections. (**B1, B2**) VonKossa/McNeal stain on thin sections. (**C1, C2**) Safranin-O stain on thin sections. (**D1, D2**) Masson Goldner trichrome stain on thin sections. (**E-G**) Histological staining of paraffin-embedded, decalcified medial condyle. (**E1**) Hematoxylin and eosin. (**F1, F2**) Safranin-O. (**G1, G2**) Alcian Blue staining. (**H1, I1**) Macroscopic imaging of medial and lateral condyles of the right hindlimb, respectively. (**H2, I2**) MRI data for sclerotic bone structure visualization, from the medial and lateral condyles of the right hindlimb, respectively. (**H3, I3**) MRI data for bone marrow edema visualization, from the medial and lateral condyles of the right hindlimb, respectively. Scale bars = 2 mm (A-G); 10 mm (H-I).

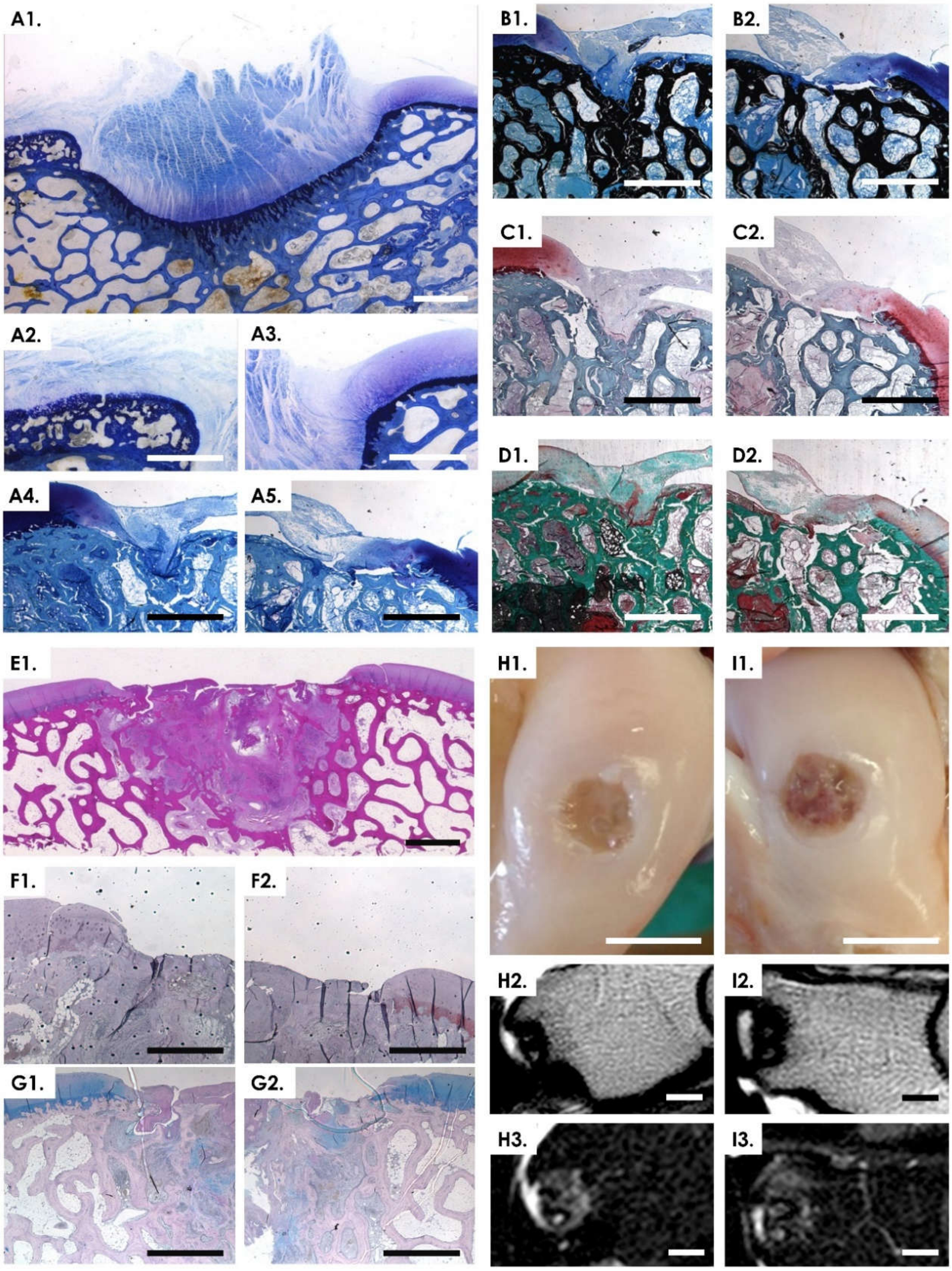

Figure S11. Histological, macroscopic, and MRI imaging mainly performed on the medial condyle from ECP6. (**A-D**) Histological staining of MMA-embedded medial condyle. (**A1-A3**) Toluidine Blue on ground sections. (**A4, A5**) Toluidine blue on thin sections. (**B1, B2**) VonKossa/McNeal stain on thin sections. (**C1, C2**) Safranin-O stain on thin sections. (**D1, D2**) Masson Goldner trichrome stain on thin sections. (**E-G**) Histological staining of paraffin-embedded, decalcified medial condyle. (**E1**) Hematoxylin and eosin. (**F1, F2**) Safranin-O. (**G1, G2**) Alcian Blue staining. (**H1, I1**) Macroscopic imaging of lateral and medial condyles of the left hindlimb, respectively. (**H2, I2**) MRI data for sclerotic bone structure visualization, from the lateral and medial condyles of the left hindlimb, respectively. (**H3, I3**) MRI data for bone marrow edema visualization, from the lateral and medial condyles of the left hindlimb, respectively. Scale bars = 2 mm (A-G); 10 mm (H-I).

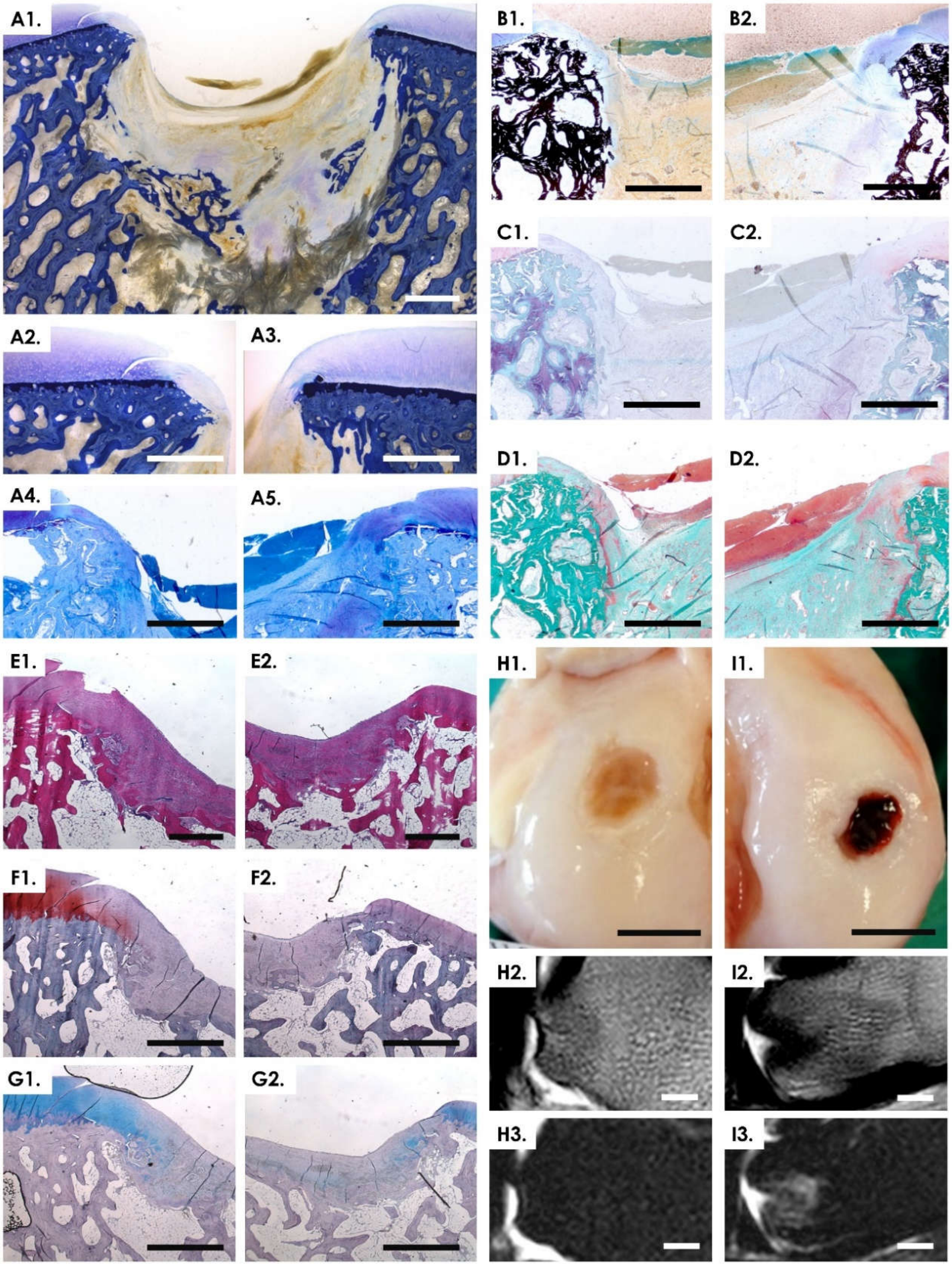

Figure S12. Histological, macroscopic, and MRI imaging mainly performed on the medial condyle from CTRL1. (**A-D**) Histological staining of MMA-embedded medial condyle. (**A1-A3**) Toluidine Blue on ground sections. (**A4, A5**) Toluidine blue on thin sections. (**B1, B2**) VonKossa/McNeal stain on thin sections. (**C1, C2**) Safranin-O stain on thin sections. (**D1, D2**) Masson Goldner trichrome stain on thin sections. (**E-G**) Histological staining of paraffin-embedded, decalcified medial condyle. (**E1**) Hematoxylin and eosin. (**F1, F2**) Safranin-O. (**G1, G2**) Alcian Blue staining. (**H1, I1**) Macroscopic imaging of lateral and medial condyles of the right hindlimb, respectively. (**H2, I2**) MRI data for sclerotic bone structure visualization, from the lateral and medial condyles of the right hindlimb, respectively. (**H3, I3**) MRI data for bone marrow edema visualization, from the lateral and medial condyles of the right hindlimb, respectively. Scale bars = 2 mm (A-G); 10 mm (H-I).

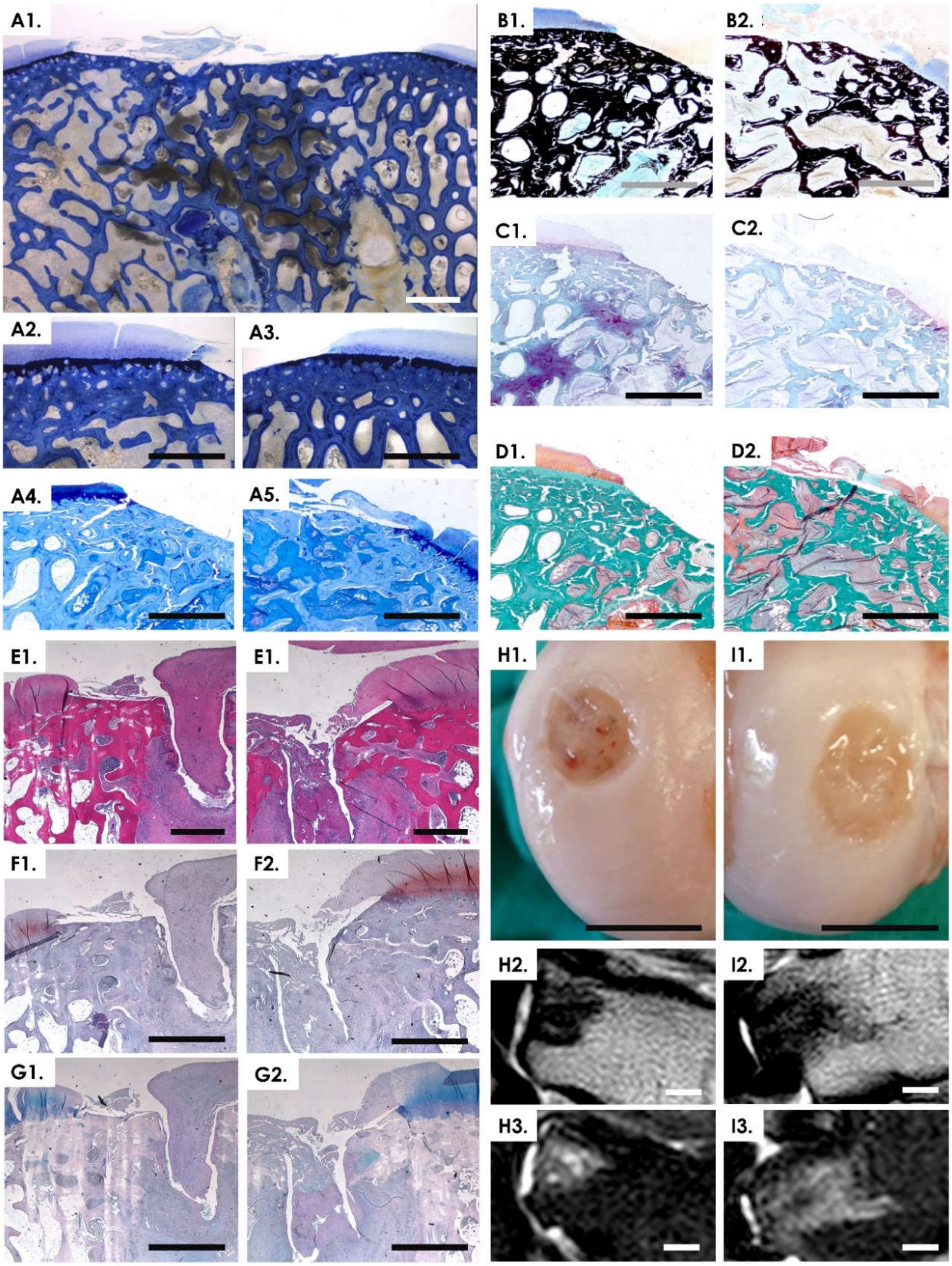

Figure S13. Histological, macroscopic, and MRI imaging mainly performed on the medial condyle from CTRL2. (**A-D**) Histological staining of MMA-embedded medial condyle. (**A1-A3**) Toluidine Blue on ground sections. (**A4, A5**) Toluidine blue on thin sections. (**B1, B2**) VonKossa/McNeal stain on thin sections. (**C1, C2**) Safranin-O stain on thin sections. (**D1, D2**) Masson Goldner trichrome stain on thin sections. (**E-G**) Histological staining of paraffin-embedded, decalcified medial condyle. (**E1**) Hematoxylin and eosin. (**F1, F2**) Safranin-O. (**G1, G2**) Alcian Blue staining. (**H1, I1**) Macroscopic imaging of medial and lateral condyles of the left hindlimb, respectively. (**H2, I2**) MRI data for sclerotic bone structure visualization, from the medial and lateral condyles of the left hindlimb, respectively. (**H3, I3**) MRI data for bone marrow edema visualization, from the medial and lateral condyles of the left hindlimb, respectively. Scale bars = 2 mm (A-G); 10 mm (H-I).

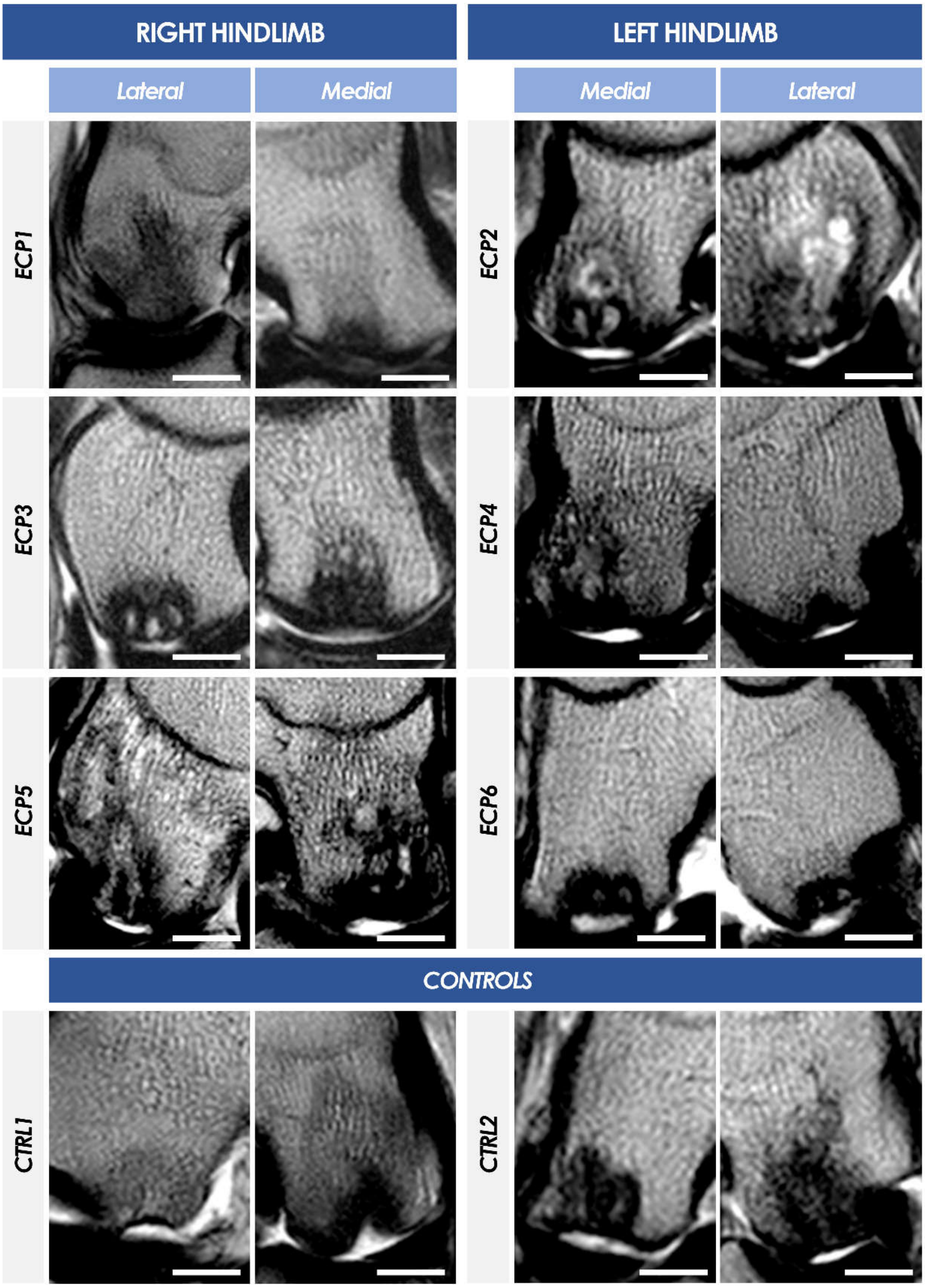

Figure S14. Magnetic resonance imaging of controls and operated joints using the T2-weighted fast spin echo sequence, which detects sclerotic bone structures, as evidenced by the darker reaction signals. In ECP2, the pattern of bone sclerosis was deemed abnormal and may have been indicative of a cyst formation or an infection. The medial condyle in ECP4 unexpectedly showed a severe sclerotic and edema reaction resembling a cyst formation. As expected, the sclerotic reaction in the lateral condyle of ECP5 permeated through the epiphysis. The medial condyle, seemingly stable, showed an intense subchondral reaction. The sclerotic reaction from the medial condyle of CTRL1 confirmed a deepening of the defect. Scale bars = 12 mm.

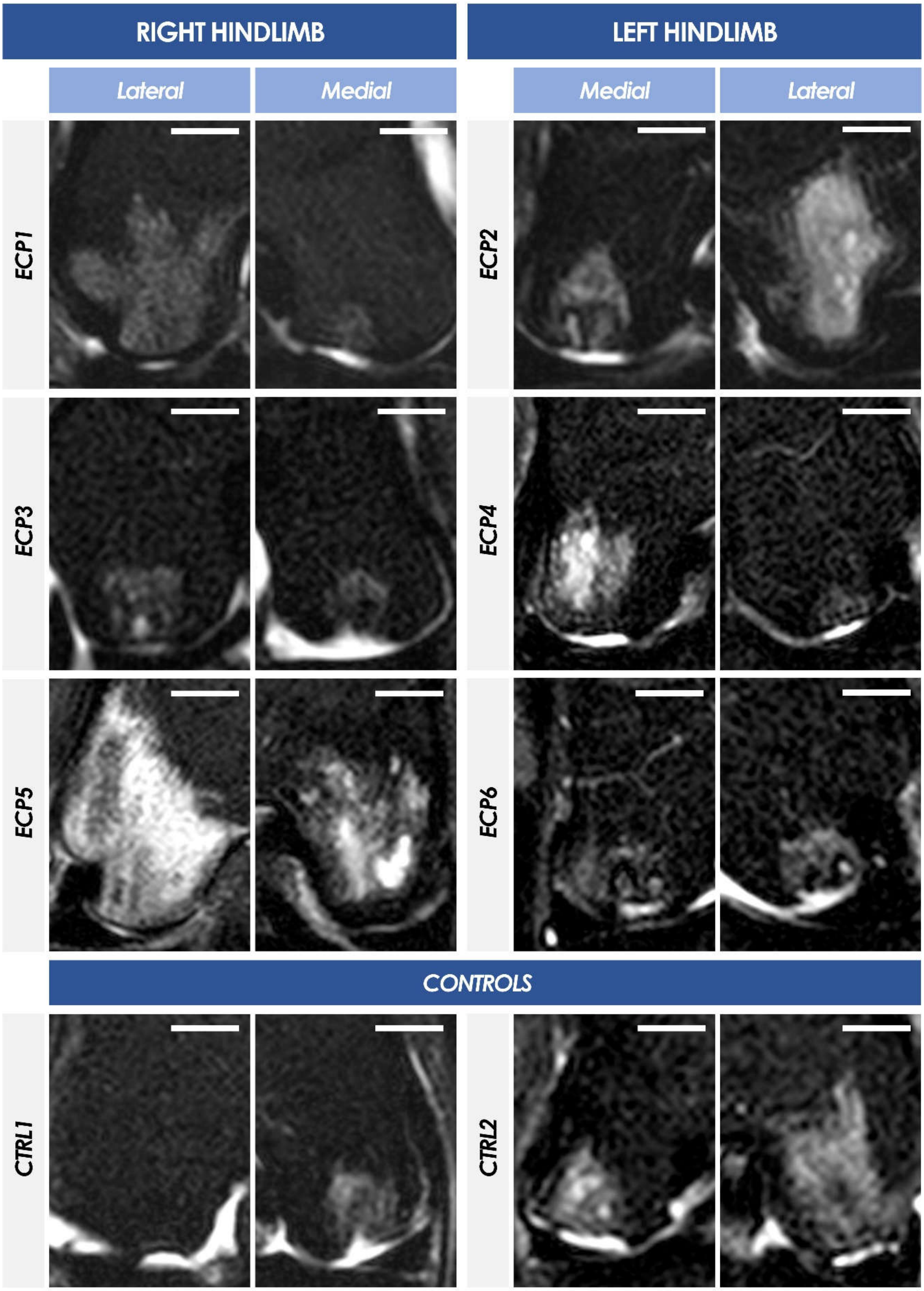

Figure S15. Magnetic resonance imaging of controls and operated joints using the Short T1 Inversion Recovery (STIR) sequence, which detects high water lesions or bone marrow edema, as evidenced by the bright signals. The medial condyle in ECP4 unexpectedly showed a severe sclerotic and edema reaction resembling a cyst formation. Scale bars = 12 mm.

Supplementary Tables

Table S1. Macroscopic scoring data of cartilage defect repair, including surrounding tissues.

Animal	Operated hindlimb	Condyle	Group	Zone 0 [1]	Zone 1 [2]	Zone 2 [3]
ECP1	Right	Lat.	hECP	1	2	0
		Med.	hECP	2	1	0
ECP2	Left	Lat.	hECP	4	1	0
		Med.	hECP	2	1	0
ECP3	Right	Lat.	hECP	2	0	0
		Med.	hECP	3	2	1
ECP4	Left	Lat.	hECP	1	0	0
		Med.	hECP	2	1	0
ECP5	Right	Lat.	hECP	1	0	0
		Med.	hECP	2	2	2
ECP6	Left	Lat.	hECP	1	0	0
		Med.	hECP	1	0	0
CTRL1	Right	Lat.	CTRL	1	2	0
		Med.	CTRL	3	1	0
CTRL2	Left	Lat.	CTRL	1	2	0
		Med.	CTRL	1	2	0

[1] Zone 0, defect center, score values comprised between 0 and 4. [2] Zone 1, defect rim, score values comprised between 0 and 2. [3] Zone 2, adjacent margin, score values comprised between 0 and 2.

Table S2. Synovitis scored according to a score scale described by Krenn *et al.* (2006). Indications of thickening synovial lining cell layer, increased resident cell density, and inflammatory infiltrates were evaluated by three investigators. A consensus score was reported in the table for each parameter.

Animal	Hindlimb	Treatment	Synovial lining cell layer [1]	Resident cell density [2]	Inflammatory infiltrates [3]	Total score [4]
ECP1	Right	hECP	2	2	1	5
	Left	Not Operated	2	1	0	3
ECP2	Left	hECP	2	1	1	4
	Right	Not Operated	1	0	0	1
ECP3	Right	hECP	1	2	2	5
	Left	Not Operated	1	0	0	1
ECP4	Left	hECP	1	2	2	5
	Right	Not Operated	1	2	1	4
ECP5	Right	hECP	0	1	1	2
	Left	Not Operated	0	1	1	2
ECP6	Left	hECP	1	2	2	5
	Right	Not Operated	1	0	0	1
CTRL1	Right	CTRL	1	2	1	4
	Left	Not Operated	1	0	0	1
CTRL2	Left	CTRL	1	3	3	7
	Right	Not Operated	1	0	0	1

[1] Synovial lining cell layer score values comprised between 0 and 3. [2] Resident cell density score values comprised between 0 and 3. [3] Inflammatory infiltrate score values comprised between 0 and 3. [4] Total score values comprised between 0 and 9.

Table S3. Synovitis scored according a modified Rooney score scale, adapted and modified from Yamanaka *et al.* (2010). Indications of synoviocyte hyperplasia, fibrosis, proliferating blood vessels, lymphocyte infiltrate patterns, and aggregates were evaluated by three investigators. A consensus score was reported in the table for each parameter.

Animal	Hindlimb	Treatment	Synoviocyte hyperplasia [1]	Fibrosis [2]	Proliferating blood vessels [3]	Pervascular lymphocyte infiltrates [4]	Lymphocyte focal aggregates [5]	Diffuse lymphocyte infiltrates [6]	Total score [7]
ECP1	Right	hECP	2	3	5	1	0	0	11
	Left	Not Operated	1	0	2	0	0	0	3
ECP2	Left	hECP	2	2	4	1	0	0	9
	Right	Not Operated	0	0	2	0	0	0	2
ECP3	Right	hECP	1	2	4	1	0	1	9
	Left	Not Operated	1	1	2	0	0	0	4
ECP4	Left	hECP	0	0	2	3	0	3	8
	Right	Not Operated	1	1	1	2	0	1	6
ECP5	Right	hECP	0	2	5	0	0	1	8
	Left	Not Operated	0	0	2	2	0	0	4
ECP6	Left	hECP	0	1	4	3	0	1	9
	Right	Not Operated	0	0	2	0	0	0	2
CTRL1	Right	CTRL	0	3	4	0	0	0	7
	Left	Not Operated	0	1	4	0	0	0	5
CTRL2	Left	CTRL	1	3	2	0	0	0	6
	Right	Not Operated	0	0	2	0	0	0	2

[1] Synoviocyte hyperplasia score values comprised between 0 and 5. [2] Fibrosis score values comprised between 0 and 5. [3] Proliferating blood vessel score values comprised between 0 and 5. [4] Pervascular lymphocyte aggregate score values comprised between 0 and 4. [5] Lymphocyte focal aggregate score values comprised between 0 and 5. [6] Diffuse lymphocyte infiltrate score values comprised between 0 and 5. [7] Total score values comprised between 0 and 29.

Table S4. A modified O'Driscoll scoring was performed to evaluate cartilage repair in the center of the defects (i.e., Zone 0).

Animal	Operated hindlimb	Condyle	Group	Cellular morphology [1]	Safranin-O staining of matrix [2]	Surface coverage [3]	Thickness [4]	Total score [5]
ECP1	Right	Lat.	hECP	3	3	3	3	12
		Med.	hECP	1	3	3	3	10
ECP2	Left	Lat.	hECP	3	3	3	3	12
		Med.	hECP	1	3	0	3	7
ECP3	Right	Lat.	hECP	1	3	1	2	7
		Med.	hECP	1	3	0	1	5
ECP4	Left	Lat.	hECP	1	3	0	2	6
		Med.	hECP	1	0	0	2	3
ECP5	Right	Lat.	hECP	1	3	0	2	6
		Med.	hECP	3	3	0	3	9
ECP6	Left	Lat.	hECP	3	3	3	3	12
		Med.	hECP	1	3	0	1	5
CTRL1	Right	Lat.	CTRL	3	3	0	3	9
		Med.	CTRL	1	3	0	3	7
CTRL2	Left	Lat.	CTRL	3	3	3	2	11
		Med.	CTRL	1	3	2	3	9

[1] Cellular morphology score values comprised between 0 and 3. [2] Safranin-O staining of the matrix score values comprised between 0 and 3. [3] Surface coverage score values comprised between 0 and 3. [4] Thickness score values comprised between 0 and 3. [5] Total score values comprised between 0 and 12.

Table S5. A modified O'Driscoll scoring was performed to evaluate cartilage repair in the defect rim (i.e., Zone 1).

Animal	Operated hindlimb	Condyle	Group	Cellular morphology [1]	Safranin-O staining of matrix [2]	Surface coverage [3]	Thickness [4]	Bonding to adjacent cartilage [5]	Total score [6]
ECP1	Right	Lat.	hECP	1	2	1	1	0	5
		Med.	hECP	3	3	3	3	2	14
ECP2	Left	Lat.	hECP	1	3	3	3	2	12
		Med.	hECP	1	1	0	1	0	3
ECP3	Right	Lat.	hECP	1	1	0	1	0	3
		Med.	hECP	1	1	0	1	0	3
ECP4	Left	Lat.	hECP	1	0	0	0	1	2
		Med.	hECP	1	3	0	2	0	6
ECP5	Right	Lat.	hECP	1	3	0	3	0	7
		Med.	hECP	1	2	2	2	1	8
ECP6	Left	Lat.	hECP	1	1	0	2	1	5
		Med.	hECP	1	2	0	1	0	4
CTRL1	Right	Lat.	CTRL	1	1	0	3	0	5
		Med.	CTRL	1	2	0	2	0	5
CTRL2	Left	Lat.	CTRL	1	3	0	1	1	6
		Med.	CTRL	2	3	3	3	2	13

[1] Cellular morphology score values comprised between 0 and 3. [2] Safranin-O staining of the matrix score values comprised between 0 and 3. [3] Surface coverage score values comprised between 0 and 3. [4] Thickness score values comprised between 0 and 3. [5] Bonding to adjacent cartilage score values comprised between 0 and 2. [6] Total score values comprised between 0 and 14.

Table S6. Scoring according to the Little scale was performed to evaluate the state of cartilage and subchondral tissue present around the defect in the adjacent margin (i.e., Zone 2).

Animal	Operated hindlimb	Condyle	Group	Structure [1]	Cellularity [2]	Cell cloning [3]	Territorial Toluidine Blue staining [4]	Interterritorial Toluidine Blue staining [5]	Tidemark/Calcification Cartilage/Subchondral bone [6]	Total score [7]
ECP1	Right	Lat.	hECP	5	1	4	2	1	0	13
		Med.	hECP	5	1	4	1	1	0	12
ECP2	Left	Lat.	hECP	6	3	3	1	2	1	16
		Med.	hECP	2	1	3	1	1	0	8
ECP3	Right	Lat.	hECP	5	1	4	1	1	2	14
		Med.	hECP	1	2	3	1	1	1	9
ECP4	Left	Lat.	hECP	6	1	1	1	1	3	13
		Med.	hECP	5	1	3	0	0	1	10
ECP5	Right	Lat.	hECP	5	1	4	0	1	0	11
		Med.	hECP	6	1	4	1	1	3	16
ECP6	Left	Lat.	hECP	6	1	3	1	1	3	15
		Med.	hECP	4	1	4	1	1	0	11
CTRL1	Right	Lat.	CTRL	0	1	4	0	0	0	5
		Med.	CTRL	2	1	4	1	2	0	10
CTRL2	Left	Lat.	CTRL	6	1	3	1	1	0	12
		Med.	CTRL	4	2	4	3	3	0	16

[1] Structure score values comprised between 0 and 10. [2] Cellularity score values comprised between 0 and 4. [3] Cell cloning score values comprised between 0 and 4. [4] Territorial Toluidine Blue staining score values comprised between 0 and 4. [5] Interterritorial Toluidine Blue staining score values comprised between 0 and 4. [6] Score values comprised between 0 and 3. [7] Total score values comprised between 0 and 29.

Table S7. Scoring according to the Little scale was performed to evaluate the state of cartilage and subchondral tissue present around the defect in the distal margin (i.e., Zone 3).

Animal	Operated hindlimb	Condyle	Group	Structure [1]	Cellularity [2]	Cell cloning [3]	Territorial Toluidine Blue staining [4]	Interterritorial Toluidine Blue staining [5]	Tidemark/Calcification Cartilage/Subchondral bone [6]	Total score [7]
ECP1	Right	Lat.	hECP	0	0	0	0	0	0	0
		Med.	hECP	0	1	3	0	0	0	4
ECP2	Left	Lat.	hECP	0	0	0	0	0	1	1
		Med.	hECP	0	0	0	0	0	0	0
ECP3	Right	Lat.	hECP	0	0	0	0	0	0	0
		Med.	hECP	0	1	1	0	0	3	5
ECP4	Left	Lat.	hECP	0	1	2	1	0	3	7
		Med.	hECP	0	0	0	0	0	0	0
ECP5	Right	Lat.	hECP	0	0	0	0	0	0	0
		Med.	hECP	0	0	0	0	0	1	1
ECP6	Left	Lat.	hECP	0	1	0	0	0	2	3
		Med.	hECP	0	0	1	0	0	0	1
CTRL1	Right	Lat.	CTRL	0	0	0	0	0	1	1
		Med.	CTRL	0	1	1	0	1	0	3
CTRL2	Left	Lat.	CTRL	0	0	0	1	1	0	2
		Med.	CTRL	0	0	0	0	0	0	0

[1] Structure score values comprised between 0 and 10. [2] Cellularity score values comprised between 0 and 4. [3] Cell cloning score values comprised between 0 and 4. [4] Territorial Toluidine Blue staining score values comprised between 0 and 4. [5] Interterritorial Toluidine Blue staining score values comprised between 0 and 4. [6] Score values comprised between 0 and 3. [7] Total score values comprised between 0 and 29.

Table S8. MRI scoring data. Condyle defects were graded according to an adapted score scale to assess the extent of subchondral bone sclerosis and bone marrow edema, as well as the depth and width of the reactions. The size of subchondral defects was also measured based on MRI data. Score scale: No Finding (0), Mild (1), Moderate (2), Severe (3), and Profound (4). Lateral condyle (Lat.), Medial condyle (Med.).

Animal	Operated Hindlimb	Condyle	Group	Subchondral bone sclerosis			Bone marrow edema			Subchondral defect
				Score [1]	Width [mm]	Depth [mm]	Score [1]	Width [mm]	Depth [mm]	Size [mm]
ECP1	Right	Lat.	hECP	3	13.74	16.77	3	15.41	19.01	5.20
		Med.	hECP	2	7.20	13.37	0	0.00	0.00	5.00
ECP2	Left	Lat.	hECP	4	11.42	18.45	4	9.64	19.29	5.87
		Med.	hECP	3	7.05	10.85	2	7.31	9.97	6.47
ECP3	Right	Lat.	hECP	3	8.85	6.47	2	6.60	7.53	6.30
		Med.	hECP	2	7.06	9.27	1	6.19	7.26	7.15
ECP4	Left	Lat.	hECP	1	3.90	2.77	0	0.00	0.00	5.27
		Med.	hECP	3	8.88	14.12	3	13.71	16.35	7.43
ECP5	Right	Lat.	hECP	3	19.54	23.97	4	24.73	19.97	5.56
		Med.	hECP	4	16.09	19.69	3	15.70	20.66	7.35
ECP6	Left	Lat.	hECP	1	5.79	3.88	1	6.38	6.29	6.85
		Med.	hECP	1	8.48	6.20	1	6.48	6.62	5.93
CTRL1	Right	Lat.	CTRL	2	10.26	10.17	0	0.00	0.00	5.05
		Med.	CTRL	3	13.79	19.79	2	7.29	9.81	9.51
CTRL2	Left	Lat.	CTRL	2	12.38	11.91	2	10.11	13.37	5.89
		Med.	CTRL	2	7.55	8.36	2	6.67	8.88	6.25

[1] Score values comprised between 0 and 4.

Supplementary Methods

Primary hECP non-enzymatic isolation and PCB establishment

Specific anonymized fetal tissue fragments were made available in individual containers by the hospital pathology department, fully immersed in transport medium (i.e., phosphate-buffered saline, PBS CHUV 1X; NaCl 6.8 g/L, Na_2HPO_4 1.5 g/L, KH_2PO_4 0.4 g/L, N°100 0 324, Laboratorium Dr. G. Bichsel AG, Switzerland). The tissues were rapidly transferred (i.e., 4 °C conservation in insulated containers) to a dedicated and accredited hospital facility for bioprocessing and culture initiation. FE002-Cart cell type processing and characterization was summarily described elsewhere by Darwiche *et al.* (2012). Briefly, approximately 5 mm^3 of epiphyseal cartilage (i.e., from the proximal ulnar epiphysis) were made available from the FE002 donation, were washed in conserved PBS containing 1 % penicillin-streptomycin (N°15140-122, Gibco™, UK), and were dissected into small fragments. The fragments were then washed thrice for a total of 15 min in conserved PBS. To perform cell isolation and initiation of adherent cell culture of hECPs, independent tissue sections were then further dissected and minced into < 0.3 mm^3 fragments and placed in 10 cm diameter cell culture Petri dishes (N°353003, Falcon®, USA). The culture Petri dishes had been extemporaneously and deeply scored with a scalpel in a checkerboard pattern. Isolated tissue fragments were placed and attached along the scored plastic regions to favor adherent cell culture initiation. From the original 5 mm^3 tissue biopsy, 6 Petri dishes were planted with homogeneously distributed whole tissue fragments (~ 6-8 fragments/dish). A relatively small volume of warmed (i.e., 37 °C) liquid cell growth medium was dispensed by pipet around each fragment to reduce the probability of tissue flotation during the first 24-hour period. The vessels were stacked and were incubated in a 37 °C ± 2 °C humidified atmosphere with 5 % ± 0.5 % CO_2 and 80 % ± 10 % relative humidity. The initial cell growth medium was composed of Dulbecco's Modified Eagle Medium (DMEM) containing 25 mM dextrose, 1 mM sodium pyruvate, and L-glutamine (DMEM 1X, N°41966-029, Gibco™, USA) supplemented with 10 % v/v of clinical-grade fetal bovine serum (FBS, N°10101145, Invitrogen, USA). Following the first 24 hours of culture, 10 additional mL of initial cell growth medium were carefully dispensed by pipet in each vessel, before re-incubation. The cell growth medium was exempt of antibiotic supplementation and was thereafter renewed every other day. The first cell outgrowth emitting from the tissue fragments was observed as early as 72 hours after culture initiation (i.e., planting of vessels). Cell cultures were regularly microscopically assessed to verify adequate morphology or growth and to exclude contamination. Towards the end of the initial culture period, abundant fibroblast-type hECPs had migrated outwards from the original tissue fragments. Cell outgrowth was allowed to attain 90-95 % relative confluency before harvest of Passage 0 cells. Once optimal banking confluency (i.e., 95 %, assessed by contrast phase microscopy) was attained for these primary cultures (i.e., 12 days), each vessel was rid of the spent growth medium, delicately rinsed twice with PBS, and monolayer cultures were submitted to trypsinization (trypsin-EDTA: 0.05 % trypsin-ethylenediaminetetraacetic acid, N°25300-054, Gibco™, USA). After complete cellular detachment (i.e., confirmed by microscopy), the resulting cell suspensions were diluted with equal volumes of initial growth medium to ensure trypsin inactivation. Cells were enumerated (i.e., total and viable relative cell counts) using hemocytometers and Trypan Blue exclusion dye (Sigma-Aldrich®, USA). The pooled cell suspensions were then centrifuged at 230 ± 10 x *g* at ambient temperature for 15 minutes. The cells were then resuspended in fresh, sterile, and warmed complete medium and distributed into one hundred T75 sterile vented cell culture flasks (Nunc®, N°153732, USA) at a viable seeding density of $1.5 \times 10^3 \pm 100$ cells/cm^2, to allow expansion of Passage 1 cells. Complete medium was composed of DMEM, FBS, and 200-times concentrated L-glutamine (N°25030-024, Gibco™, USA) in 100:10:1 volumetric proportion. Final L-glutamine concentration was 5.97 mM and complete medium was exempt of antibiotic supplementation. The cell-seeded culture vessels were stacked in flights of five and incubated as previously mentioned. The complete medium (i.e., 15 mL/flask) was renewed every two days until the confluency attained 95 %. Cells were regularly microscopically assessed to verify adequate morphology, growth characteristics, and to exclude contamination. Confluent cells (i.e., Passage 1) were then harvested by trypsinization and enumerated, as described hereabove. The cell suspension was centrifuged at ambient temperature for 15 min at 230 ± 10 x *g*. After supernatant removal, the cell pellet

was resuspended in an *ad hoc* cryopreservation solution composed of DMEM, FBS, and dimethyl sulfoxide (DMSO, N°D-2438, Sigma-Aldrich®, USA) in 67.5 %; 27.5 %; 5.0 % proportion to attain final viable cellular densities ranging from 2 x 10^6 to 10^7 viable cells/mL. Cell suspensions were then homogenously dispensed and frozen in individual aliquots (i.e., 1.0 to 1.1 mL/vial, individually labelled 1.8 mL cryovials, N°366656, Nunc®, USA) using Nalgene® Mr. Frosty® Cryo 1°C Freezing Containers (Nalgene®, UK), which were rapidly placed in an ultra-low temperature (i.e., -80 °C) freezer to achieve a -1 °C/minute rate of cooling and a corresponding freezing curve. After 12 hours in the freezing containers, the cryovials were rapidly transferred to the liquid nitrogen vapor phase (i.e., −165 °C) of a quarantine storage tank. The vials were subsequently stored in separate locked level-alarm fitted long-term Dewar storage tank containers in several locations to mitigate risks of destruction. This cryopreserved material was defined as the FE002-Cart Parental Cell Bank (PCB, Passage 1). All entries and vial movements were logged in a central file for inventory and traceability purposes.

Supplementary Data

Mechanical indentation of harvested tissues

Mechanical indentation was performed at the "Centre Suisse d'Electronique et de Microtechnique" (Neuchatel, Switzerland) on condyle samples immersed in phosphate buffered saline for transport after harvest. A spherical ruby probe tip, 1 mm in diameter, was used (CSM Instruments Bioindenter). Regions of cartilage tissue surrounding the defects were subjected to a 30 second loading ramp up to a load of 1 mN, which was held for another 120 seconds and then released over a 30 second unloading ramp. The same loading protocol was used to indent repair tissue within the defects with a maximal load of 100 μN to avoid signal saturation. The Hertz model was used on the loading ramp to estimate Young's modulus. Tests were performed in triplicate, separated by 100 μm within a given region of interest. Cartilage tissue was tested 5 - 10 mm away from the defect site.

Results of biomechanical analysis

High variability in outcomes was observed in the measured stiffness of studied cartilage tissues, ranging from 119 ± 4 kPa to 637 ± 36 kPa, with lateral condyles showing a slight trend toward higher stiffness (Figure SD1). This corroborated notes from the veterinary surgeon about the normally stiffer lateral condyle cartilage, as compared to that found in the medial condyle. The range was also very variable when measuring the stiffness of repair tissue, going from 10 ± 2 kPa to 71 ± 11 kPa. We were unable to measure the stiffness of repair tissue in several samples, as it appeared that the defect site was filled with a loose, soft gel-like substance (Figure SD1).

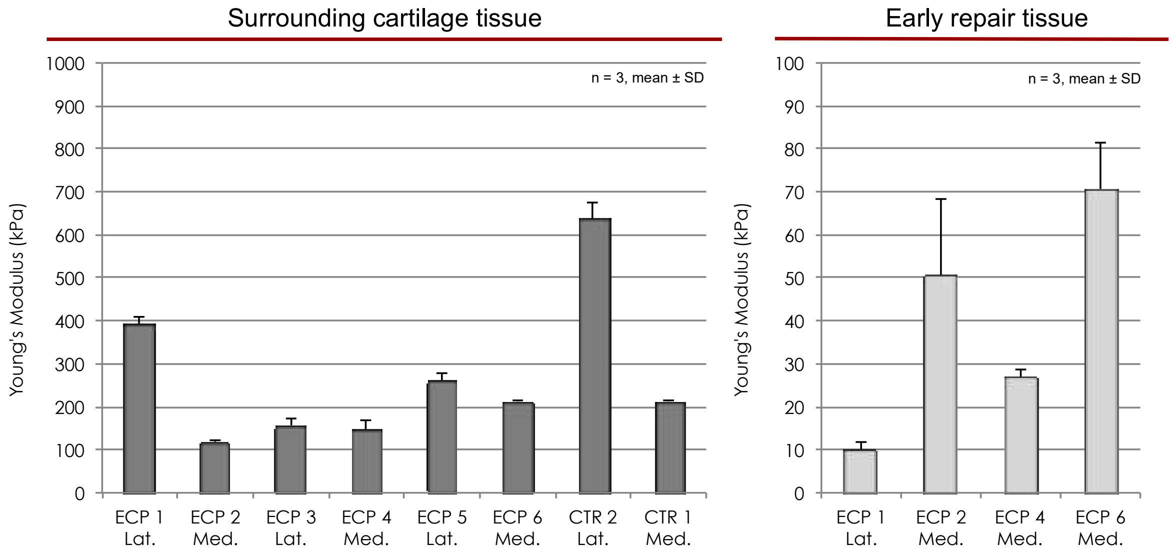

Figure SD1: Mechanical indentation performed on harvested cartilage tissue and early repair tissue in condyle samples immersed in phosphate buffered saline. The loading protocol increased load on the sample for 30 seconds, maintained a plateau for 120 seconds and then receded for another 30 seconds. The maximal load used was 1 mN for cartilage tissue and 100 μN for early repair tissue. Young's modulus is represented as an average of three measurements with the error bars reporting the corresponding standard deviation.
